# Climate change influences on the potential geographic distribution of the invasive Asian longhorned tick, *Haemaphysalis longicornis*

**DOI:** 10.1038/s41598-025-86205-6

**Published:** 2025-01-17

**Authors:** Mohammed Okely, Ze Chen, Eslam Adly, Mahmoud Kamal

**Affiliations:** 1 Entomology Department, Faculty of Science, Ain Shams University, Abbassia, Cairo, 11566 Egypt; 2 Hebei Key Laboratory of Animal Physiology, Biochemistry, and Molecular Biology, College of Life Sciences, Hebei Normal University, Shijiazhuang, 050024 Hebei P.R. China

**Keywords:** Climate change, Hard ticks, Vector invasion, *Haemaphysalis longicornis*, Computational biology and bioinformatics, Ecology, Ecology

## Abstract

The Asian long-horned tick, *Haemaphysalis longicornis* Neumann, 1901, is the competent vector for severe fever with thrombocytopenia syndrome virus (SFTSV). *Haemaphysalis longicornis* originated mainly in eastern Asia and invaded many areas like Australia, New Zealand, and the Pacific islands, and was recently introduced to eastern parts of the USA. This species is characterized by high adaptability to a wide range of temperatures and can reproduce parthenogenically under stressful conditions. Migratory birds are important hosts of *H. longicornis* and are thought to be responsible for its unexpected invasion and introduction into new areas worldwide. This study predicted the historical (near current) global environmental suitability and the possible shifts in environmental suitability for *H. longicornis* under the ongoing climate change between 2021 and 2100. The results demonstrated that Europe is at potential of high environmental suitability for *H. longicornis* invasion although this species has not been recorded in any regions of Europe yet. Our model also anticipated the environmental suitability for *H. longicornis* in eastern parts of the USA, although the recently recorded occurrences there were not used in the model calibration. Climate change is thought to affect and increase the range of suitable environments for *H. longicornis.* The different maps introduced in this study may help improve understanding of the global environmental suitability for this invasive disease vector and predict the areas at high environmental suitability for possible invasion to prioritize the control programs and enhance quarantine procedures in these areas.

**Supplementary Information:**

The online version contains supplementary material available at 10.1038/s41598-025-86205-6.

## Introduction

The Asian long-horned tick *Haemaphysalis longicornis* Neumann, 1901, is an economically important livestock pest that threatens the health of humans, and wild, and domestic animals^1^. This species is considered a three-host tick that infested livestock hosts such as goats, sheep, cattle, and horses; wildlife hosts including deer, rats, mice, hedgehogs, and other birds; domestic pets (cats and dogs) as well as humans^1–4^. The original range of *H. longicornis* is in eastern Asia and was introduced into Australia, New Zealand, Pacific Islands^5,6^. Recently, the invasive *H. longicornis* was reported from New Jersey in 2017 for the first time from sheep^7,]^ then spread rapidly across 15 states and became established in the east of the United States^8,9^. *Haemaphysalis longicornis* exhibits both sexual and parthenogenetic reproductive strategies in nature^10^, this unusual biological characteristic and its high adaptability allow a single female tick to create a new population, especially at the edge of its current distribution, reinforcing its invasion ability^6^. The high adaptability and invasive ability may result in extensive geographical and host ranges of *H. longicornis* to new habitats like the recently documented invasion in the USA^11^. Moreover, Migratory birds might play a key role in species dispersal beating any geographical barriers^12^, meaning that more countries worldwide are threatened by unexpected invasion by *H. longicornis* and its associated pathogens.

*Haemaphysalis longicornis* is known as a competent vector for severe fever with thrombocytopenia syndrome virus (SFTSV) in China, South Korea, and Japan closely related to Heartland virus in the United States^13–15^. Other pathogens including *Anaplasma* spp., *Rickettsia* spp., *Babesia* spp., *Theileria* spp., and *Borrelia* spp. were detected in this species without any epidemiological significance^15–18^. In Australia and New Zealand, this tick species can also transmit *Theileria* orientalis Ikeda^1,19^. Thus, identifying the ecological niche of *H. longicornis* and the factors limiting its global distribution potential has become a global health concern and a global priority^6^.

Climate change, urbanization, international trade of wild and domestic animals, and human behaviors have all been suggested to have contributed to the rapid worldwide spread of vector-borne diseases^20,21^. The global distributional potential of *H. longicornis* is increasing daily due to several factors such as climate change, global livestock trades, birds’ migration, and urbanization^22^. Climatic variables are the primary factors limiting the global distribution of tick species^23,24^. Temperature and desiccation affect the reproduction, activity, and development of *H. longicornis*^25^. Thus, changes in climatic suitability may drive tick species to invade and establish populations in new areas worldwide. The global threat of *H. longicornis* invasion originates from its great level of tolerance to a variety of climatic conditions, multi-host preferences, asexual reproductive ability, and relationship with migratory birds^6–8^. In addition to these biological characteristics, changes in climatic conditions may influence vector invasion potential, meaning that more areas worldwide may be at risk of *H. longicornis* invasion. *Haemaphysalis longicornis* has been established in eastern parts of the United States, which reflects the rapid expansion of this tick species^26^. Such rapid invasion of potential medical and veterinary disease vectors worldwide is dangerous to both human and animal health. However, the effect of climate change on the potential global distribution of *H. longicornis* remains unclear.

Here, we used the most recent climatic and occurrence data to predict the historical (near-current) global environmental suitability for *H. longicornis* and regions of highly suitable environments for possible invasion by this invasive SFTSV vector. The present study tested the possibility of predicting the recent introduction of *H. longicornis* across the eastern parts of the USA using species occurrence data from southeast Asia and Oceania. This study is the first to highlight the possible influence of ongoing climate change on the global environmental suitability for *H. longicornis* between 2021 and 2100. The present research introduces different environmental suitability maps for *H. longicornis* under both historical (near current) and future conditions to enhance our understanding of this invasive SFTSV vector distribution. The generated models and maps may help prioritize areas that require control programs and quarantine procedures to avoid invasion and limit the distribution of *H. longicornis* consequentially limiting its associated disease distribution.

## Materials and methods

### Occurrence records

Primary occurrence records for *H. longicornis* were obtained from the Global Biodiversity Information Facility (GBIF; www.gbif.org; ~64 occurrence records), VectorMap (https://vectormap.si.edu; ~1123 occurrence records), the Atlas of Living Australia (www.ala.org.au; ~17 occurrence records), Inaturalist (www.inaturalist.org; ~27 occurrence records), and the previous scientific literature^6,27^ (~ 986 occurrence records). The dataset with a total of 2217 records was subjected to several data-cleaning steps to decrease possible biases in the estimation of the ecological niche models (ENMs). We discarded all records with unknown geographic coordinates and eliminated all duplicate records from the final dataset. The remaining records were filtered using a spatial rarefaction function in the SDM package in R, applying a distance-based filtering technique. This approach removed all duplicate records within each 2.5’ cell (~ 5 km), ensuring spatial independence of the data points^28,29^. The final data set (approximately 828 occurrence records) was randomly divided into two equal portions: 50% of the records for model calibration and 50% for evaluating the resulting model. The data random division was conducted using custom R script. The final data set used in the model calibration and evaluation is available at https://figshare.com/s/0e1292d38559dfa174b6.

### Climatic data

The historical (near current) and future climatic data were obtained from WorldClim version 2.1 (www.worldclim.org), with a spatial resolution of 2.5 arcminutes (≈ 5 km)^30^. Initially, the bioclimatic variables of historical conditions included 19 variables based on the monthly records of precipitation and temperature from 1970 to 2000. Bioclimatic variable numbers 8, 9, 18, and 19 were excluded from the analysis because spatially known artifacts were detected in these variables^31^. In response to climate change under different scenarios and periods, we obtained a parallel dataset for 8 diverse Global Climatic Models (GCMs), with the four expected Shared Socio-Economic Pathways (SSPs) representing the bioclimatic variables over four time periods: 2021–2040, 2041–2060, 2061–2080, and 2081–2100. The Shared Socio-Economic Pathways (SSPs) are a newly developed framework and are considered the extended range of Representative Concentration Pathways (RCPs) in the older version 1.4 ^32^. We used 8 GCMs (S. File 1) for each SSP in each period, with a total of 128 combinations (i.e., 8 GCMs × 4 SSPs × 4 time periods). Principal component analysis (PCA) was applied to the remaining 15 variables of the historical data to eliminate the excessive intercorrelations^33^. The PCA calculation and transfer code were written in R software version 3.2.0 to calculate the principal component of historical climatic variables and transfer the resulting PCAs to the parallel future climatic dataset^34^. We used the transferred PCAs of eight GCMs available from the WorldClim repository to determine the potential environmental suitability for *H. longicornis* under changing climate. The median of all ENM results based on each GCM was used to represent the future environmental suitability at each SSP for the four time periods to reduce the uncertainty of a single climate model scenario^35^. Among the 15 principal components (PCs), the first 8 PCs were chosen for model calibration because they contributed to more than 99.9% of the variance.

### Ecological niche modeling

**Fig. 1:**
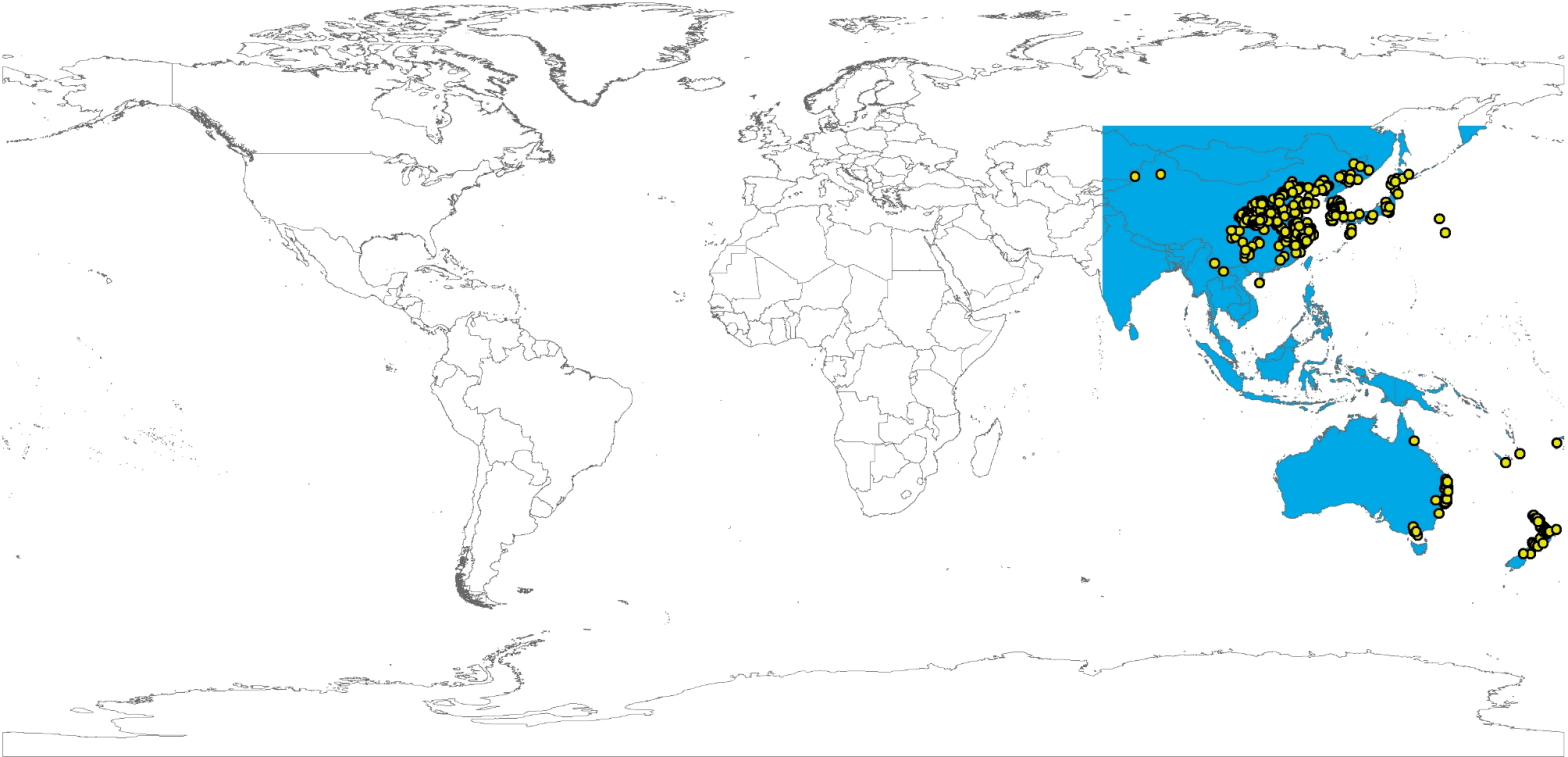
This figure depicts the accessible area (‘M’) for *Haemaphysalis longicornis*, shaded in blue, representing the geographic region considered in the modeling of the species’ historical distribution. The yellow solid points indicate the occurrence records used in the model, highlighting specific locations where *H. longicornis* has been observed. This map provides a visual overview of the study area and the data points that informed the historical distribution model, offering context for the spatial analysis of the species’ range.

ENMs for *H. longicornis* were estimated based on the maximum entropy algorithm employed in MaxEnt (version 3.3.3; http://www.cs.princeton.edu/wschapire/maxent/)^36^. We estimated an accessible area “M”^37^ that included Southeast Asia, and Oceania for this species (Fig. 1). The occurrence records of model calibration within this accessible area were only used in the model calibration, as MaxEnt ignored the occurrence records outside the identified accessible area. The ignored records were included in the data set used later in the model evaluation. In MaxEnt, the bootstrap function was used to create 10 replicates for each analysis with a random seed. We also deactivated the clamping and extrapolation options to avoid prediction in non-analogous environments^38^. For historical conditions, the median of all replicates was chosen to represent and visualize the environmental suitability for *H. longicornis* globally. Simultaneously, we calculated the median across all medians for each GCM to recapitulate the model results for future conditions. Our models were thresholded based on the assumption that up to 5% of the occurrence data may have been mistakenly recorded and discarded (E = 5%)^39^. We used the range (maximum-minimum) across the 10 model replicates to estimate the uncertainty estimates of the species ecological niche model under historical conditions^40^. On the other hand, the uncertainty estimates in the future models were determined as the range for each SSP’s GCM combinations (i.e., range across the 8 GCM resulted models, in each SSP)^33^.

### Model evaluation

The performance of the niche model for *H. longicornis* was evaluated using three approaches: (1) Partial ROC (pROC) statistics using the partial ROC function available in the Niche Toolbox platform^41^. Based on 1000 bootstrapped iterations, the ASCII format median layer obtained from MaxEnt and 50% of the occurrence records (i.e., testing records) were used to detect pROC values. (2) Using the occurrence records of the United States and the other occurrences that were filtered out during the early stages of data cleaning and were not used in the model calibration, a one-tailed cumulative binomial probability test was conducted to estimate the likelihood of obtaining the observed level of accurate predictions. (3) The extrapolation areas were detected using ExDet. tool software V1.1 to identify type I novelty (analogous and non-analogous environments) by comparing the 8 PCs of accessible “M” areas against the 8 PCs of global climatic data^42^. Areas of possible extrapolation across the world may result from projecting the calibrated models within the identified accessible area “M” for *H. longicornis* to the whole world.

To ensure transparency and reproducibility of our species distribution modeling (SDM) process, we have adhered to the guidelines set forth by the Overview, Data, Model, Assessment, and Prediction (ODMAP) protocol as outlined by Zurell and colleagues^43^ in the referenced publication. All relevant details concerning the modeling process, including data sources, model selection, and evaluation criteria, have been documented comprehensively. This documentation is provided as (S. File 2), where the completed ODMAP table is available, ensuring that our methods align with the recommended standards for reporting SDMs.

## Results

**Fig. 2:**
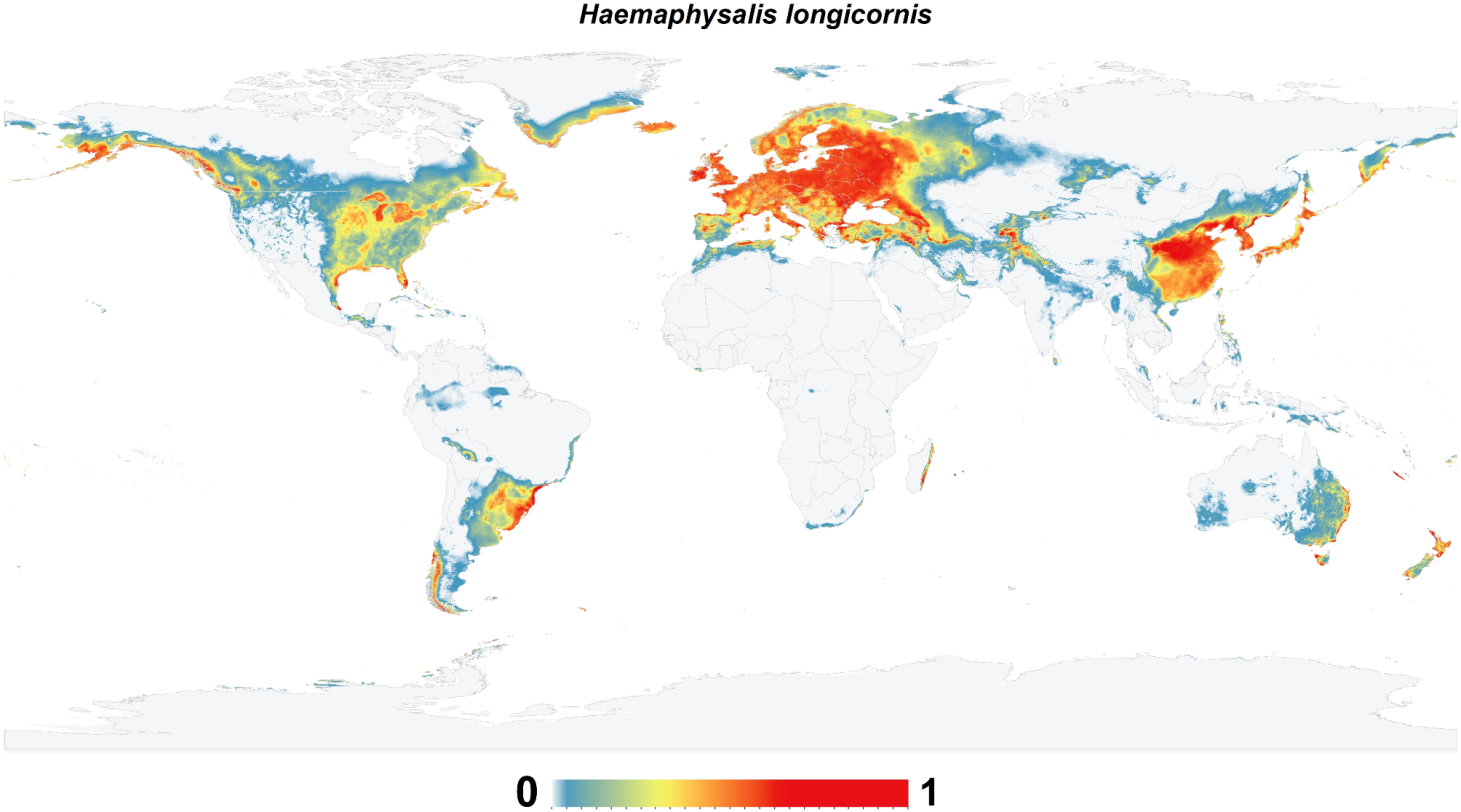
Probability map depicting the potential distribution of the Asian longhorned tick (*Haemaphysalis longicornis*) under historical climatic conditions. This map illustrates areas with suitable environmental conditions for the tick’s survival and proliferation, based on climatic factors. Regions highlighted indicate where the tick is most likely to establish and thrive, given historical climate patterns. The color gradient, ranging from light blue to dark orange, represents the gradient in probability from low to high. This map is instrumental in understanding the tick’s historical potential distribution, aiding in retrospective analyses and informing targeted surveillance and control efforts.

The historical global environmental suitability for *H. longicornis* was predicted to occur in China, Japan, North, and South Korea, northern areas of Vietnam, west, southwest, and southeast Russia, several Oceanian countries (including Fiji, New Caledonia, eastern areas of Australia, New Zealand and Vanuatu), southern parts of Papua New Guinea, Malaysia (particularly in Kuala Terengganu on the coast of the South China Sea), southern Sri Lanka, north India, Pakistan, Afghanistan, Uzbekistan, limited narrow zones in southern Kazakhstan, Kyrgyzstan, southwest Turkmenistan, north of Iran, and more narrow zones in south Iran, north of Iraq, Georgia, Azerbaijan, Armenia, Turkey, Cyprus, and northwest Syria on the coast of Mediterranean Sea (Fig. 2).

Interestingly, environments of the entire European continent are expected to be appropriate for the existence of this species. In Africa, *H. longicornis* showed potential environmental suitability patterns across North Africa near the northern coast of Tunisia, Morocco, and Algeria, and the eastern coastal areas of Madagascar. In North America, the environmental suitability for *H. longicornis* was observed in broad areas along the coastal states, eastern and central parts of the USA, southern Canada, and a small, constrained area along the coastline of the Gulf of Mexico. In South America, *H. longicornis* is anticipated to occur in Argentina, Uruguay, Chile, Paraguay, and Brazil, and in limited narrow zones in Peru and Bolivia (Fig. 2).

**Fig. 3:**
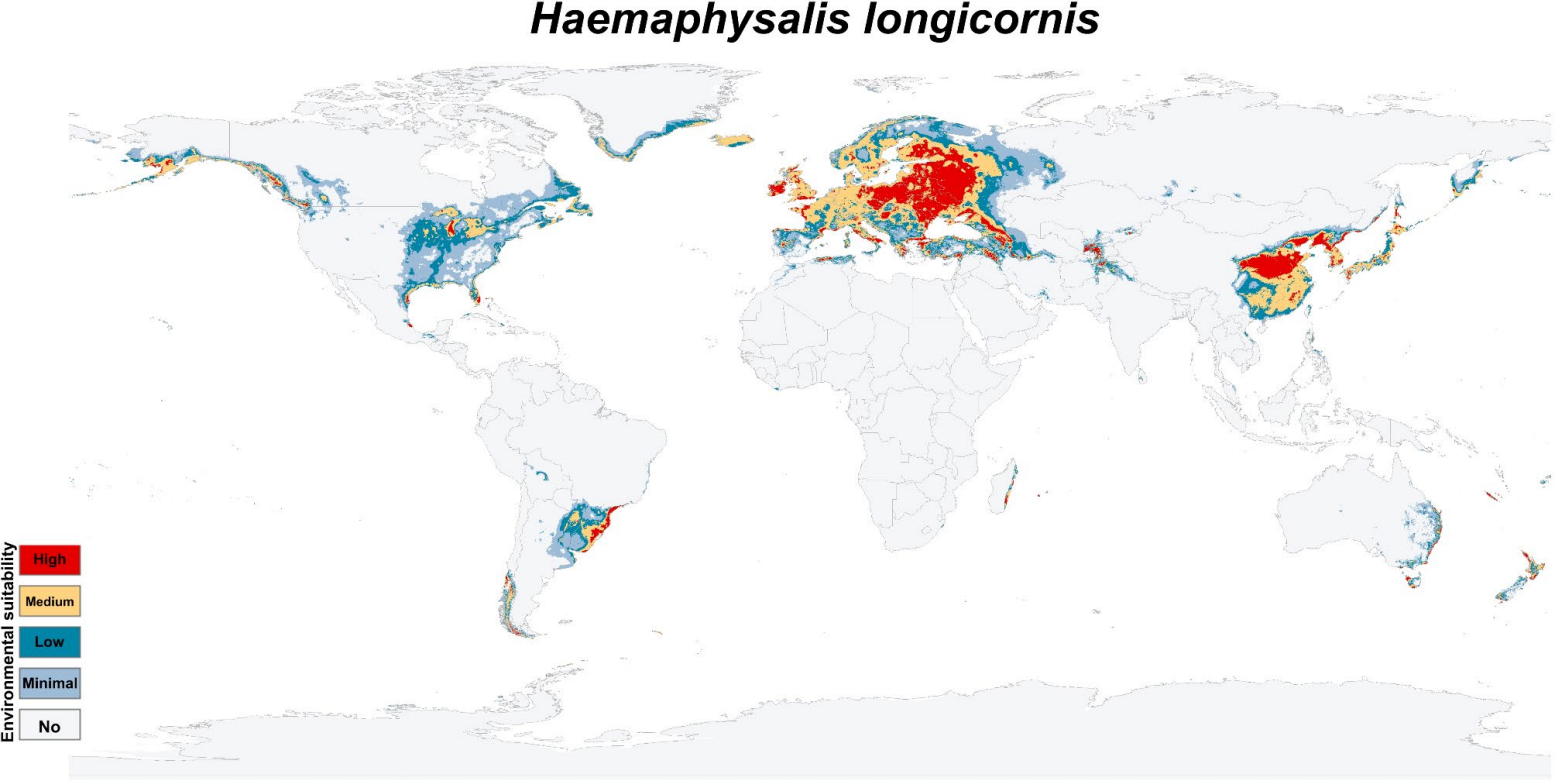
The classified environmental suitability map for the Asian longhorned tick (*Haemaphysalis longicornis*) under historical climate conditions. The map categorizes environmental suitability based on historical climatic factors, with a color-coded system: white denotes areas unsuitable for the tick’s survival, light grey indicates minimal suitability, blue represents low suitability, pale yellow shows medium suitability, and red marks regions of high environmental suitability. This classification offers valuable insights into the historical potential distribution of *Haemaphysalis longicornis*, serving as a baseline to understand how past environmental conditions have influenced the tick’s geographic range.

ENMs for *H. longicornis* yielded predictions under historical climatic conditions that predict areas with potentially high environmental suitability globally. Environmentally suitable habitats with a high potential for presence were primarily found in central and eastern Europe, eastern Asia, and western Russia, and limited narrow zones in southern and western Europe, the Americas, New Zealand, and the eastern coastline of Australia (Fig. 3). Also, the future potential environmental suitability maps were anticipated for *H. longicornis* (S. File 3).

The *H. longicornis* model performed noticeably better than random values and provided an area under the curve ratios above the null predictions (*P* < 0.001). The minimum, maximum, and mean pROC values were 1.72, 1.85, and 1.79, respectively. Of the independent records from the United States and occurrences filtered in early data cleaning procedures, 96% were correctly predicted by the resulting model. This was statistically superior to random expectations (*P* < 0.001; S. File 4).

**Fig. 4:**
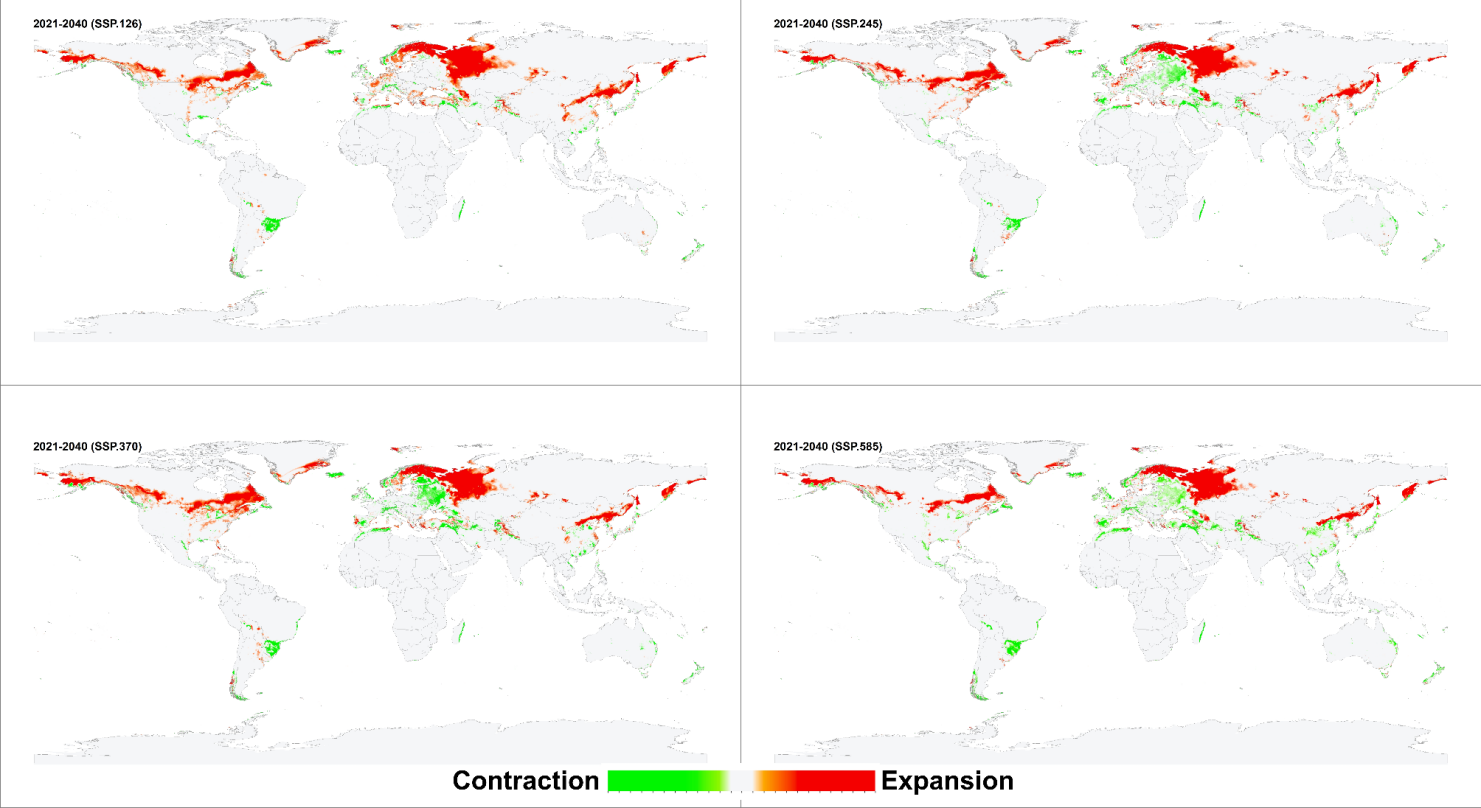
This figure presents four maps illustrating the projected impact of climate change on global environmental suitability for the Asian longhorned tick (*Haemaphysalis longicornis*) for the period 2021–2040, under four distinct Shared Socioeconomic Pathways (SSPs): SSP126, SSP245, SSP370, and SSP585. The maps use color gradients to depict changes in suitability: light to dark green shades indicate regions where the historical environmental suitability is anticipated to contract, while light to dark orange shades show areas where suitability is projected to expand, shift, or increase due to climate change. Light grey areas represent locations where the tick’s presence or absence remains stable across both historical and future climatic conditions. Together, these maps provide a comprehensive visual representation of how different climate change scenarios may influence the future distribution of *Haemaphysalis longicornis*.

**Fig. 5:**
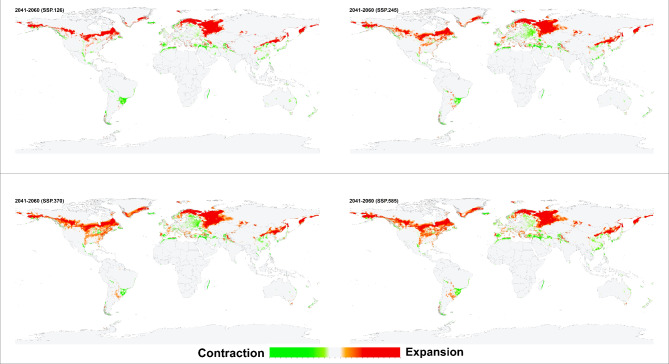
This figure presents four maps illustrating the projected impact of climate change on global environmental suitability for the Asian longhorned tick (*Haemaphysalis longicornis*) for the period 2041–2060, under four distinct Shared Socioeconomic Pathways (SSPs): SSP126, SSP245, SSP370, and SSP585. The maps use color gradients to depict changes in suitability: light to dark green shades indicate regions where the historical environmental suitability is anticipated to contract, while light to dark orange shades show areas where suitability is projected to expand, shift, or increase due to climate change. Light grey areas represent locations where the tick’s presence or absence remains stable across both historical and future climatic conditions. Together, these maps provide a comprehensive visual representation of how different climate change scenarios may influence the future distribution of *Haemaphysalis longicornis*.

**Fig. 6:**
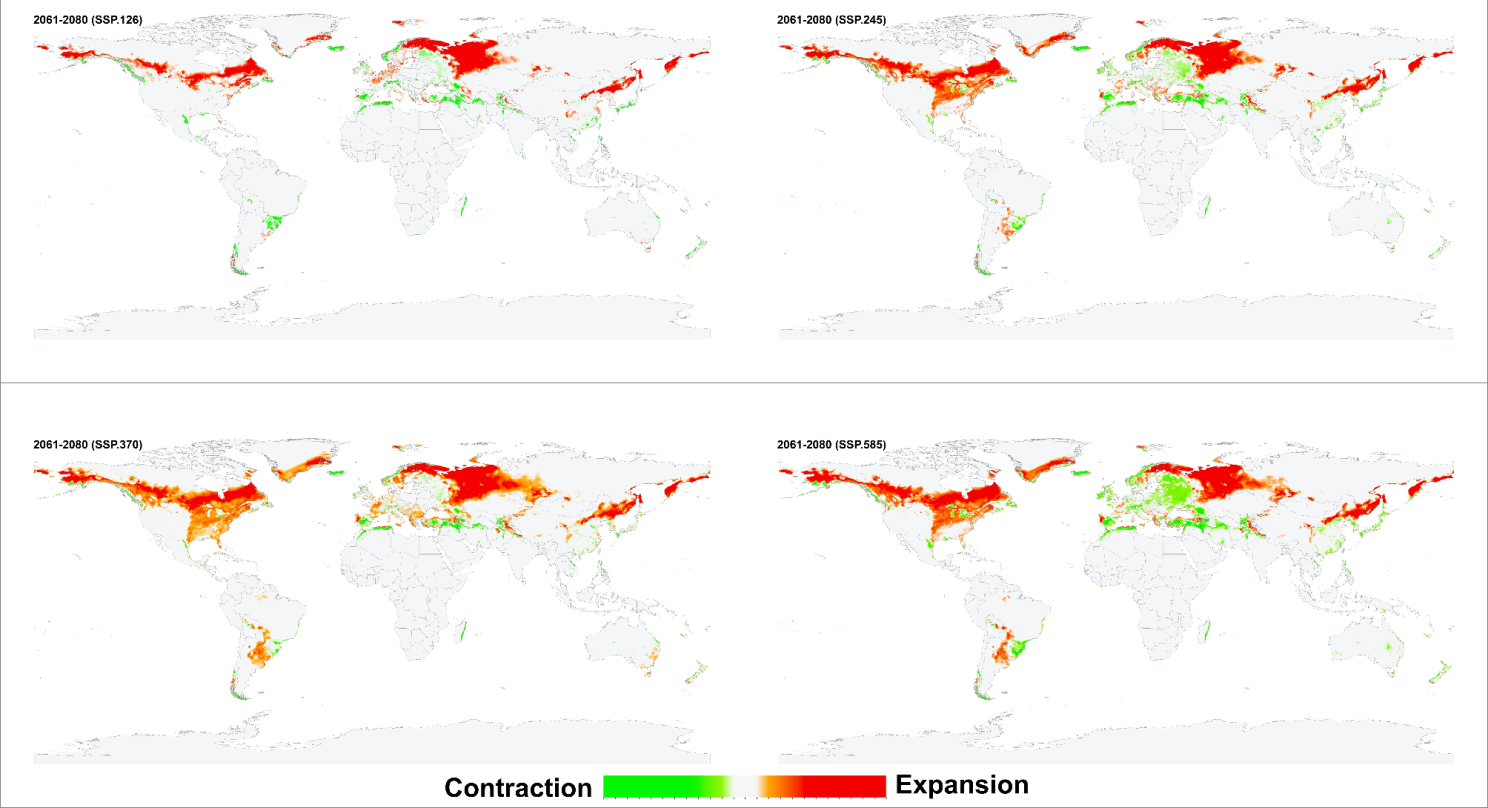
This figure presents four maps illustrating the projected impact of climate change on global environmental suitability for the Asian longhorned tick (*Haemaphysalis longicornis*) for the period 2061–2080, under four distinct Shared Socioeconomic Pathways (SSPs): SSP126, SSP245, SSP370, and SSP585. The maps use color gradients to depict changes in suitability: light to dark green shades indicate regions where the historical environmental suitability is anticipated to contract, while light to dark orange shades show areas where suitability is projected to expand, shift, or increase due to climate change. Light grey areas represent locations where the tick’s presence or absence remains stable across both historical and future climatic conditions. Together, these maps provide a comprehensive visual representation of how different climate change scenarios may influence the future distribution of *Haemaphysalis longicornis*.

**Fig. 7:**
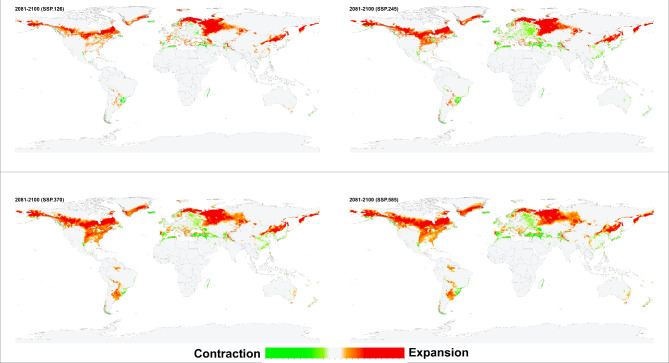
This figure presents four maps illustrating the projected impact of climate change on global environmental suitability for the Asian longhorned tick (*Haemaphysalis longicornis*) for the period 2081–2100, under four distinct Shared Socioeconomic Pathways (SSPs): SSP126, SSP245, SSP370, and SSP585. The maps use color gradients to depict changes in suitability: light to dark green shades indicate regions where the historical environmental suitability is anticipated to contract, while light to dark orange shades show areas where suitability is projected to expand, shift, or increase due to climate change. Light grey areas represent locations where the tick’s presence or absence remains stable across both historical and future climatic conditions. Together, these maps provide a comprehensive visual representation of how different climate change scenarios may influence the future distribution of *Haemaphysalis longicornis*.

The environmental suitability patterns of the historical and future conditions were similar when the resulting models were projected for future scenarios. However, an increase in the environmental suitability range was predicted in southern Canada, southern Greenland, western and southern Russia, northeastern China, and narrow zones in central Asia and South America. *Haemaphysalis longicornis* was also predicted to lose its suitable environments on the southern coastlines of Turkey and Spain and along the northern coasts of Tunisia, Morocco, and Algeria (Figs. 4, 5, 6, 7). Additionally, predictions revealed variances in environmental suitability ranges across several SSPs from 2021 to 2100. Overall, this species demonstrated the potential for expansion in its environmental suitability range in response to climate change, though some regions showed a likelihood of range contraction (Figs. 4, 5, 6, 7). Between the historical and future periods 2021–2041, the possible suitable environments of *H. longicornis* are predicted to increase by 11.39%, 11.90%, 10.37%, and 11.57%, and decrease by 1.74%, 2.04%, 2.23%, and 2.33% under SSP 126, SSP 245, SSP 370, and SSP 585, respectively (Fig. 4 & S. File 5). Between the historical and the future period 2041–2060, it is anticipated that the entire environmental suitability would rise by 16.05%, 18.26%, 21.35%, and 24.18%, and decrease by 2.44%, 2.39%, 2.42%, and 2.95% under SSP 126, SSP 245, SSP 370, and SSP 585, respectively (Fig. 5 & S. File 5). It is also predicted that the environmental suitability range would increase by 14.45%, 24.20%, 31.50%, and 34.66%, and decrease by 2.51, 2.88, 3.14, and 3.72% under SSP 126, SSP 245, SSP 370, and SSP 585, respectively between the historical and the future conditions 2061–2080 (Fig. 6 & S. File 5). Between the historical and the future conditions in 2081–2100, the environmentally suitable areas of *H. longicornis* are predicted to increase by 19.63%, 30.95%, 43.44%, and 49.67% and decrease by 2.15%, 2.88%, 3.77%, and 5.47% under SSP 126, SSP 245, SSP 370, and SSP 585, respectively (Fig. 7 & S. File 5).

*Haemaphysalis longicornis* environmental stability maps showed differences among the diverse SSPs from 2041 to 2100 (Figs. 4, 5, 6, 7, amp and S. File 5). In terms of past and future conditions, the regions with the highest environmental stability were the central and eastern parts of the USA, eastern parts of South America, eastern parts of Asia, and most of Europe.

**Fig. 8:**
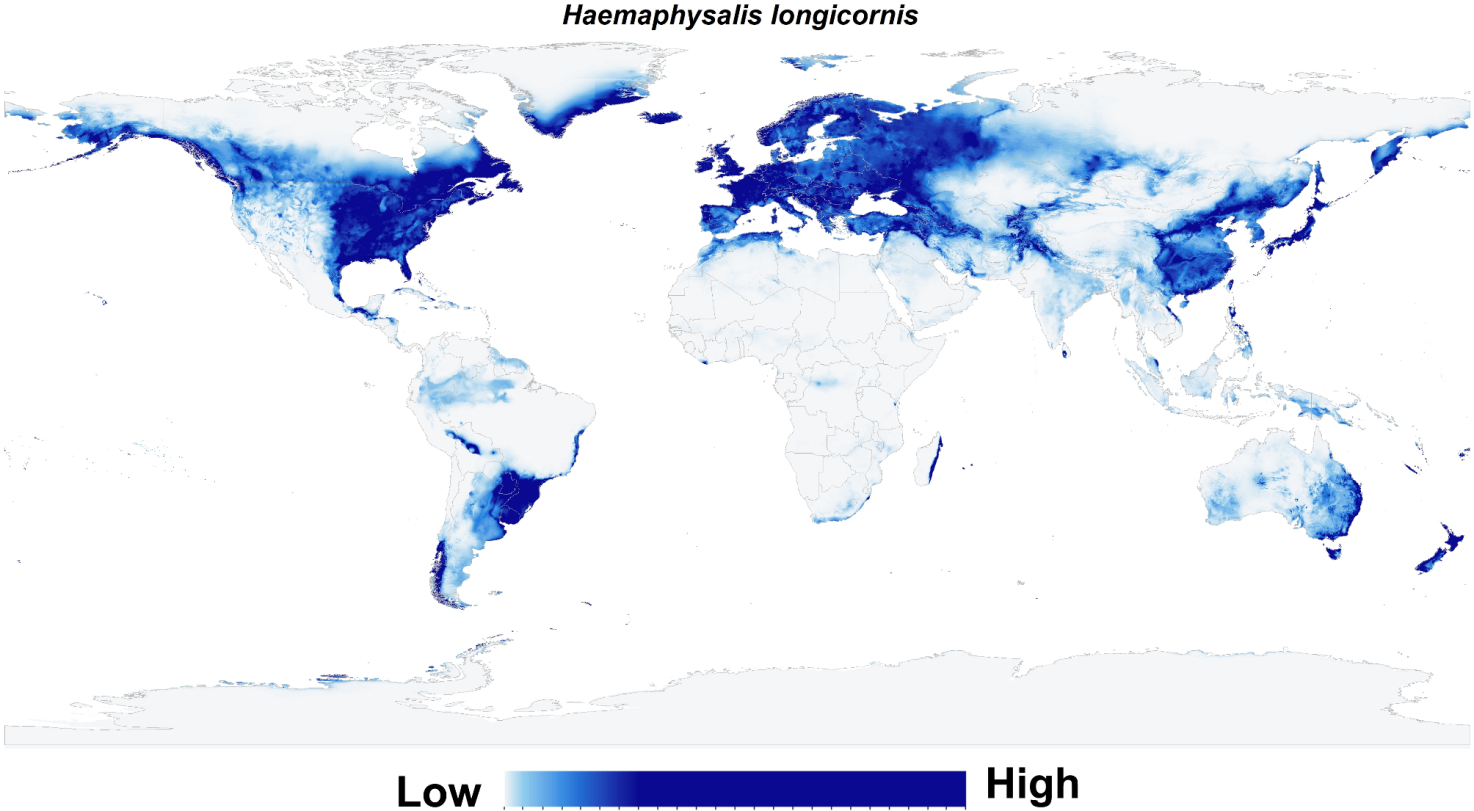
This map illustrates the uncertainty estimates in model predictions for *Haemaphysalis longicornis* distribution under historical climatic conditions. The uncertainty is calculated as the difference between the maximum and minimum prediction replicates. A gradient color scheme from light to dark blue is used to represent the range of uncertainty, with light blue indicating areas of low uncertainty and dark blue highlighting regions of high uncertainty. This visualization provides insight into the confidence levels of the model’s predictions, helping to identify areas where the results are most and least reliable.

**Fig. 9:**
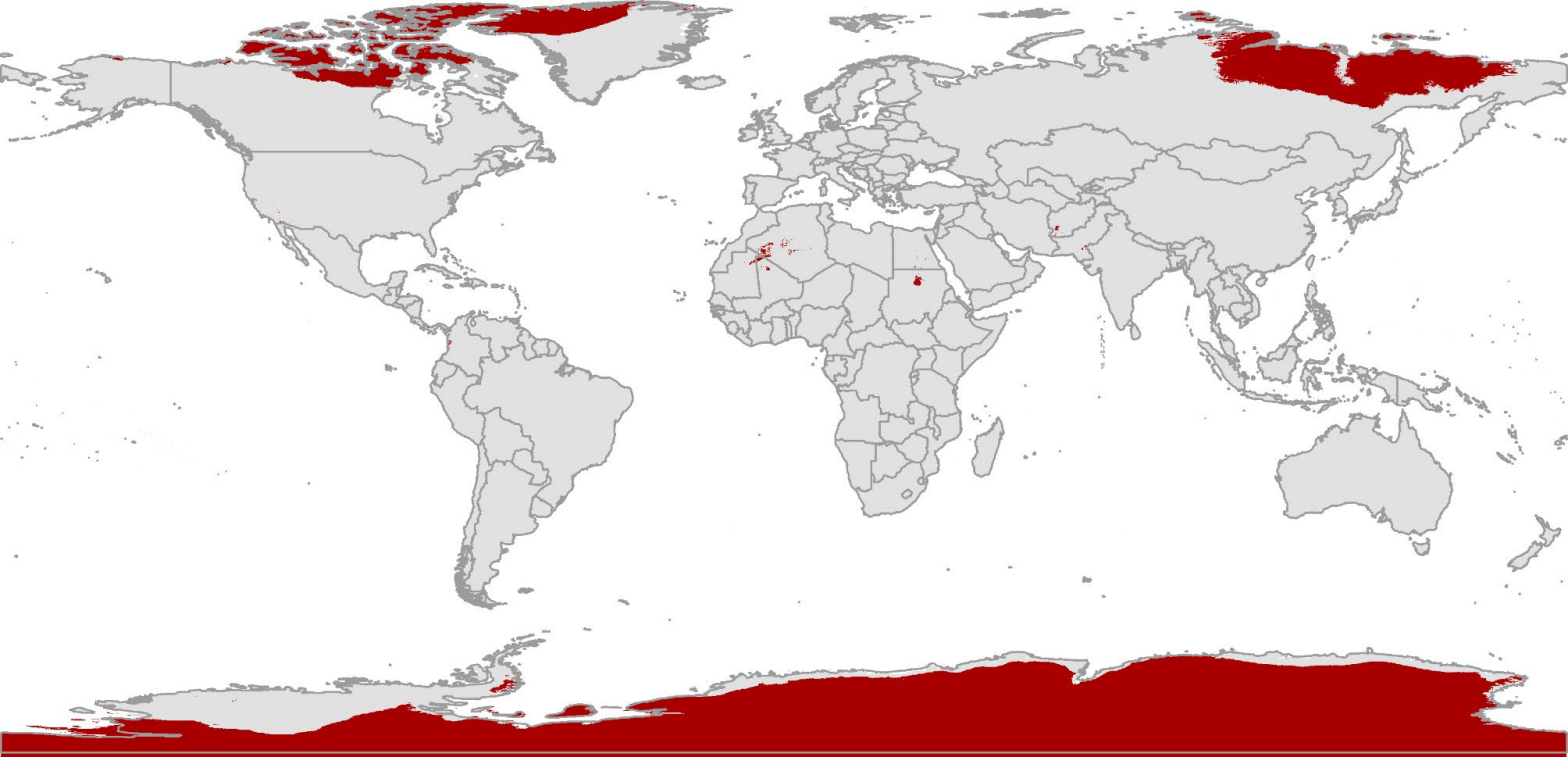
Extrapolation Detection (ExDet) output map for *Haemaphysalis longicornis* on a global scale. Regions highlighted in red indicate areas with ‘Type 1’ novelty, where environmental conditions differ significantly from those used in the model, suggesting caution in interpreting the model’s predictions in these areas. Gray regions represent areas with environmental conditions like those within the model’s training data, indicating a higher confidence in the model’s applicability. This map is crucial for identifying regions where model extrapolations may be less reliable due to novel environmental conditions.

The uncertainty estimate maps revealed regional variations in the uncertainty indices under historical settings. Uncertainty in model anticipations was determined in narrow zones in South America, central and eastern parts of Asia, the northern coasts of Tunisia, Morocco, and Algeria, and in many Western European countries. The model also displayed higher levels of uncertainty in Japan, New Zealand, and coastal areas of Madagascar, as well as in narrow zones in South America (Fig. 8). The degree of uncertainty in ENMs under future conditions varied between periods and SSPs. Eastern and Central Asia, southern Canada, South America, western Russia, and Europe were found to have the greatest variation between the different GCM predictions for *H. longicornis* among all the SSPs (S. File 6). The ExDet tool detected climatic novelty in our analysis under historical climatic conditions for *H. longicornis* in northern Greenland, northeast Russia, and northern Canada and showed pixels corresponding to climatic novelty in limited parts of North Africa (Fig. 9).

## Discussion

The present study predicted the spatial and temporal global environmental suitability for *H. longicornis* under diverse climatic conditions, with significant implications for public health and epidemiology in regions worldwide that may be at risk of actual occurrence or possible invasion by this species. This study is crucial for several reasons: (1) It provided comprehensive maps estimating the likelihood of *H. longicornis* establishing in various new and diverse continents, highlighting regions with high environmental suitability that may be vulnerable to potential invasion under the historical (near-current) climatic conditions (2) The study evaluated the potential for *H. longicornis* to spread, particularly highlighting regions that may become more vulnerable due to global warming, as projected by various climate change scenarios. (3) It produced a variety of maps that are essential for identifying priorities for mitigation and surveillance programs aimed at controlling the spread of *H. longicornis*.

The historical and probable future environmental suitability for *H. longicornis* were visualized through a range of maps, including probability maps (Fig. 2 & S. File 7), potential environmental suitability classified maps (Fig. 3 & S. File 3), and potential environmental stability maps (Figs. 4, 5, 6, 7, and S. File 5). These maps were further complemented by uncertainty estimates (Fig. 8 & S. File 6) in the resulting models, which were crucial in identifying areas where extrapolation might be necessary (Fig. 9). Together, these maps provide a comprehensive visualization of the global potential environmental suitability for this invasive species, helping to clarify both the historical and potential future environmental suitability for *H. longicornis*. This study underscored the potential global distribution of environmental suitability for *H. longicornis* under various climate change scenarios, indicating significant shifts in environmental suitability. The findings suggest that regions previously considered as low environmentally suitable, particularly in Europe and North America, may soon face new threats. This shift has profound implications for public health, as the spread of this vector could lead to the introduction and establishment of diseases such as severe fever with thrombocytopenia syndrome (SFTS) in these areas. The results highlight the urgent need for enhanced surveillance and proactive measures to mitigate the potential impacts of this invasive species.

Future research should focus on refining predictive models by incorporating additional environmental and anthropogenic factors, as well as exploring effective intervention strategies to control the spread of *H. longicornis* in vulnerable areas. These efforts are critical for improving our ability to anticipate and respond to the challenges posed by this invasive species.

Several studies have explored the potential distribution of *H. longicornis* at both local and global scales, particularly focusing on regions like the USA^3,6,11,27,44^. However, the present study extends this work by employing the most recent climatic and species occurrence data to model the global distribution of environmental suitability for *H. longicornis*, providing a comprehensive visualization of its historical and potential future distribution of suitable environments under various climate change scenarios. This study is unique in that it is the first to systematically examine shifts in environmental suitability for *H. longicornis* across different climate projections, revealing higher environmental suitability for potential invasion in Europe. Remarkably, this prediction comes despite the absence of recorded occurrences in Europe, a discrepancy that may be attributed to historically weak surveillance programs or stringent quarantine measures that have prevented detection.

Our modeling approach, which was calibrated using occurrence records from regions such as Asia, Australia, and New Zealand, successfully predicted environmental suitability for *H. longicornis* in the eastern USA—a region where the species has been confirmed through multiple recordings^3,7–9,26,45,46^. This successful prediction suggests a potential analogous invasion scenario could unfold in Europe, as well as in other high-environmentally suitable areas such as western and southern Canada, the eastern coasts of Madagascar, southern Chile, and the border regions between Argentina and Brazil. Even though *H. longicornis* has not yet been recorded in these regions, the environmental conditions indicated by our model show high environmental suitability, particularly along coastal margins, which is consistent with findings from previous research^3,11,27,44^. Our study underscores the significant threat *H. longicornis* poses to European countries, which should be urgently addressed in both surveillance and quarantine strategies to prevent a possible invasion. The high environmental suitability indicated in our historical models for Europe, despite the lack of occurrence records, highlights the need for proactive measures.

Our findings show considerable overlap with the predictions made by Raghavan and colleagues^11^, especially in southeastern USA. However, their uncertainty models identified high uncertainty in regions like the Pacific Northwest, Mexico, and Central America, areas where our model also indicated potential but with less confidence. Additionally, our results align with those of Namgyal and colleagues^44^, who suggested that Mexico is not a highly suitable environment for *H. longicornis*, and with Rochlin’s study^3^, which identified high-environmental suitability in North America’s coastal areas, a finding consistent with our model’s predictions.

However, our results diverge from those of Zhao and colleagues^6^, who predicted greater environmental suitability for *H. longicornis* along the western coast of the USA and in specific regions of western and southern Europe, including Turkey, Spain, the UK, Ireland, France, Albania, and Greece. In contrast, our model indicates that the entire European continent—including western, southern, northern, and eastern regions—exhibits high environmental suitability, suggesting a broader potential for invasion than previously anticipated.

Overall, this study not only confirms the findings of earlier research in many respects but also expands upon them by providing a more nuanced understanding of the global distribution and potential future spread of environmental suitability for *H. longicornis*. This highlights the urgent need for global cooperation in enhancing surveillance and implementing stringent measures to mitigate the risks associated with the spread of this invasive species.

The ecological niche models for *H. longicornis* were based and evaluated in a series of steps to ensure the most efficient model accuracy as follows: First, the ecological niches of *H. longicornis* were identified using the most recent climatic data and the most updated 2217 species occurrences records in comparison with only 249 occurrence points used by Zhao and colleagues^6^, with a series of data set cleaning to remove uncertain occurrences and avoid any biased occurrences. Second, the models were based on the principal components of the bioclimatic variables to avoid and reduce the dimensionality and multicollinearity between these variables, which would lead to the lowest possible model accuracy. Third, the accuracy of the resulting models was evaluated using different techniques to ensure their predictivity, and the anticipated areas, especially in the USA, were matched with the occurrence records of *H. longicornis* recently recorded there although these occurrences were not used in the model calibration. Fourth, uncertainty estimates in model anticipation were identified to complete the model prediction accuracy, and possible extrapolation in non-analogous areas through projection was identified to ensure that most of the predicted areas were analogous to the identified accessible areas of model calibration at the origin of the species.

Projecting the ecological niche model at the species’ origin to the whole world helps predict and identify suitable environments for possible invasion pathways across the world. The limitation of using host data in our model calibration does not affect the model accuracy; hence, the species is a multi-host species with high adaptability to different hosts. Bioclimatic variables are thought to limit and influence the distribution of species because they can adapt to and associate with many hosts. However, a study byZhao and colleagues^6^ suggested that the distribution of *H. longicornis* is not only determined by climatic factors but also by land cover type. The present predictions support the climatic variables in its principal component state were able to predict the global potential distribution of suitable environments for *H. longicornis*, especially in the predicted areas in the USA. Normalized Difference Vegetation Index (NDVI) variables were neglected in this study because of the lack of parallel future data on NDVI variables^29,40^. Additionally, climatic variables are better predictors of the potential distribution of tick vectors than vegetation-derived variables, and climate is the primary factor that directly affects tick distribution^23,24^.

Climate change has undoubtedly become a global threat that requires attention because its consequences have become more notable everywhere. Climate change is believed to influence the distribution of several disease vectors and their transmission in new areas worldwide^33^. Here, we visualized for the first time the possible influence of ongoing climate changes on the global potential distribution of suitable environments for *H. longicornis* based on different expected scenarios and socioeconomic pathways. The predicted future models revealed the possible influence of climate change on suitable environments for *H. longicornis*, consequentially influencing its distribution and establishment. Climate change is predicted to extend the suitable environments for *H. longicornis* which means that new areas will be at high environmental suitability for its distribution, consequently introducing transmitted pathogens to these new areas. Not only will new areas be highly suitable environments, but the probability of environmental suitability distribution in historically anticipated areas will also increase. Climate change does not mean environmental range expansion alone; the currently anticipated suitable environments may become unsuitable under a changing climate. Climate change will undoubtedly influence the distribution of environmental suitability for *H. longicornis* because the activity, reproduction, and development of this invasive vector are mainly affected by temperature and desiccation during the environmental phases of its life cycle^25^.

An important consideration in the interpretation of our model output is the observed high uncertainty in areas where environmental suitability for *H. longicornis* is predicted to be high. This overlap between high uncertainty and high environmental suitability suggests that while these regions may be environmentally favorable for the species, the predictions should be approached with caution. The high uncertainty could result from several factors, including complex environmental conditions, insufficient data, or inherent variability in the ecological factors driving the model. This limitation emphasizes the need for further data collection and refinement of the model to enhance the reliability of predictions in these areas. Consequently, while the model provides valuable insights into potential environments, the associated uncertainty limits the confidence in these predictions and highlights the necessity for complementary validation methods or additional surveillance in these high-environmental suitability regions.

In summary, our study clarified, confirmed, and improved the historical distributional potential of suitable environments for *H. longicornis*. Additionally, it placed new regions at high potential environmental suitability for vector invasion under both historical and future climatic conditions. We can detect and observe the prospective distribution of suitable environments for this significant invasive vector using ecological niche modeling approaches, regardless of the environmental circumstances of the present or the future. Several diseases may originate or re-emerge in new and historic places because of *H. longicornis* invasions. The invasion of this species has significantly increased the risk of deadly diseases in several new areas, particularly in Europe, where environmental conditions are becoming increasingly suitable for its spread, although *H. longicornis* has not yet been recorded there.

## Conclusion

The present work provides critical insights into the potential future environmental suitability for *H. longicornis* in a changing climate. The predicted expansion of suitable environments for this species highlights the growing risk of vector-borne diseases in new regions. These results underscore the necessity for global cooperation in surveillance and control efforts to manage the spread of invasive vectors. As climate change continues to reshape ecosystems, our findings call for a reevaluation of current public health strategies to address emerging risks associated with vector-borne diseases. Continued research is essential to anticipate and mitigate these risks, ensuring preparedness in regions that may soon face new public health challenges.

## Electronic supplementary material

Below is the link to the electronic supplementary material.

Supplementary Material 1

Supplementary Material 2

Supplementary Material 3

Supplementary Material 4

Supplementary Material 5

Supplementary Material 6

Supplementary Material 7

## Data Availability

The datasets used and/or analyzed during the current study are available from the corresponding author upon reasonable request.
