# Supplementary material for: Climate change influences on the potential geographic distribution of the invasive Asian longhorned tick, Haemaphysalis longicornis
Source: Sci Rep. 2025 Jan 17;15:2266. doi: 10.1038/s41598-025-86205-6 (PMC11748616; doi:10.1038/s41598-025-86205-6)
Supplement: Supplementary file 1 — Supplementary Material 1 [file 41598_2025_86205_MOESM1_ESM.pdf]

S. File 1: List of the 8 global climatic models (GCMs) used in the present study to predict the global potential distribution of *Haemaphysalis longicornis* under changing climate

GCM	Modeling Center or Group
BCC-CSM2-MR	Beijing Climate Center , China.
CNRM-CM6-1	National Centre for Meteorological Research , France.
CNRM-ESM2-1	National Centre for Meteorological Research , France.
CanESM5	The Canadian Earth System Model , Canada.
IPSL-CM6A-LR	Institute Pierre-Simon Laplace , France.
MIROC-ES2L	Atmosphere and Ocean Research Institute, National Institute for Environmental Studies & Japan Agency for Marine-Earth Science and Technology, Japan.
MIROC6	Atmosphere and Ocean Research Institute, National Institute for Environmental Studies & Japan Agency for Marine-Earth Science and Technology, Japan.
MRI-ESM2-0	The Meteorological Research Institute, Japan.
