# Supplementary material for: Climate change influences on the potential geographic distribution of the invasive Asian longhorned tick, Haemaphysalis longicornis
Source: Sci Rep. 2025 Jan 17;15:2266. doi: 10.1038/s41598-025-86205-6 (PMC11748616; doi:10.1038/s41598-025-86205-6)
Supplement: Supplementary file 2 — Supplementary Material 2 [file 41598_2025_86205_MOESM2_ESM.pdf]

Mapping the global potential distributions of *Haemaphysalis longicornis* Neumann, 1901 under changing climate

– ODMAP Protocol –

Mohammed Okely, Ze Chen, Eslam Adly, Mahmoud Kamal

2024-08-11

Overview

Authorship

Contact : mohamedokely@sci.asu.edu.eg

Model objective

Model objective: Mapping and interpolation

Target output: Maps of potential global distribution under historical and future climate scenarios

Focal Taxon

Focal Taxon: *Haemaphysalis longicornis*

Location

Location: Global scale

Scale of Analysis

Spatial extent: -180, 180, -56, 72 (xmin, xmax, ymin, ymax)

Spatial resolution: 2.5 arcminutes (~5 km)

Temporal extent: 1970–2100

Temporal resolution: Four future periods (2021–2040, 2041–2060, 2061–2080, 2081–2100)

Boundary: rectangle

Biodiversity data

Observation type: field survey, citizen science, standardised monitoring data

Response data type: point occurrence, presence-only

Predictors

Predictor types: climatic

Hypotheses

Hypotheses: Climatic factors, particularly temperature and precipitation, are the primary drivers of the distribution of *Haemaphysalis longicornis*.

Assumptions

Model assumptions: The species distribution is primarily limited by climatic conditions, and the data used are representative of the actual distribution.

Algorithms

Modelling techniques: Maxent

Model complexity: The model complexity was carefully balanced to ensure accurate predictions while avoiding overfitting:

Principal Component Analysis (PCA): PCA was applied to reduce the number of predictor variables, minimizing multicollinearity and simplifying the model without sacrificing important environmental information.

MaxEnt Regularization: Default regularization settings were used in MaxEnt to control model complexity, preventing the model from fitting noise in the data and enhancing generalization across different regions.

Use of Median Predictions: Averaging across multiple Global Climate Models (GCMs) and Shared Socioeconomic Pathways (SSPs) further moderated complexity, ensuring that the final predictions were robust and not overly influenced by individual models.

These steps ensured that the model was complex enough to capture the necessary environmental relationships while remaining generalizable and reliable for global predictions.

Model averaging: Ensemble modeling and model averaging were employed to enhance the robustness and reliability of the predictions:

Model Averaging:

Predictions from multiple Global Climate Models (GCMs) and Shared Socioeconomic Pathways (SSPs) were averaged to produce a consensus model for each future scenario. The median of these predictions was used as the final output, reducing the influence of outliers and providing a more stable and reliable prediction of *Haemaphysalis longicornis* distribution.

Ensemble Modelling:

The ensemble approach integrated results from eight different GCMs across four SSPs, each representing different climate change trajectories. This method allowed the model to account for variability and uncertainty in future climate conditions, ensuring that the predictions were not overly dependent on any single model or scenario. The use of model averaging and ensemble modeling provided a more comprehensive and robust prediction, capturing a wider range of potential outcomes and reducing uncertainty in the projected distributions.

Workflow

Model workflow: Data Collection → Data Cleaning → Model Calibration → Model Validation → Prediction of Distribution → Uncertainty Assessment

Software

Software: MaxEnt 3.3.3

Code availability: codes and data available upon request from the corresponding author

Data availability: GBIF, VectorMap, Atlas of Living Australia, iNaturalist, and scientific literature sources

Data

Biodiversity data

Taxon names: *Haemaphysalis longicornis*

Taxonomic reference system: The taxonomic reference system used in this study for *Haemaphysalis longicornis* followed the most current and widely accepted classifications as documented in the following sources:

Global Biodiversity Information Facility (GBIF): Taxonomic names and classifications were cross-referenced with the GBIF database to ensure accuracy and consistency.

Integrated Taxonomic Information System (ITIS): Taxonomic information was also verified against ITIS to ensure alignment with global standards.

Scientific Literature: The species identification and classification were consistent with descriptions and classifications provided in peer-reviewed entomological and parasitological literature.

Ecological level: species

Data sources: GBIF, VectorMap, Atlas of Living Australia, iNaturalist, scientific literature

Spatial Design: Data were spatially thinned to one occurrence per 5 km grid cell, ensuring uniform distribution and minimizing spatial autocorrelation.

Cross-Sectional Design: Data include historical records (1970-2000) and projections for future periods (2021-2100), capturing both past and future distributions under varying climate scenarios.

Sample size: Approximately 828 unique occurrence records after data cleaning

Cleaning: Duplicate records removed; spatial filtering applied using SDM Toolbox 2.4

Absence data: Not applicable (presence-only modeling)

Stratified Sampling: The occurrence data for *Haemaphysalis longicornis* were collected from various sources, covering a wide geographic area. The data were spatially filtered using a stratified approach, ensuring that records were evenly distributed across different environmental strata and reducing spatial bias.

Cross-Sectional Design: The sampling spanned a broad temporal range, utilizing historical data from 1970 to 2000 and projecting into the future (2021-2100). This design captured both current and potential future distributions under different climate scenarios. **Nestedness:**

Nested Sampling: Data were nested by geographic regions (e.g., continents, countries), allowing for the assessment of both broad-scale global patterns and finer-scale regional variations in the species' distribution.

Errors and biases: Potential sampling bias in the occurrence data

Data partitioning

Training data: 50% of the occurrence records

Validation data: 50% of the occurrence records

Predictor variables

Predictor variables: WorldClim v2.1

Data sources: https://www.worldclim.org/

Spatial extent: Global; -180, 180, -56, 72 (xmin, xmax, ymin, ymax)

Spatial resolution: 2.5 arcminutes

Coordinate reference system: CRS Name: WGS 84 (World Geodetic System 1984)

Temporal extent: The raw occurrence data for *Haemaphysalis longicornis* used in this study cover the following temporal extent:

Historical Data: 1970 to 2000 Future Projections: 2021 to 2100 (for climate scenario modeling)

Temporal resolution: Monthly

Data processing: Principal Component Analysis (PCA) applied to bioclimatic variables

Transfer data

Data sources: WorldClim v2.1 future projections (SSPs 126, 245, 370, 585)

Spatial extent: global; -180, 180, -56, 72 (xmin, xmax, ymin, ymax)

Spatial resolution: Same as for historical data

Temporal extent: Same as for historical data

Temporal resolution: Same as for historical data

Models and scenarios: BCC-CSM2-MR, CNRM-CM6-1, CNRM-ESM2-1, CanESM5, IPSL-CM6A-LR, MIROC-ES2L, MIROC6, and MRI-ESM2-0

Quantification of Novelty: Novel environmental conditions and combinations were quantified using the following methods:

Distance to Training Data:

The study used the Extrapolation Detection (ExDet) tool to quantify the novelty of environmental conditions when projecting the species distribution model (SDM) into new areas or future scenarios.

Type 1 Novelty: Identified regions where environmental variables fall outside the range of the training data, indicating truly novel conditions not represented in the original dataset.

Model

Variable pre-selection

Variable pre-selection: PCA applied to reduce multicollinearity among predictor variables

Multicollinearity

Multicollinearity: PCA applied to reduce multicollinearity among predictor variables

Model settings

<maxent>

Model settings (extrapolation): Clamping and Extrapolation were deactivated

Model estimates

Coefficients: pROC and binomial probability test.

Parameter uncertainty: uncertainty estimates were mapped and provided for both historical and climate change conditions

Variable importance: PCA was conducted and PCs with the highest contribution to the overall variance were chosen

Model selection - model averaging - ensembles

Model selection: The median of the model outputs across all GCMs used for final predictions

Model averaging: The median of the model outputs across all GCMs used for final predictions

Model ensembles: In this study, an ensemble modeling approach was employed to account for the variability and uncertainty inherent in species distribution modeling under different climate change scenarios. The ensemble method integrated predictions from multiple Global Climate Models (GCMs) and Shared Socioeconomic Pathways (SSPs) to produce more robust and reliable predictions of the potential distribution of *Haemaphysalis longicornis*.

Analysis and Correction of non-independence

Spatial autocorrelation: Spatial autocorrelation in residuals was addressed using a combination of data filtering techniques and model evaluation methods to ensure that the species distribution model (SDM) for *Haemaphysalis longicornis* provided unbiased and reliable predictions.

Spatial autocorrelation was managed by:

Spatial Filtering: Thinning occurrence records to one per 5 km grid cell using the spatial rarefaction function in the SDM package in R, reducing clustering and autocorrelation in the data.

Model Validation: Evaluating model performance using partial ROC and Moran's I statistic to detect and minimize spatial autocorrelation in residuals.

PCA on Predictors: Applying PCA to reduce multicollinearity and ensure predictors were not spatially correlated.

Threshold selection

Threshold selection: 5% omission threshold applied

Assessment

Performance statistics

Performance on training data: Area under the curve (AUC) values consistently above random predictions.

Performance on validation data: Independent records from the USA used for validation.

Plausibility check

Response shapes: map display

Prediction

Prediction output

Prediction unit: Probability of presence.

Post-processing: Clipping to geographic regions of interest.

Uncertainty quantification

Scenario uncertainty:

Uncertainty in Climate Models:

Multiple GCMs: The study utilized predictions from eight different Global Climate Models (GCMs) to capture a range of possible climate futures. This approach accounted for variability in climate model outputs, providing a more comprehensive assessment of uncertainty.

Ensemble Averaging: The median of predictions from all GCMs was used to reduce the impact of outliers and extreme values, resulting in a more stable and reliable prediction. Uncertainty in Scenarios:

Shared Socioeconomic Pathways (SSPs): Four SSPs (126, 245, 370, 585) were used to represent different potential futures based on varying levels of greenhouse gas emissions and socioeconomic development. This allowed the study to account for uncertainties in future emissions and land-use changes.

Scenario-Based Variability: The variability in predictions across different SSPs was quantified, providing insights into how different future scenarios could impact the distribution of *Haemaphysalis longicornis*.

Range of Outcomes: By integrating results from multiple scenarios and storylines (combinations of GCMs and SSPs), the study provided a range of potential outcomes, highlighting areas of agreement and divergence in the predictions.

Extrapolation Detection (ExDet): The ExDet tool was used to identify areas where novel environmental conditions might occur, flagging these as regions with higher uncertainty in the predictions. Overall, uncertainty was rigorously quantified by using multiple models, scenarios, and storylines, ensuring that the predictions account for a broad spectrum of possible future conditions.
