# Supplementary material for: Climate change influences on the potential geographic distribution of the invasive Asian longhorned tick, Haemaphysalis longicornis
Source: Sci Rep. 2025 Jan 17;15:2266. doi: 10.1038/s41598-025-86205-6 (PMC11748616; doi:10.1038/s41598-025-86205-6)

S. File 3: This file presents classified environmental suitability maps for the tick species *Haemaphysalis longicornis* under various climate change scenarios across four different time periods. The maps are based on four Shared Socioeconomic Pathways (SSPs), which include SSP.126, SSP.245, SSP.370, and SSP.585. Suitability is color-coded, with white indicating areas that are not environmentally suitable, light grey representing minimal suitability, blue for low suitability, pale yellow for medium suitability, and red highlighting regions of high environmental suitability. These classifications reflect the potential distribution shifts of *Haemaphysalis longicornis* in response to changing climate conditions, providing insight into areas that may become increasingly favorable for the species over time.

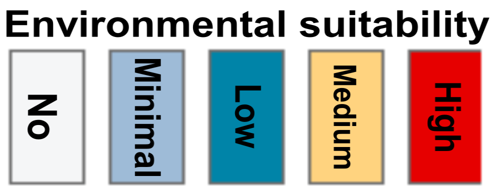

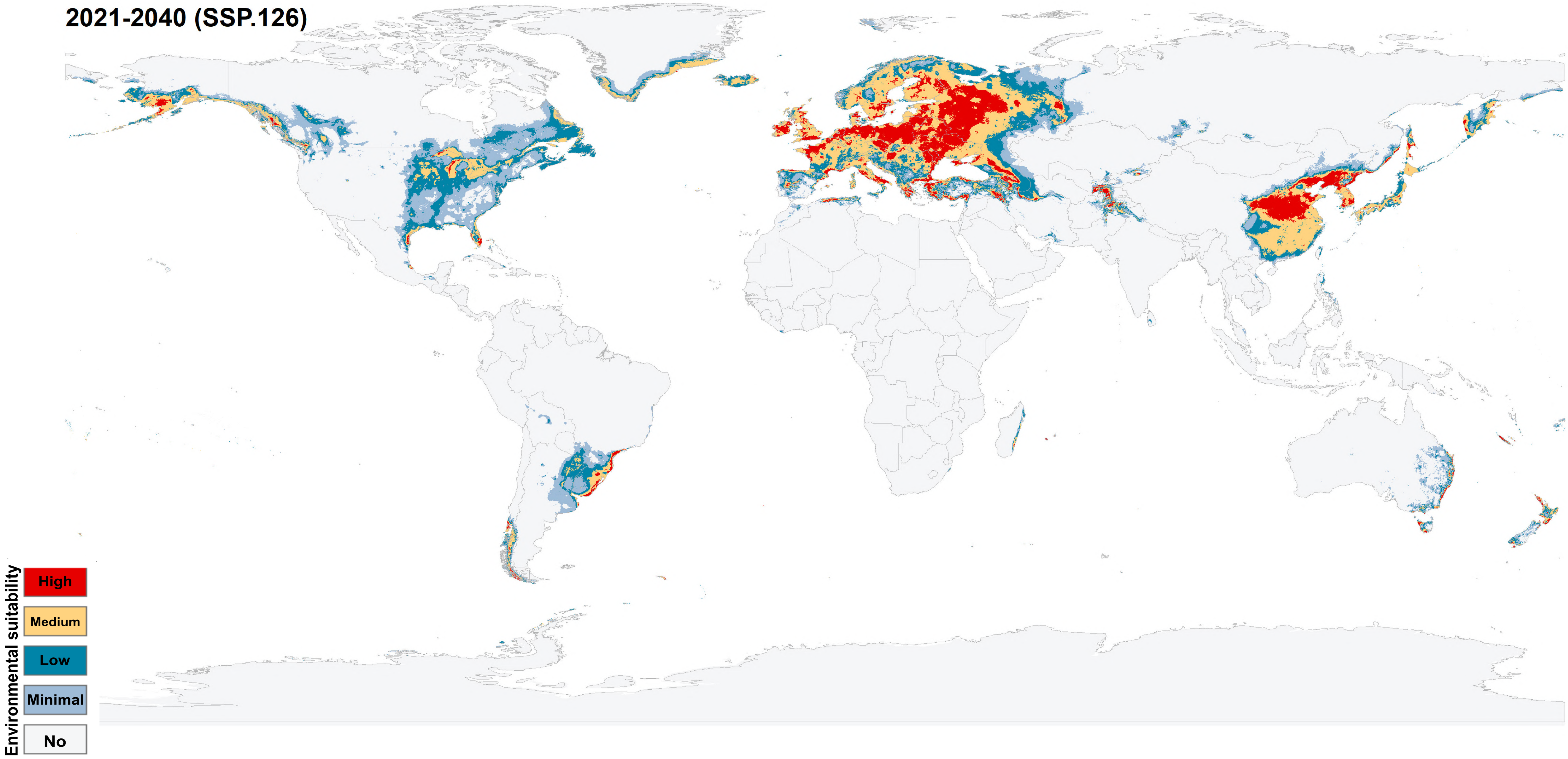
2021-2040 (SSP.126)
Environmental suitability
High
Medium
Low
Minimal
No

2021-2040 (SSP.245)

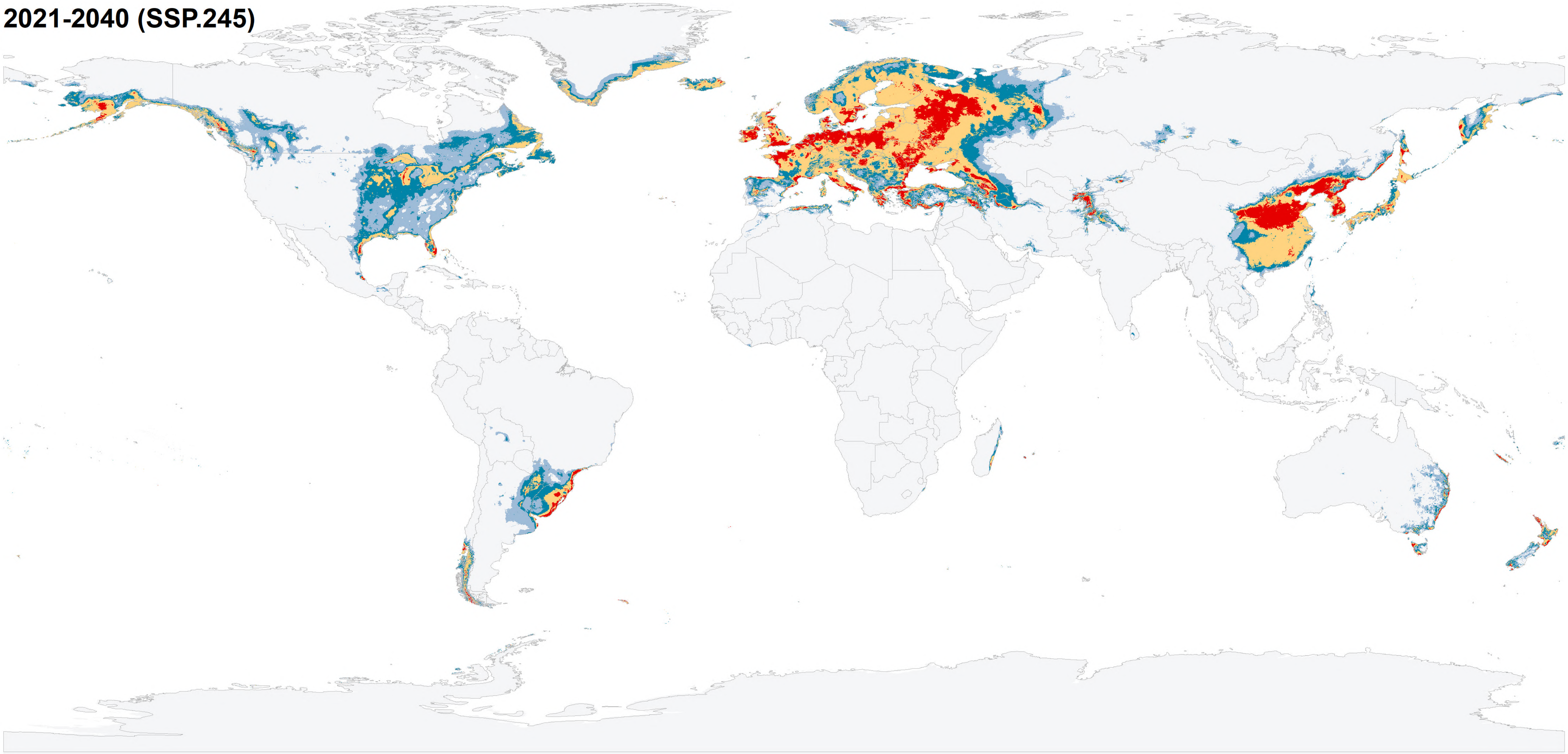

2021-2040 (SSP.370)

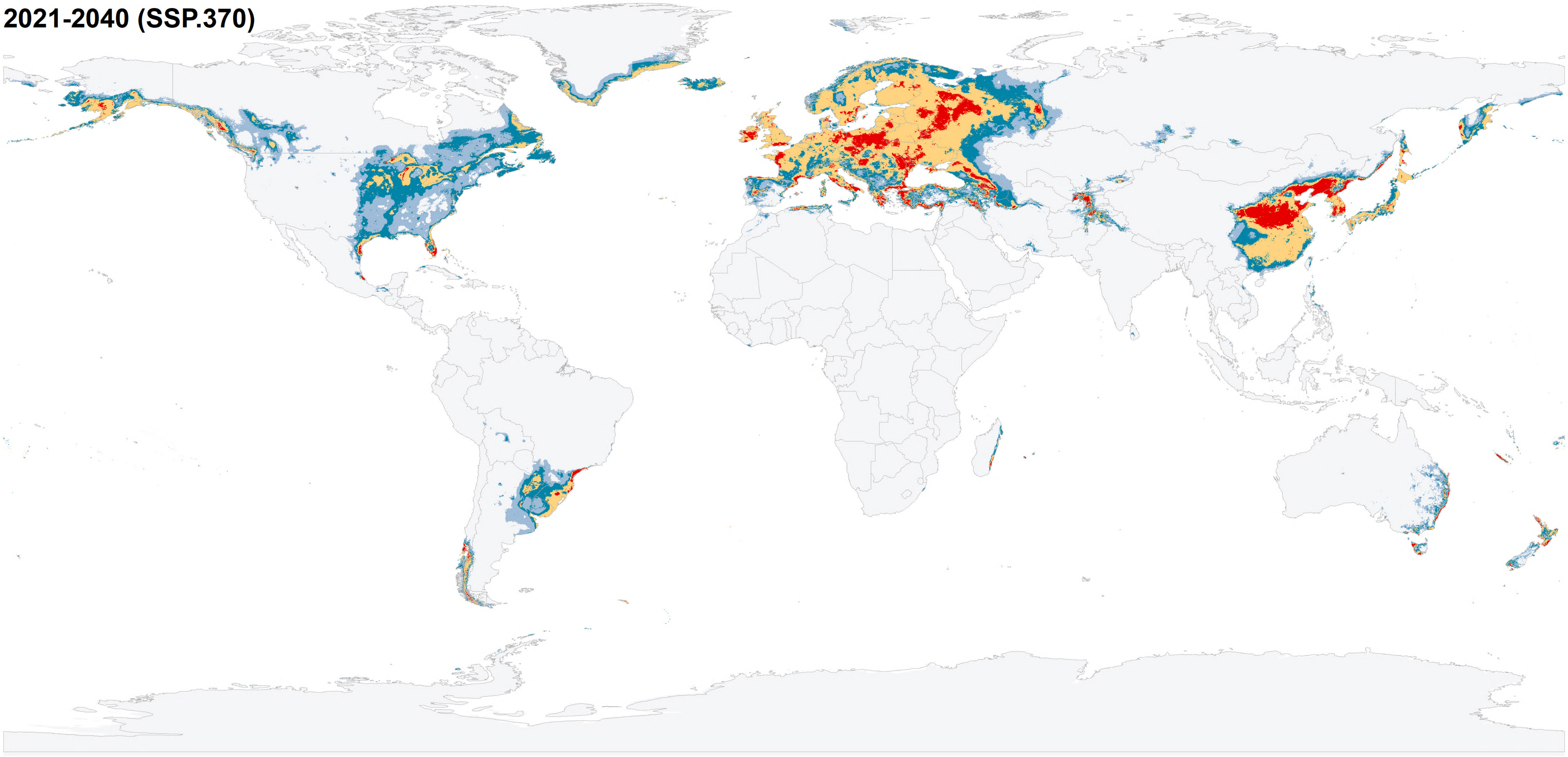

2021-2040 (SSP.585)

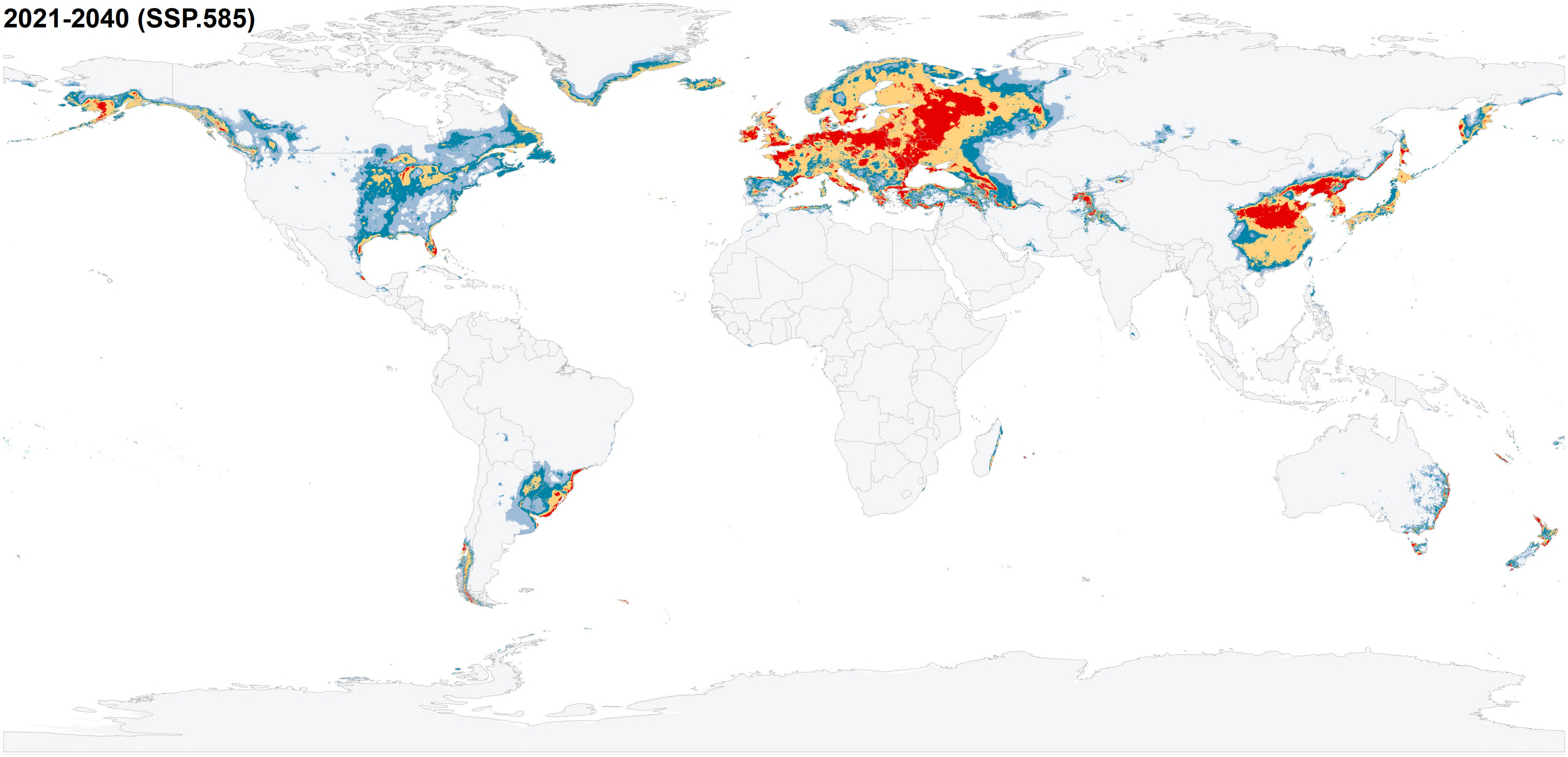

2041-2060 (SSP.126)

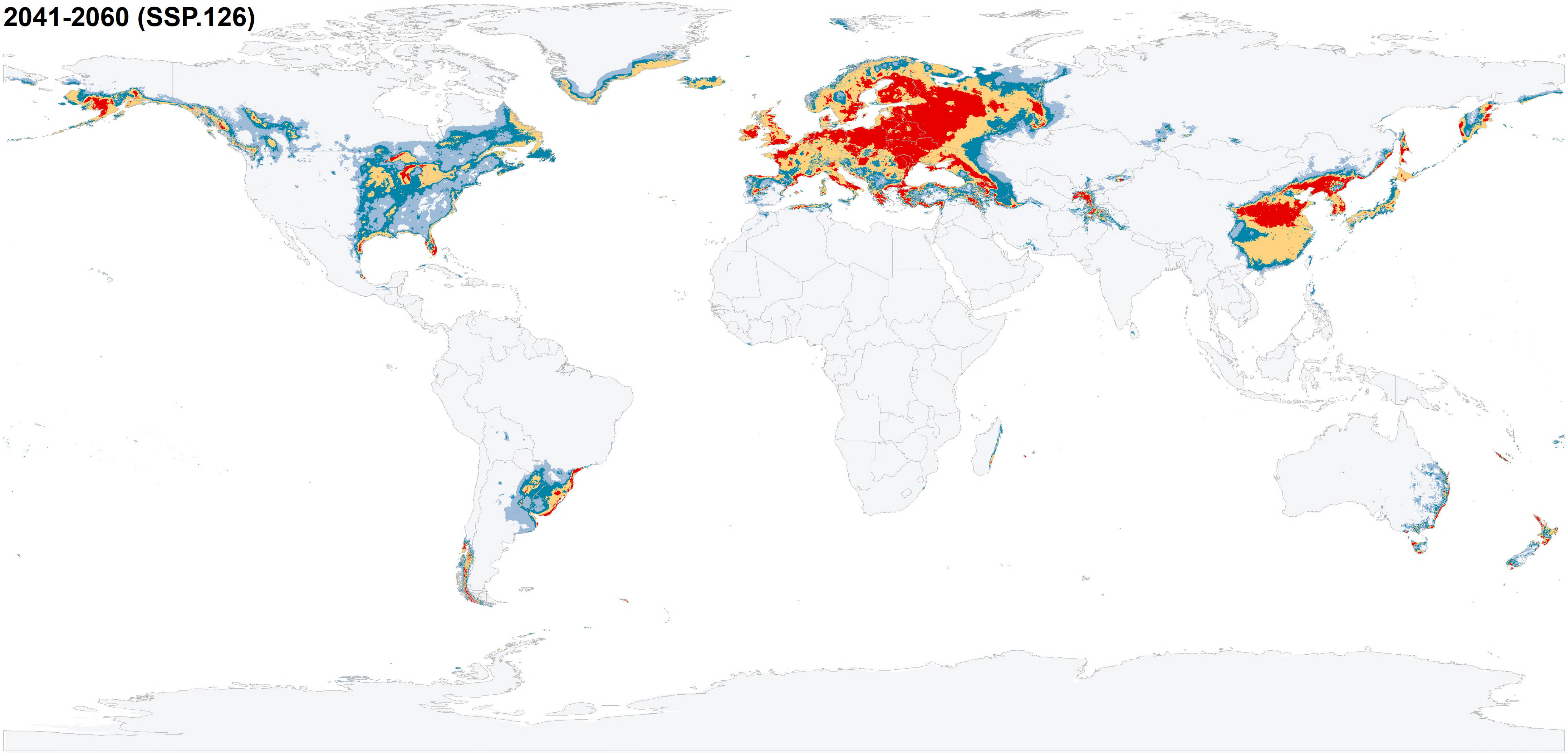

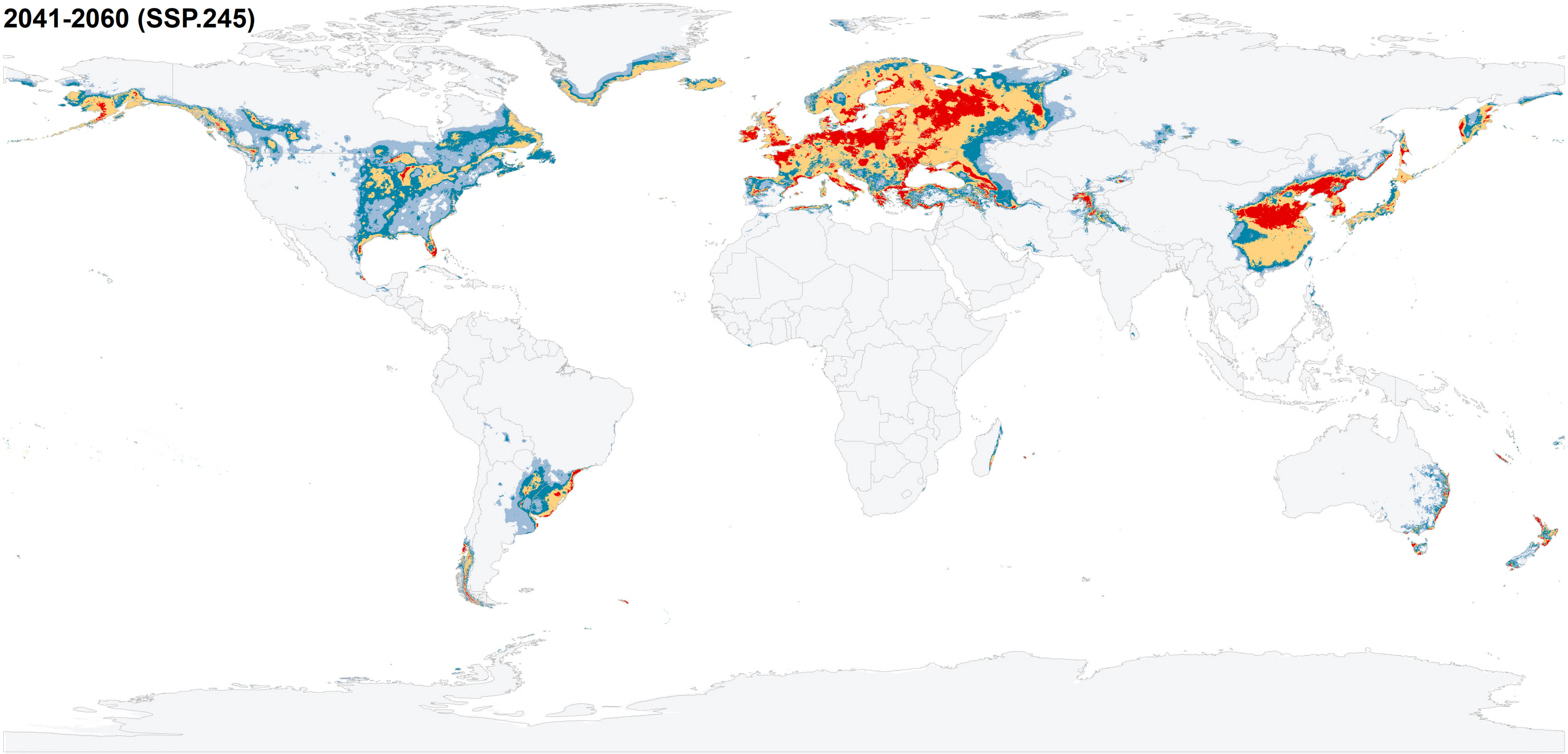
2041-2060 (SSP.245)

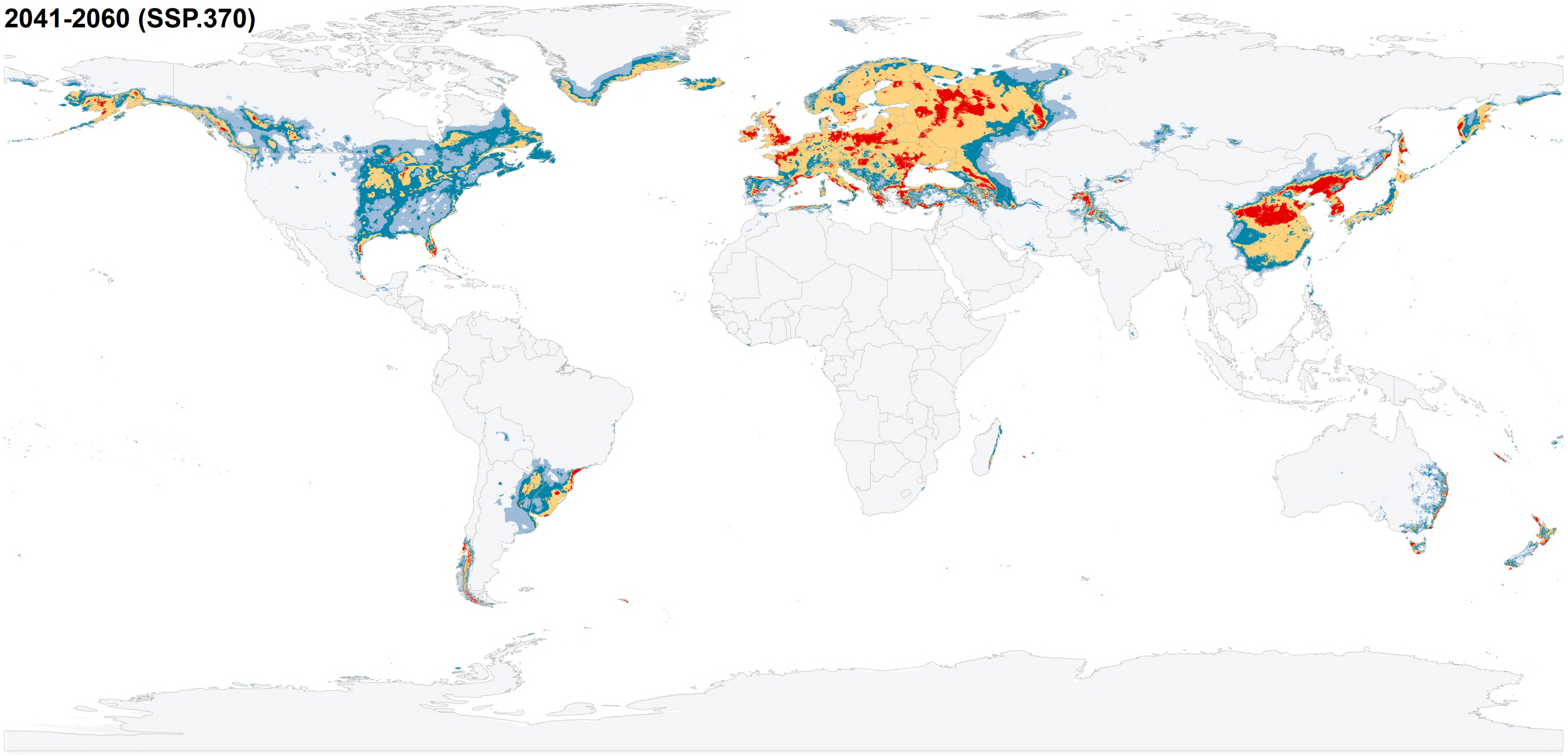
2041-2060 (SSP.370)

2041-2060 (SSP.585)

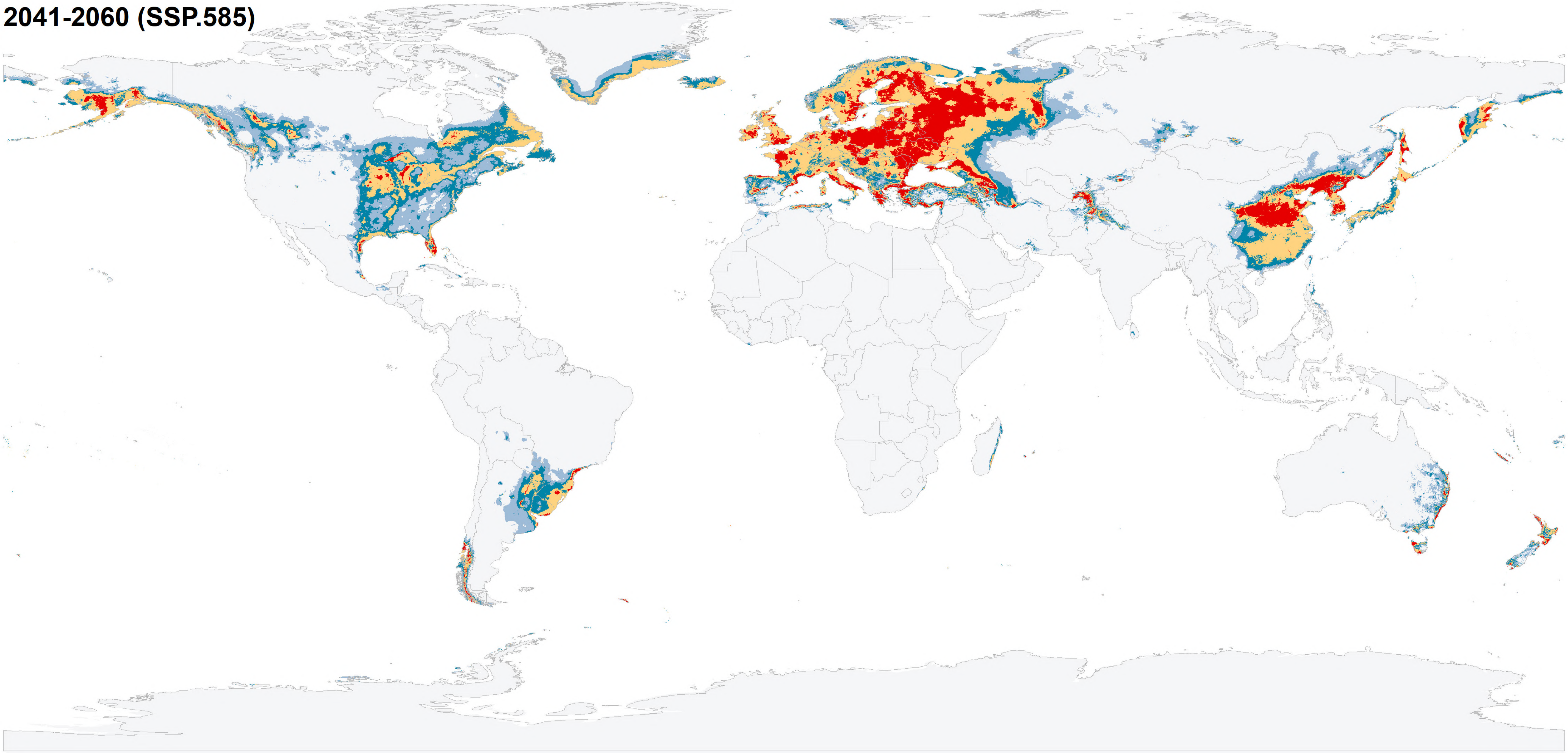

2061-2080 (SSP.126)

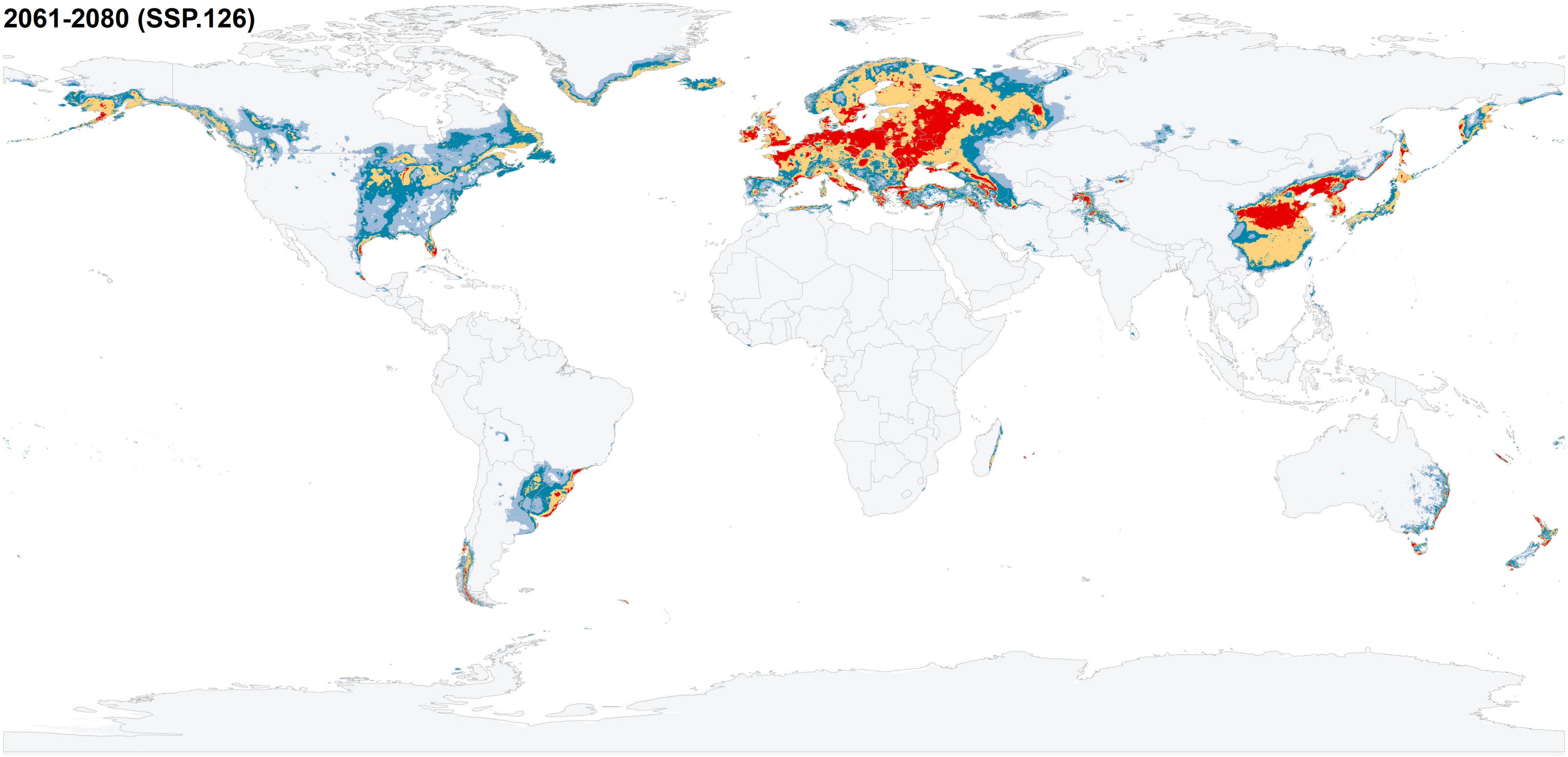

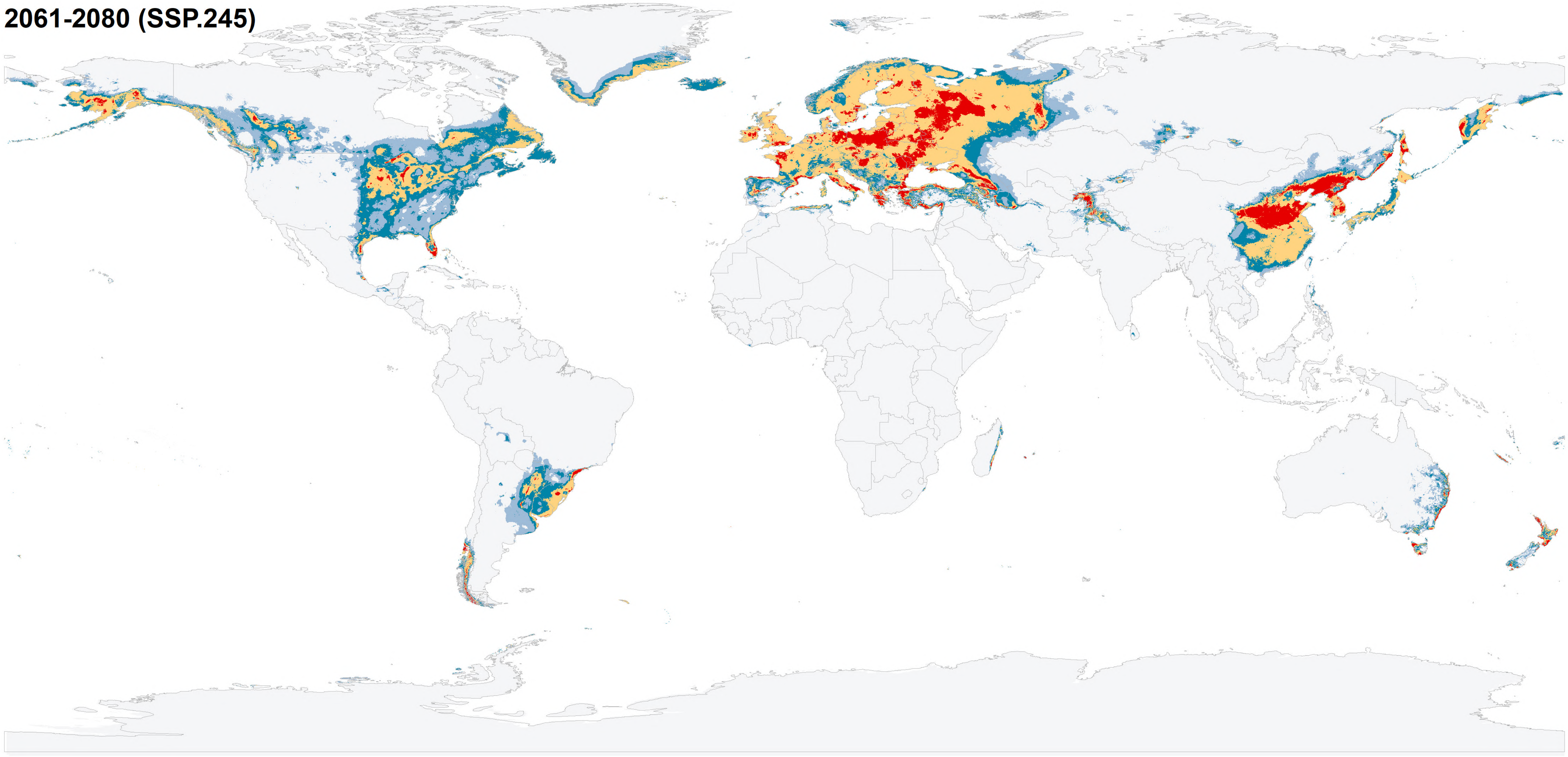
2061-2080 (SSP.245)

2061-2080 (SSP.370)

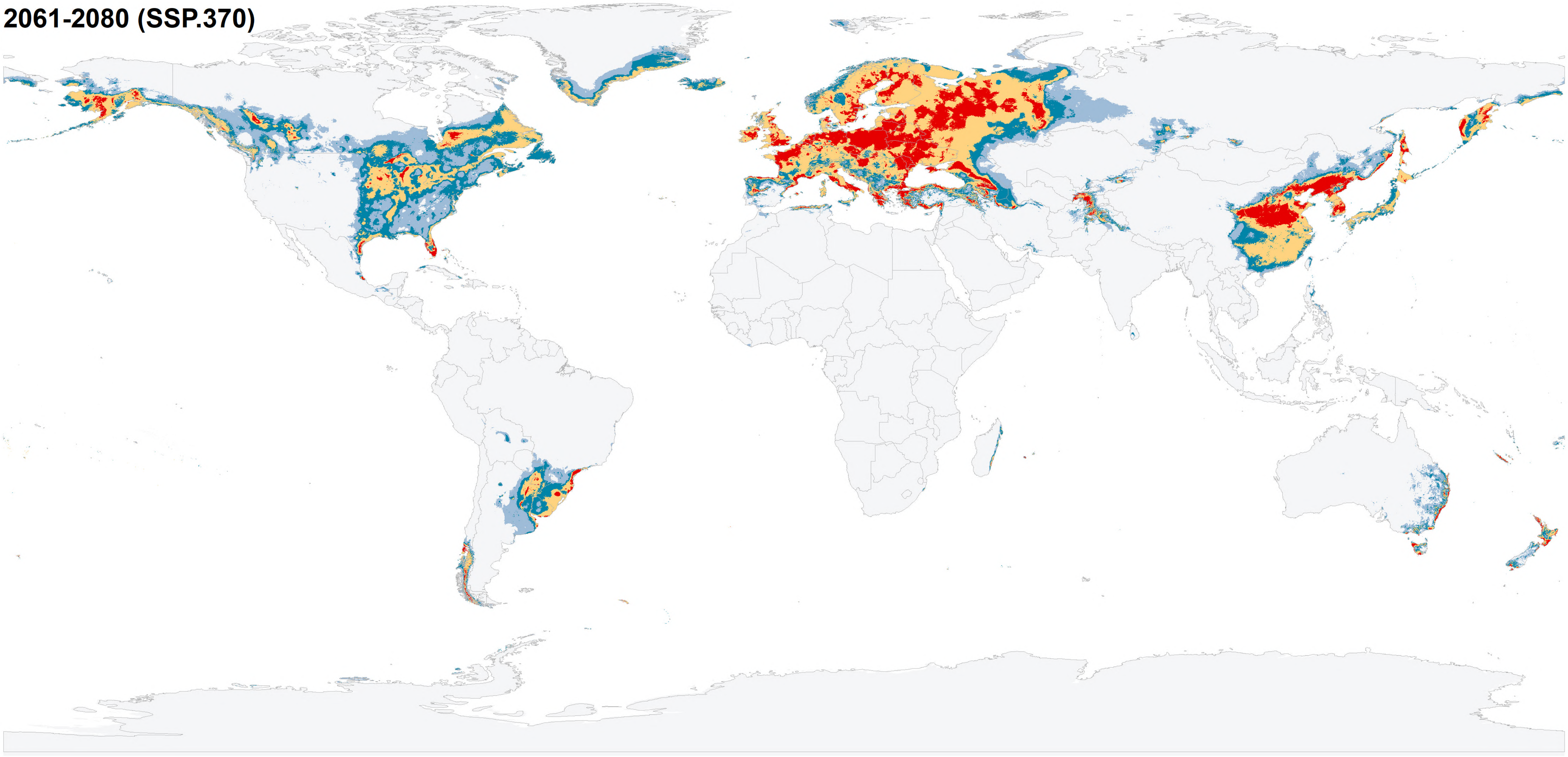

2061-2080 (SSP.585)

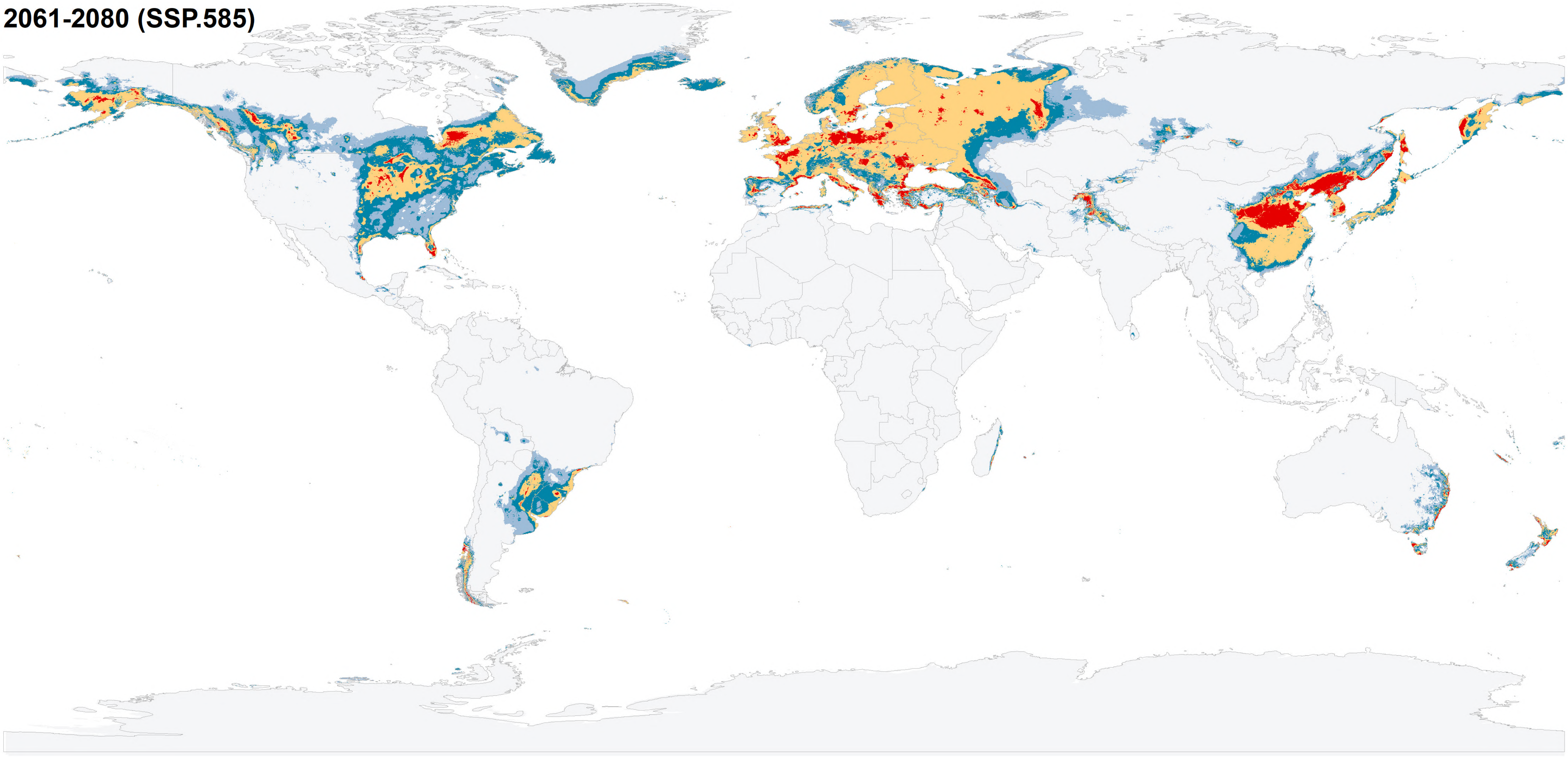

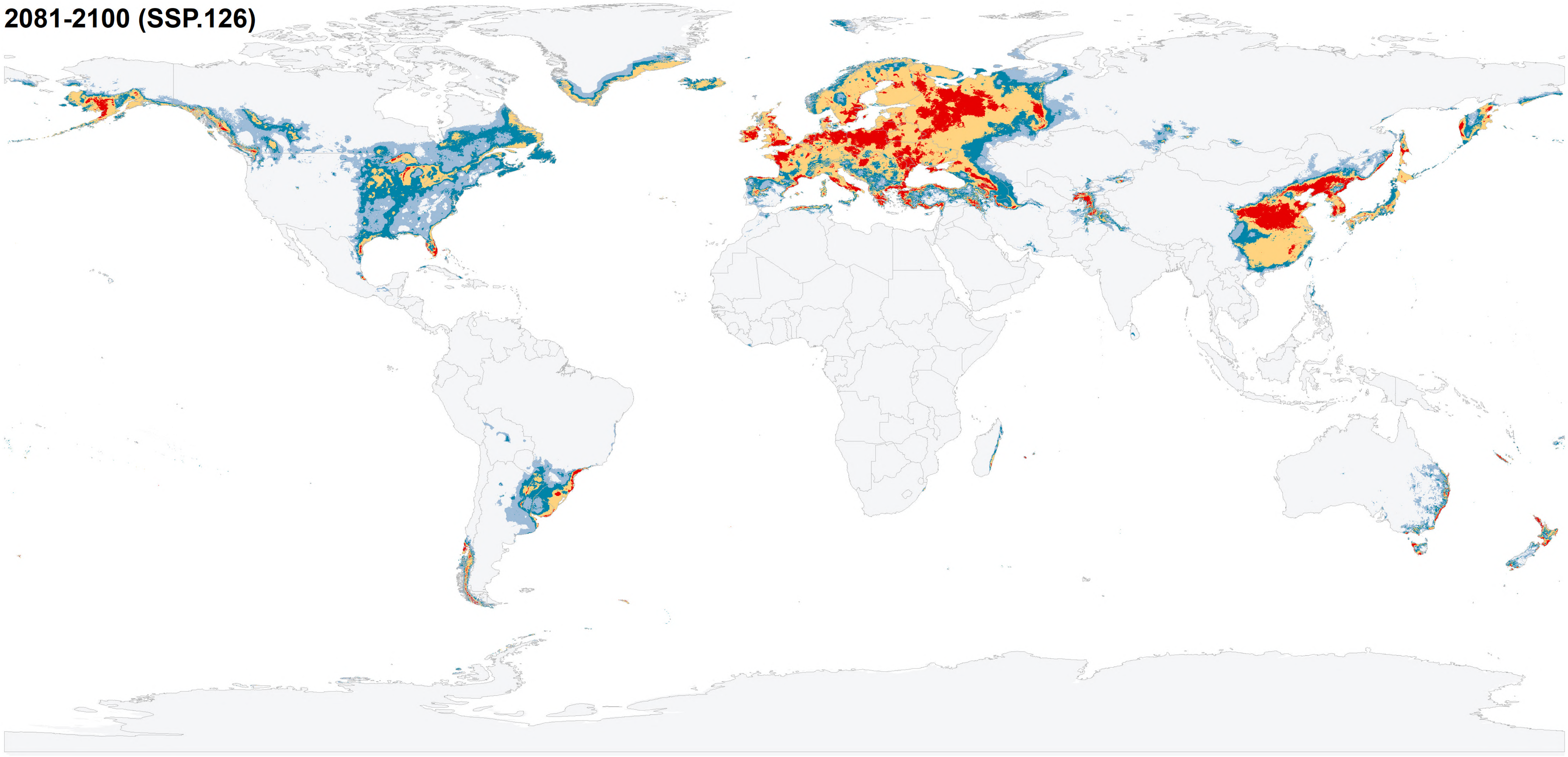
2081-2100 (SSP.126)

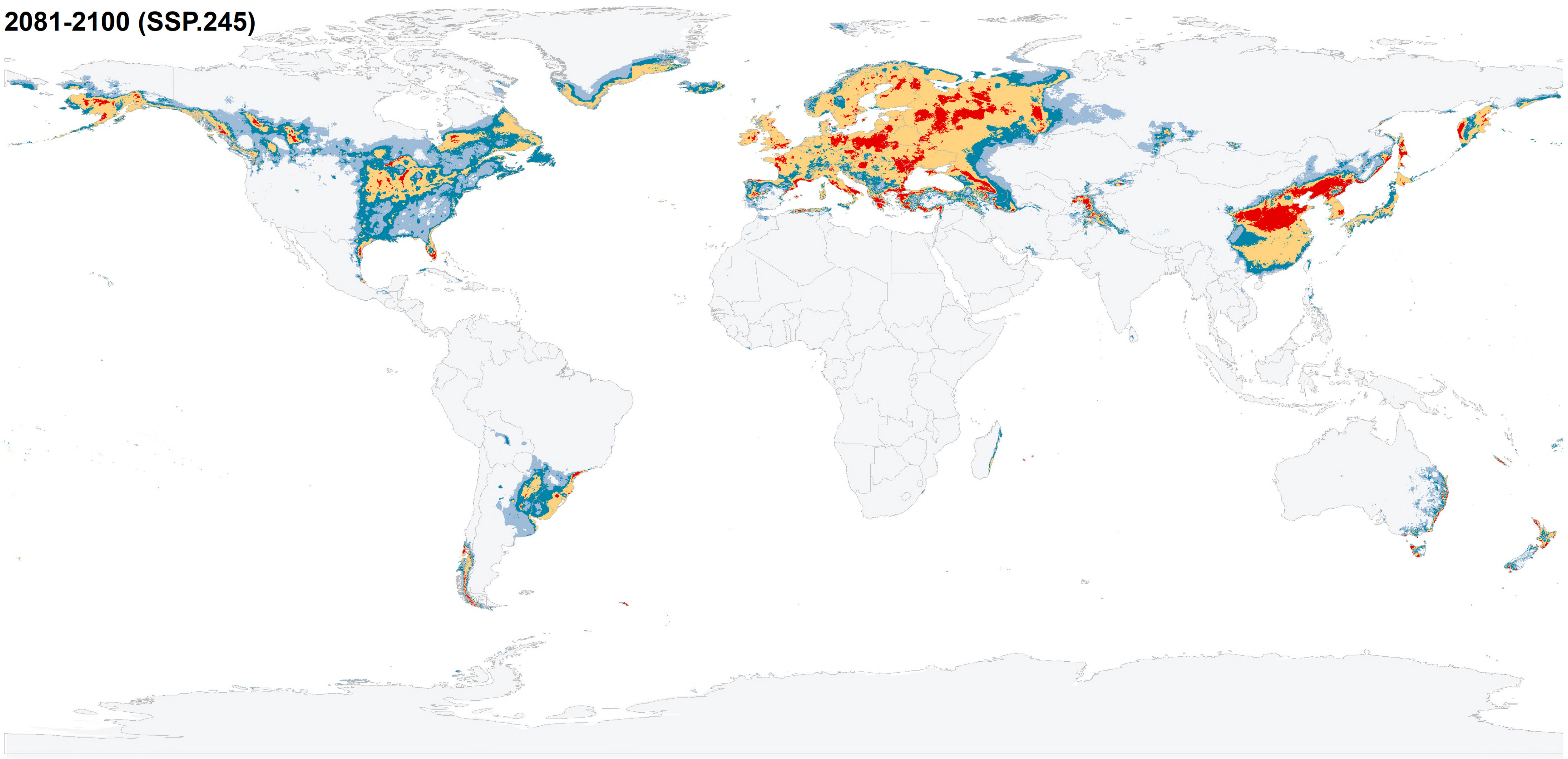
2081-2100 (SSP.245)

2081-2100 (SSP.370)

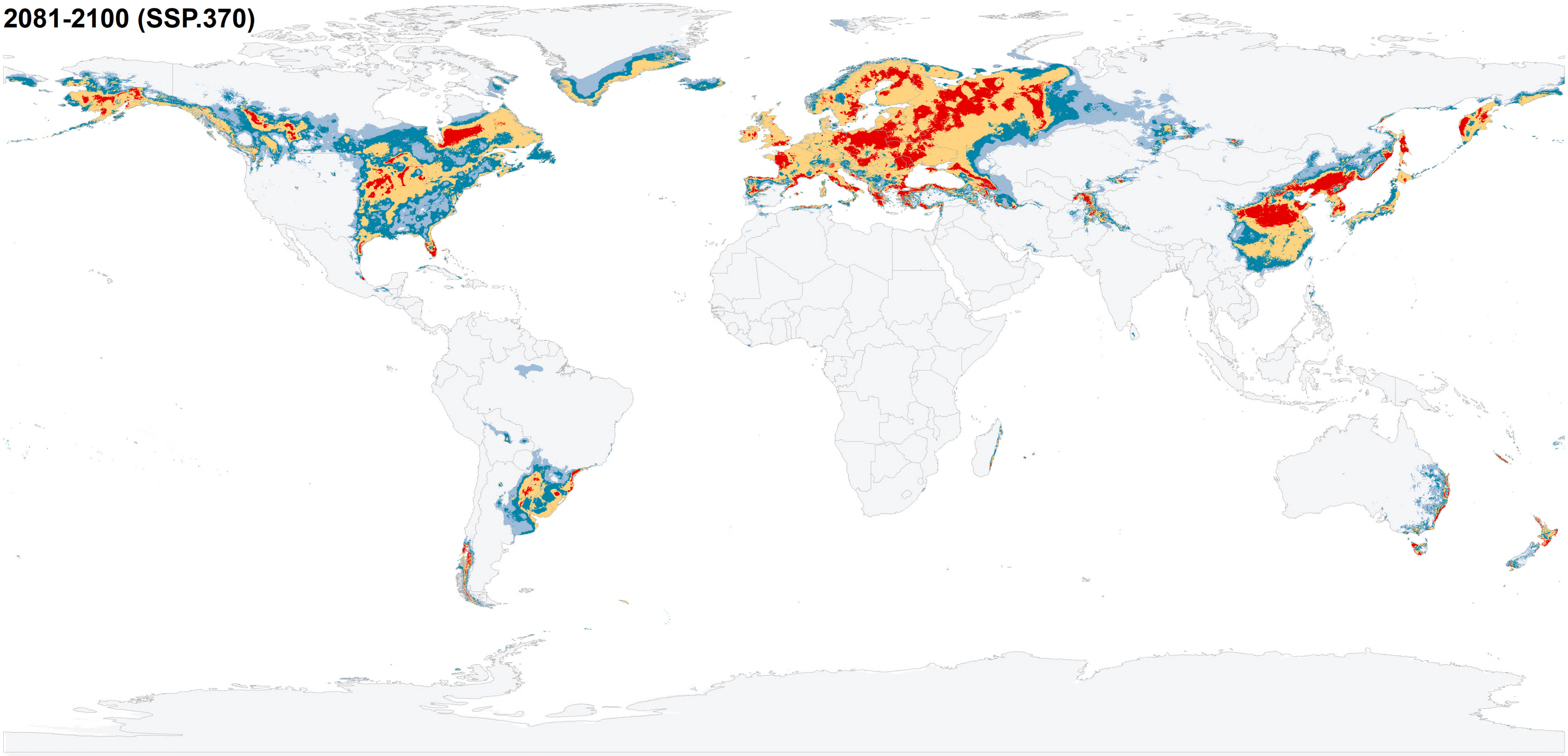

2081-2100 (SSP.585)

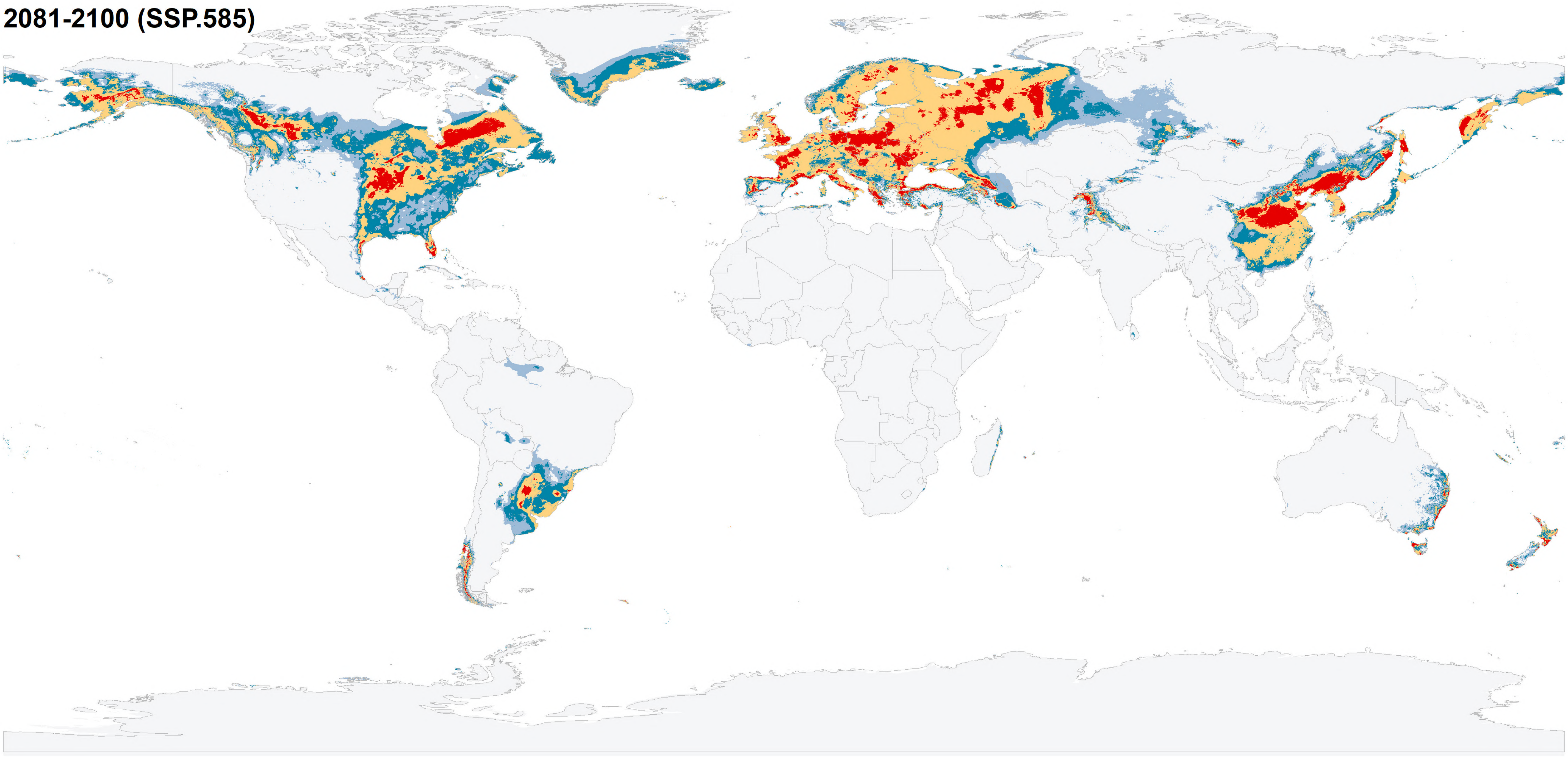

Supplement: Supplementary file 3 — Supplementary Material 3 [file 41598_2025_86205_MOESM3_ESM.pdf]
