# Supplementary material for: Climate change influences on the potential geographic distribution of the invasive Asian longhorned tick, Haemaphysalis longicornis
Source: Sci Rep. 2025 Jan 17;15:2266. doi: 10.1038/s41598-025-86205-6 (PMC11748616; doi:10.1038/s41598-025-86205-6)

S. File 4: The occurrence records used in the One-tailed cumulative binomial probability test for model evaluation Vs. the predicted areas as suitable habitats for *H. longicornis* under historical climatic conditions. (A) Close up to Asia, Oceania, and New Zealand. (B) Close up to the Americas.

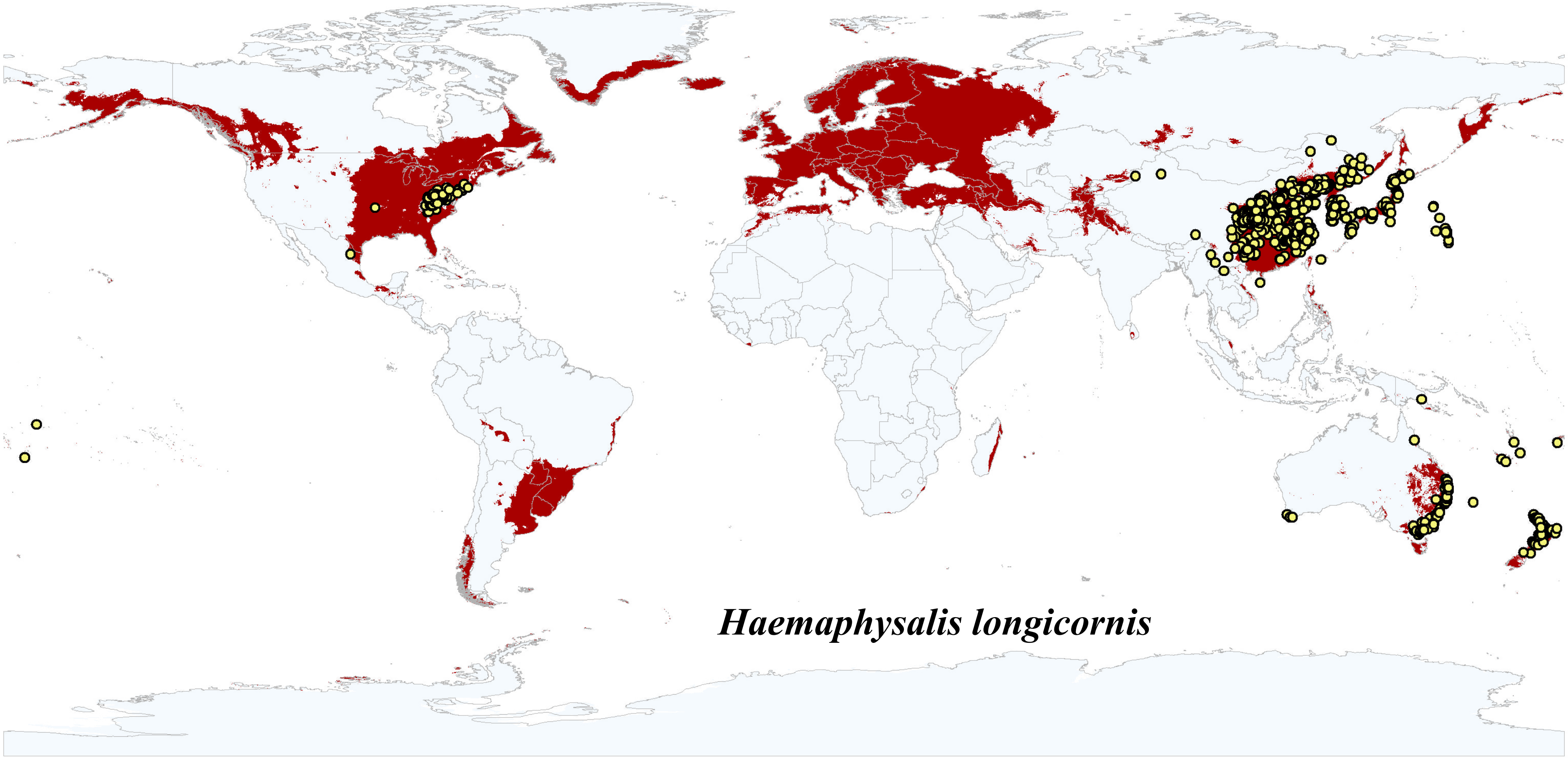
Haemaphysalis longicornis

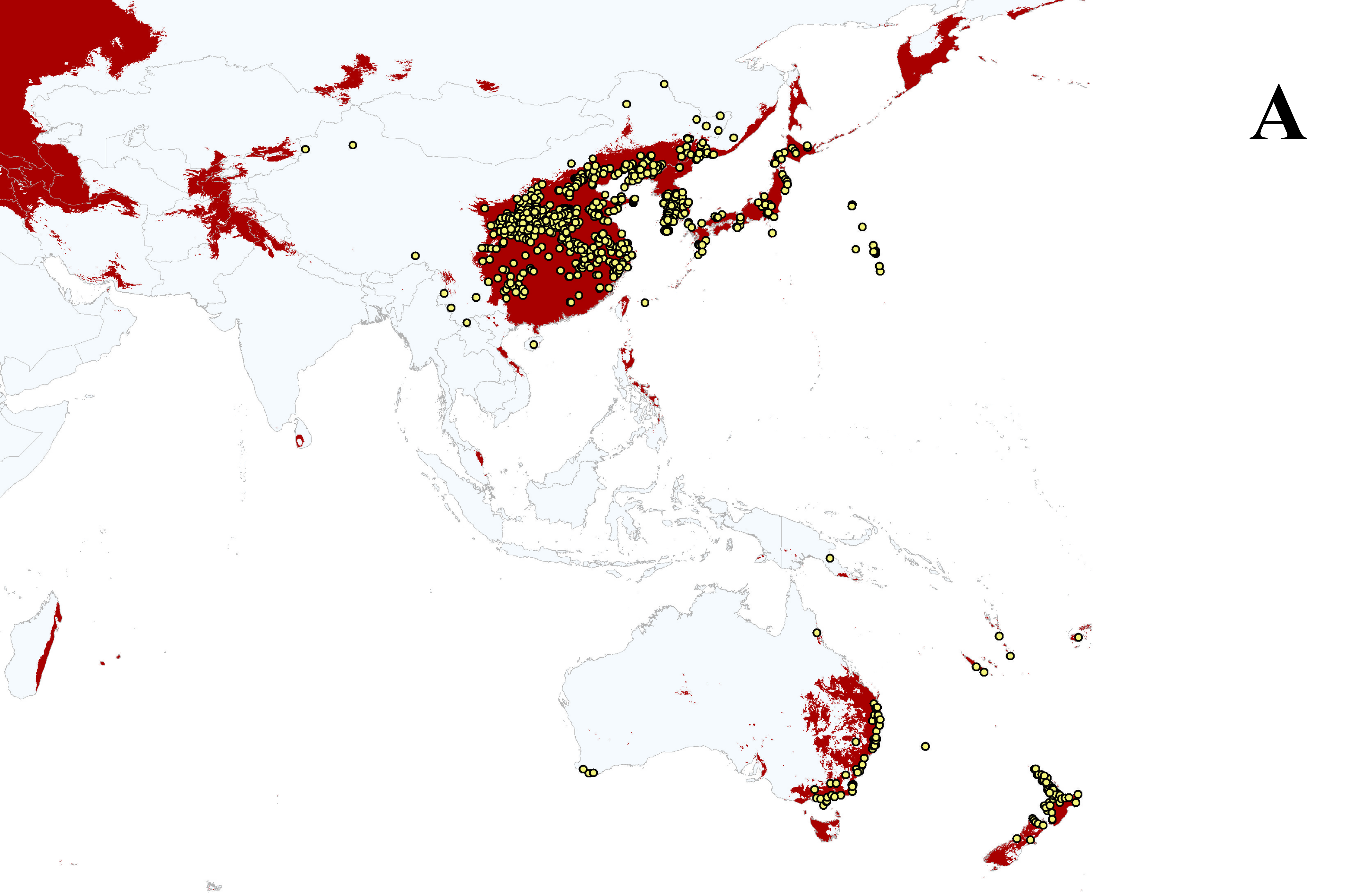
A

B

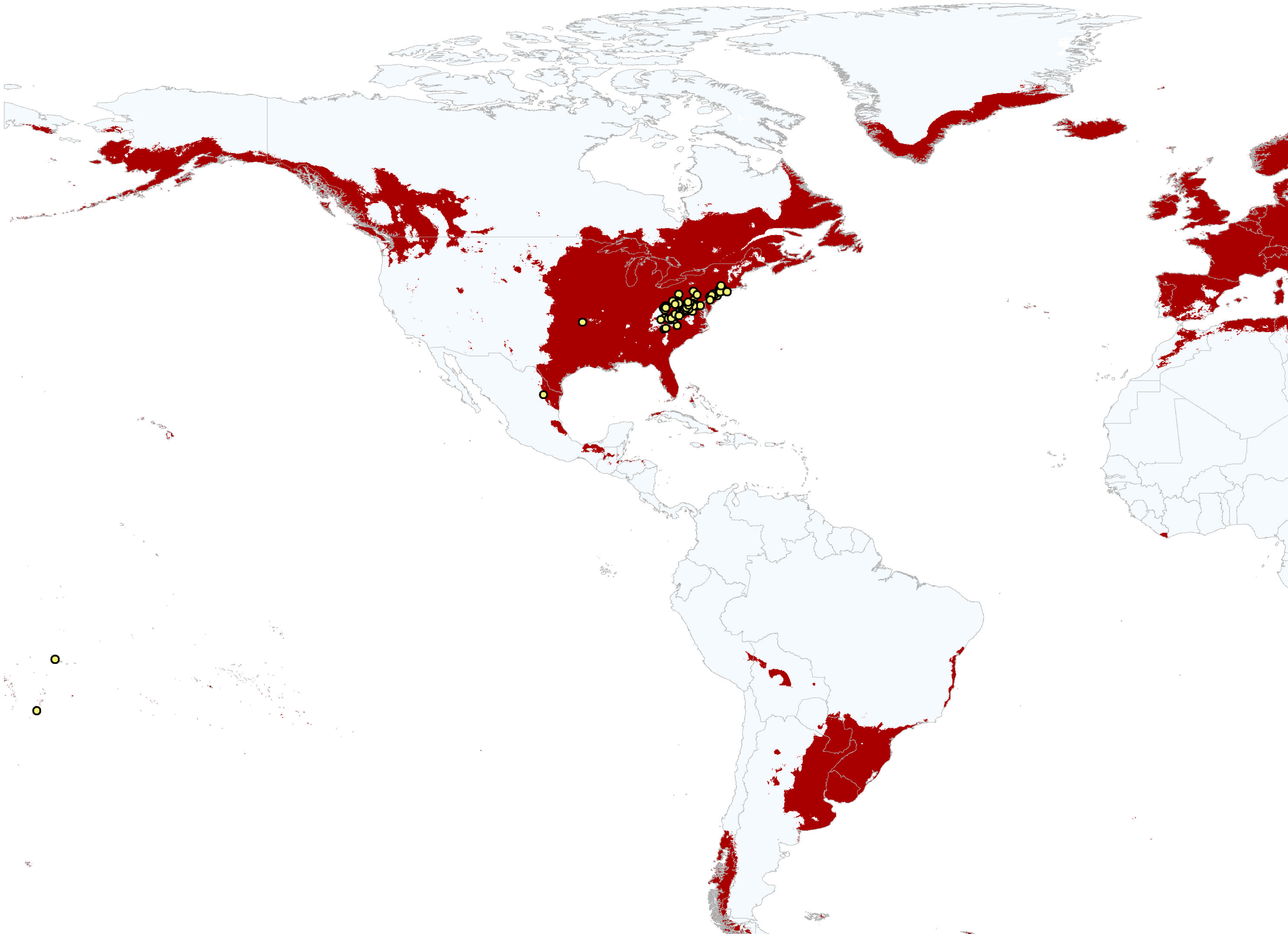

Supplement: Supplementary file 4 — Supplementary Material 4 [file 41598_2025_86205_MOESM4_ESM.pdf]
