# Supplementary material for: Climate change influences on the potential geographic distribution of the invasive Asian longhorned tick, Haemaphysalis longicornis
Source: Sci Rep. 2025 Jan 17;15:2266. doi: 10.1038/s41598-025-86205-6 (PMC11748616; doi:10.1038/s41598-025-86205-6)
Supplement: Supplementary file 5 — Supplementary Material 5 [file 41598_2025_86205_MOESM5_ESM.pdf]

S. File 5: The file presents a binary model of *Haemaphysalis longicornis* under historical climatic conditions, where red-shaded areas indicate suitable environments and white areas denote non-suitable regions. Additionally, the file includes maps showing shifts in environmental suitability under changing climate scenarios across four future time periods: 2021-2040, 2041-2060, 2061-2080, and 2081-2100. These projections are based on four Shared Socioeconomic Pathways (SSPs): SSP126, SSP245, SSP370, and SSP585. The color-coded maps depict various scenarios: white represents areas with no environmental suitability both historically and in future conditions, pale yellow indicates stability in environmental suitability across time, blue shows regions where suitability is predicted to contract, and red highlights areas where suitable environments are expected to expand. The maps are derived from thresholded models, providing a detailed visualization of how *H. longicorn's* potential habitat may shift in response to climate change.

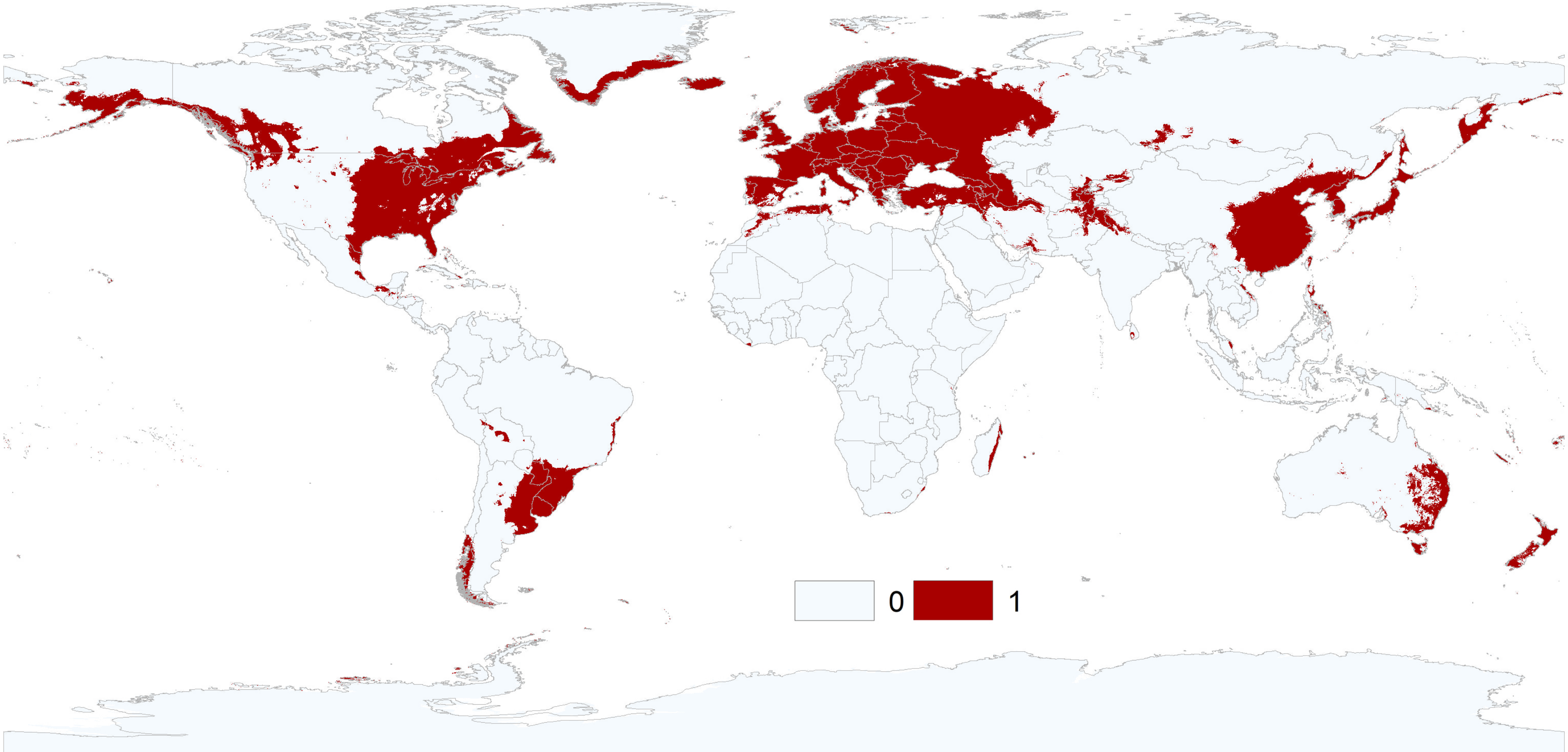

0
1

Haemaphysalis longicornis
2021-2040

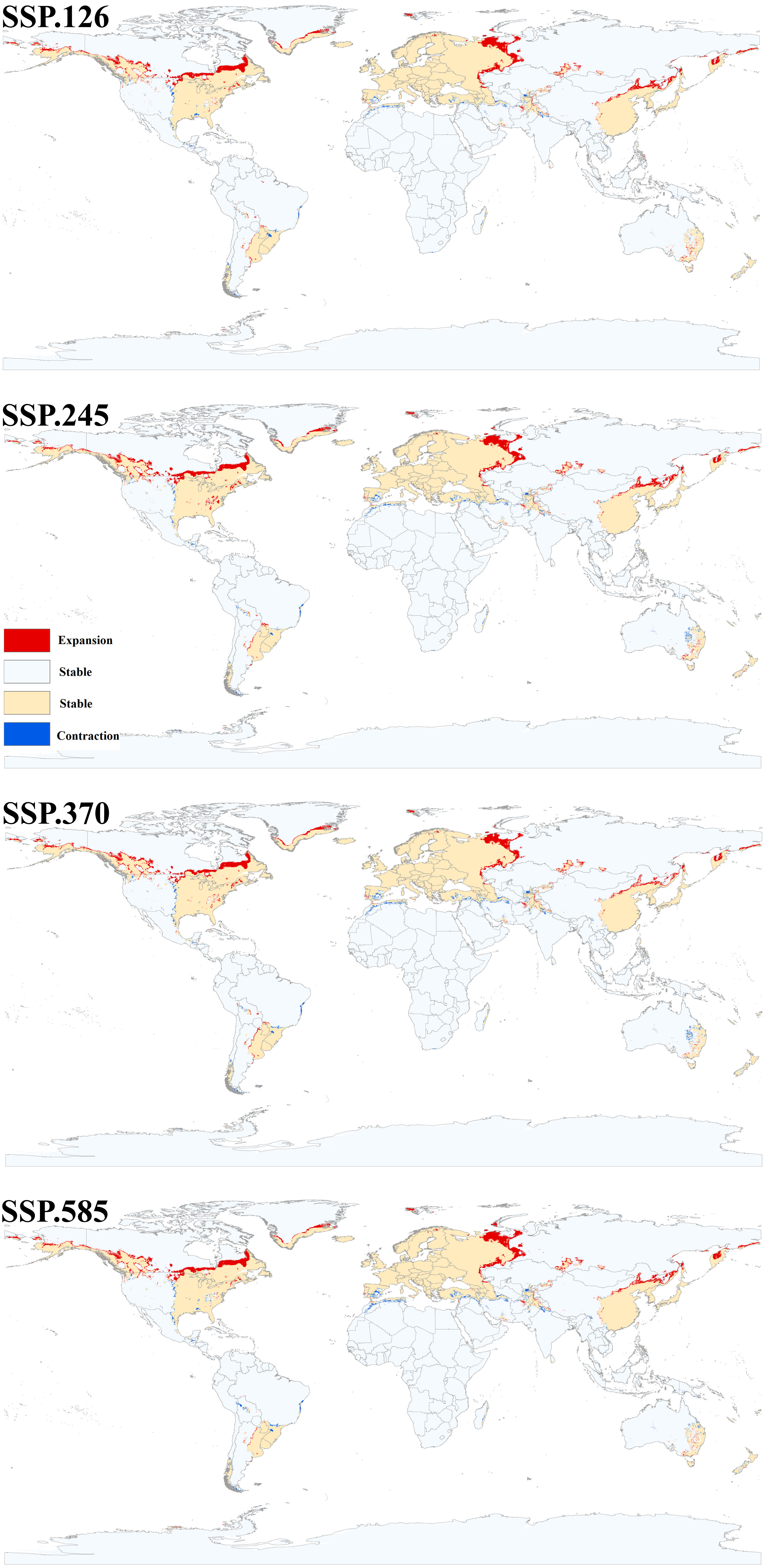

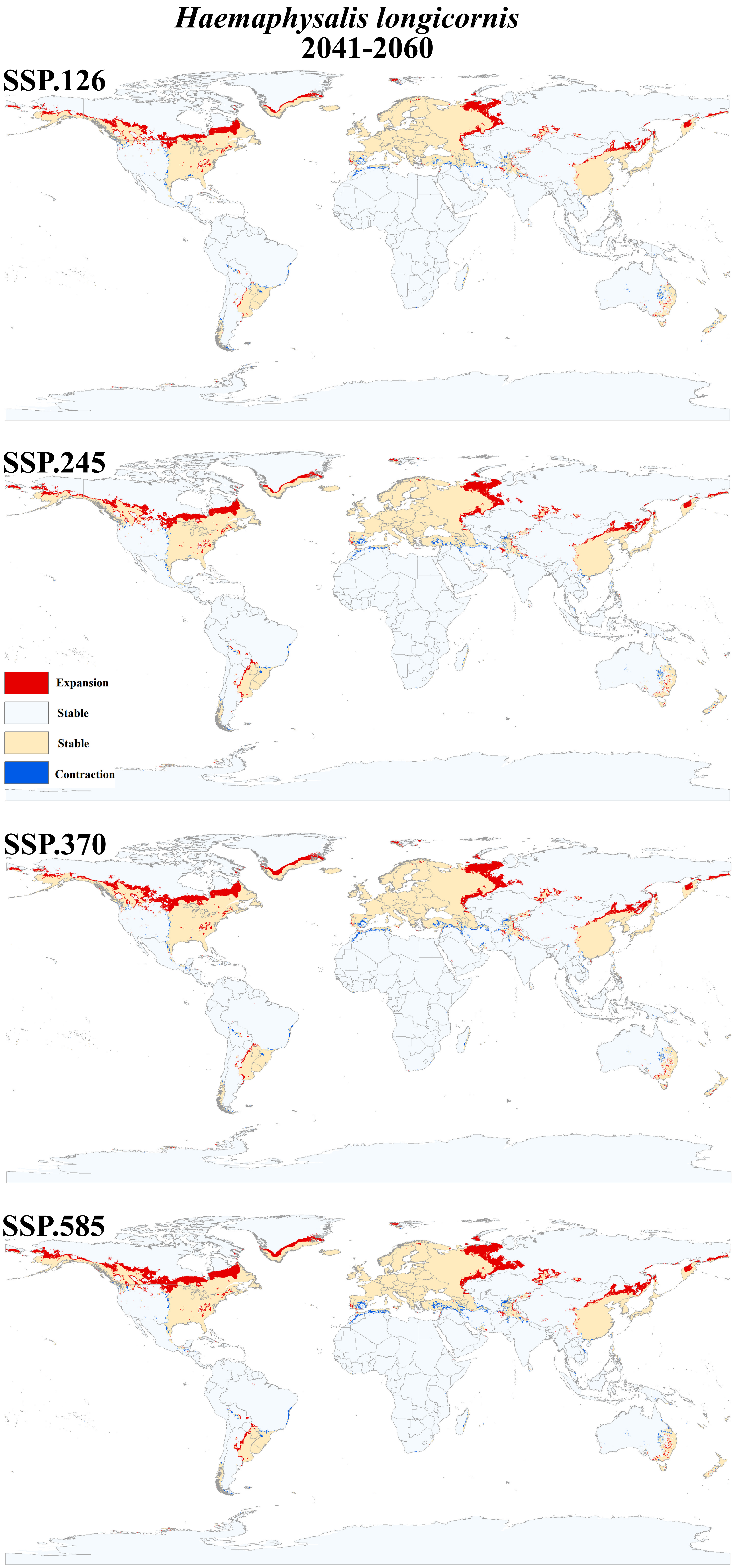

Haemaphysalis longicornis
2041-2060
SSP.126
SSP.245
Expansion
Stable
Stable
Contraction
SSP.370
SSP.585

Haemaphysalis longicornis
2061-2080

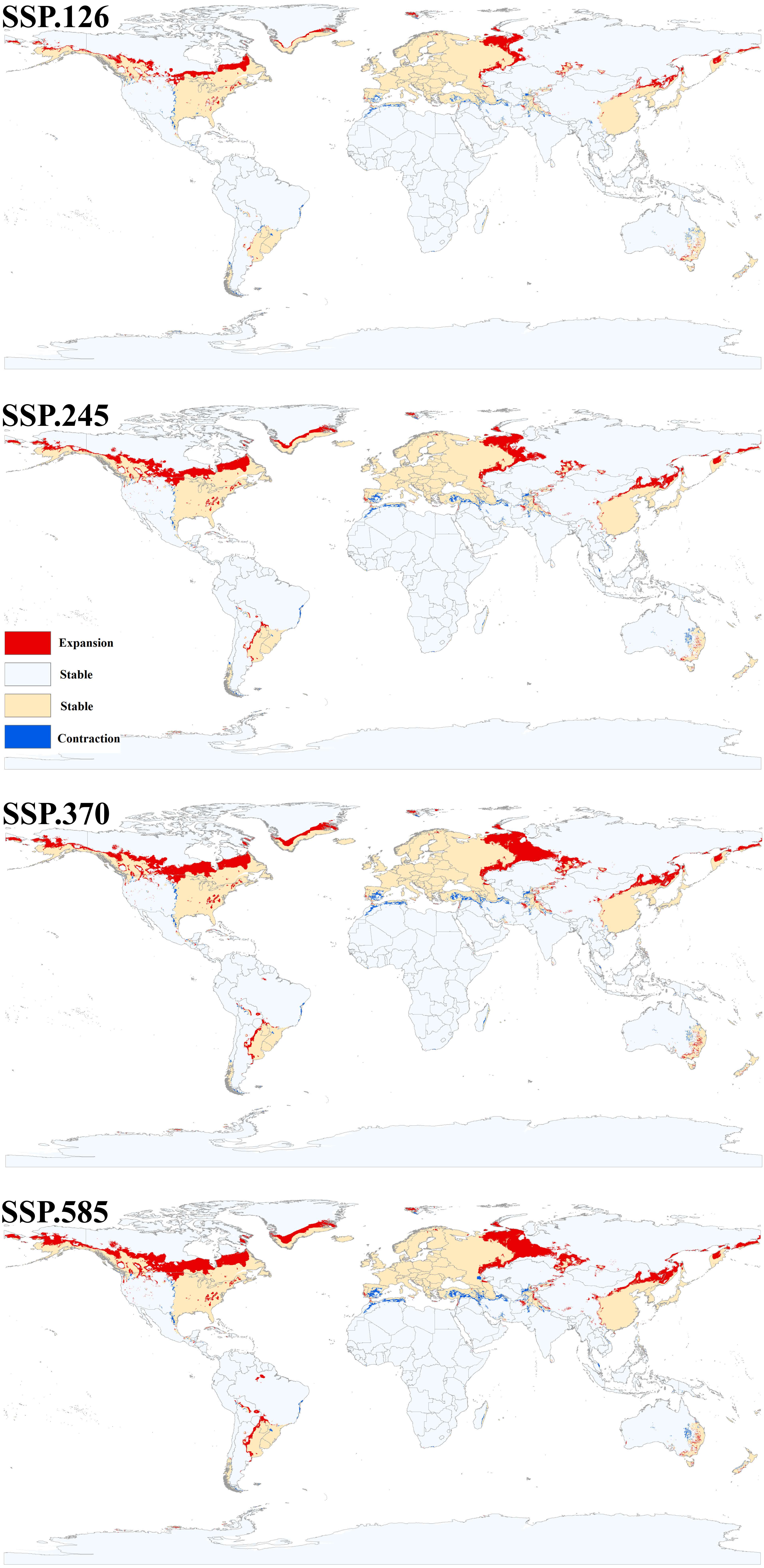

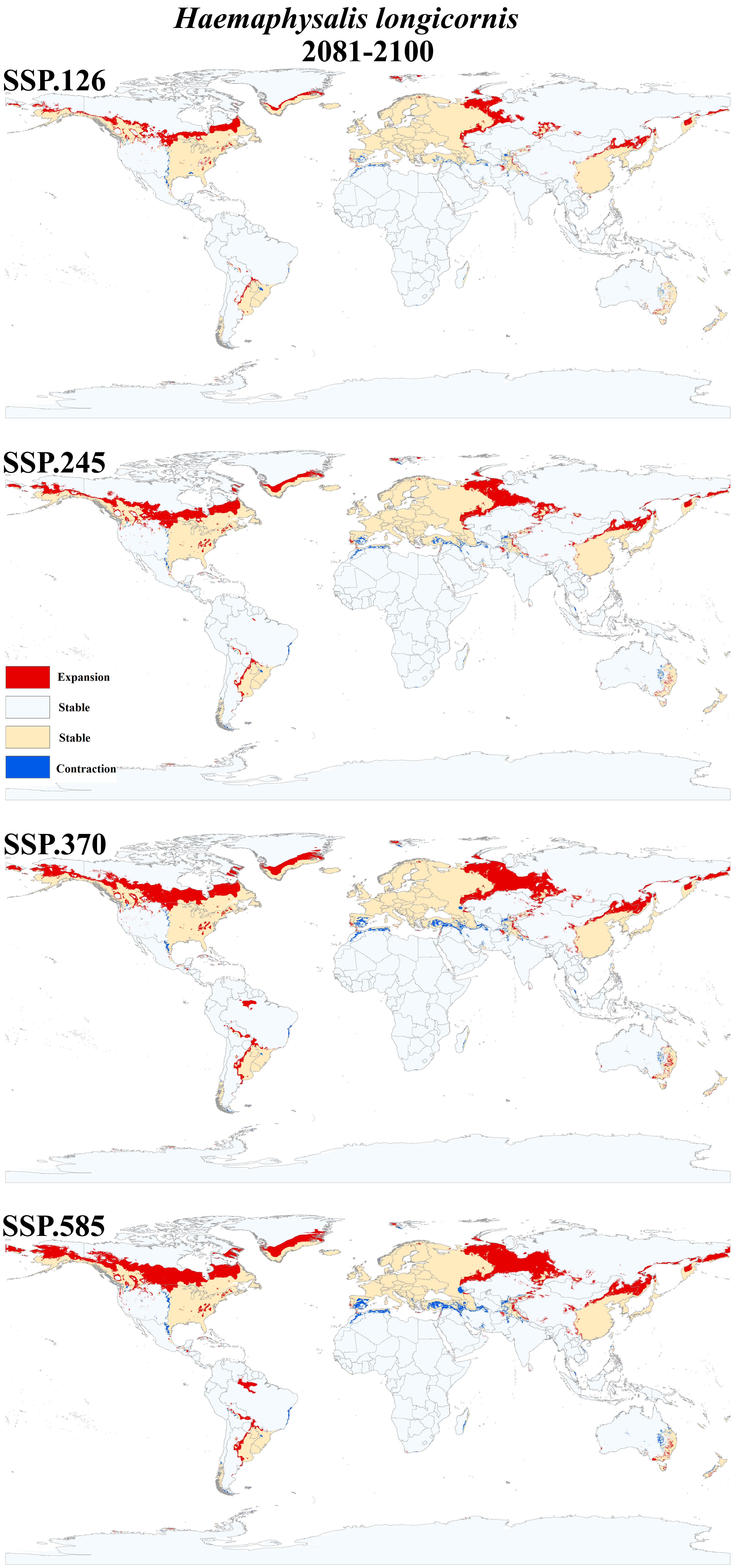
Haemaphysalis longicornis
2081-2100
SSP.126
SSP.245
Expansion
Stable
Stable
Contraction
SSP.370
SSP.585
