# Supplementary material for: Climate change influences on the potential geographic distribution of the invasive Asian longhorned tick, Haemaphysalis longicornis
Source: Sci Rep. 2025 Jan 17;15:2266. doi: 10.1038/s41598-025-86205-6 (PMC11748616; doi:10.1038/s41598-025-86205-6)
Supplement: Supplementary file 6 — Supplementary Material 6 [file 41598_2025_86205_MOESM6_ESM.pdf]

S. File 6: The uncertainty estimates in the ENMs of *H. longicornis* based on future climatic conditions (2021-2100). The uncertainty estimates associated with the ENMs of future conditions are the difference in anticipation between the different 8 general circulation models used in the present work.

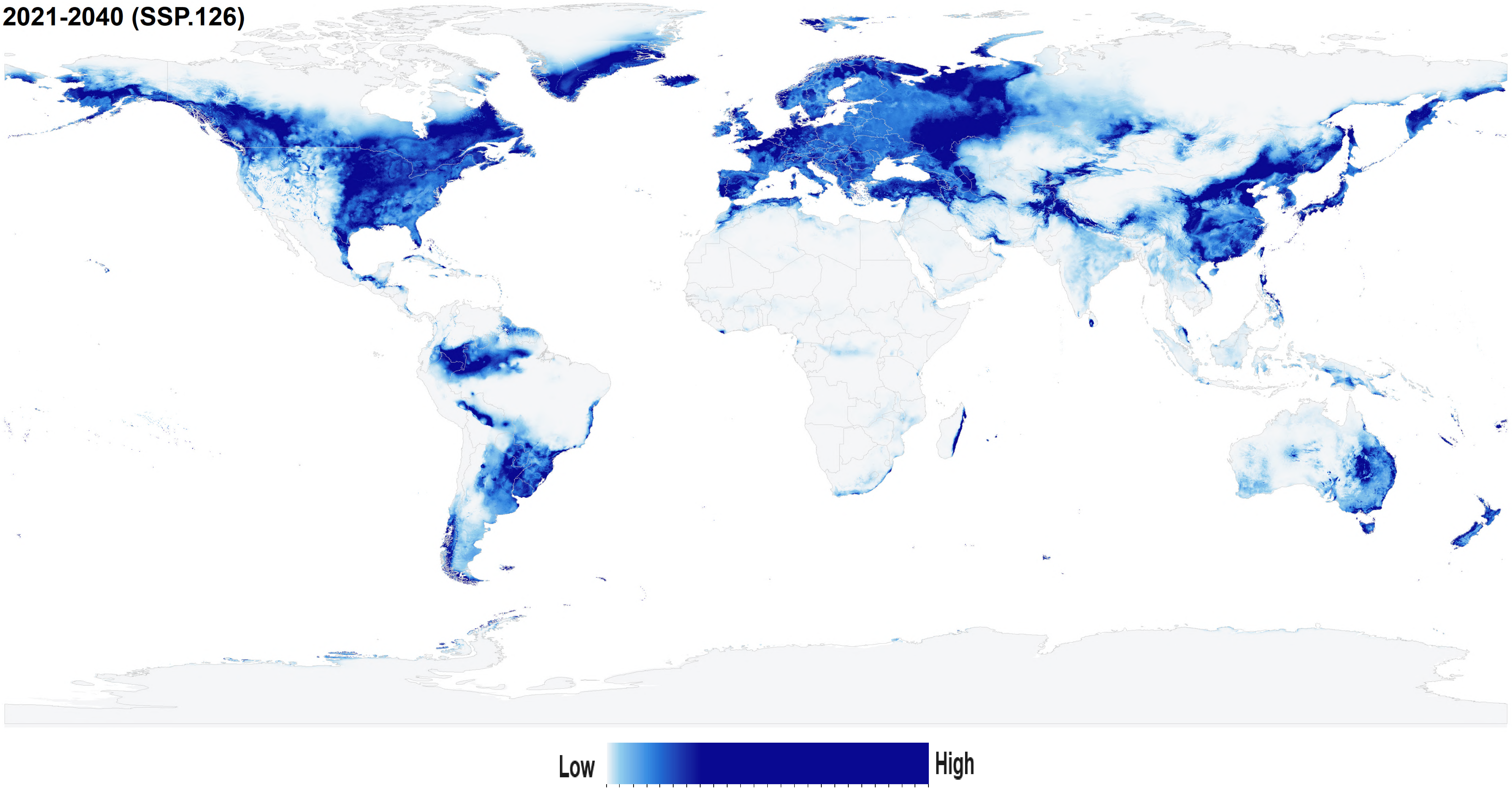
2021-2040 (SSP.126)
Low
High

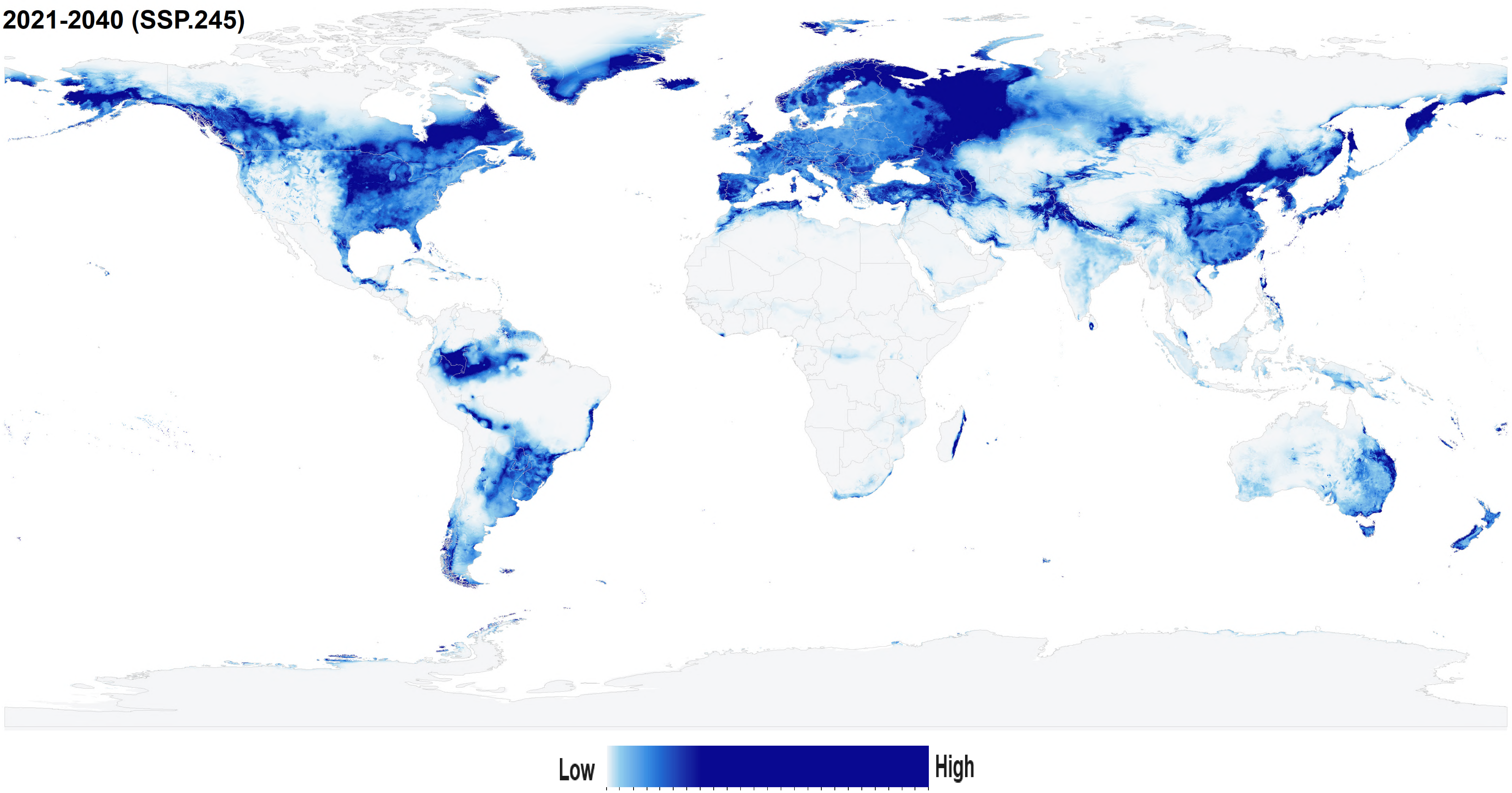
2021-2040 (SSP.245)
Low
High

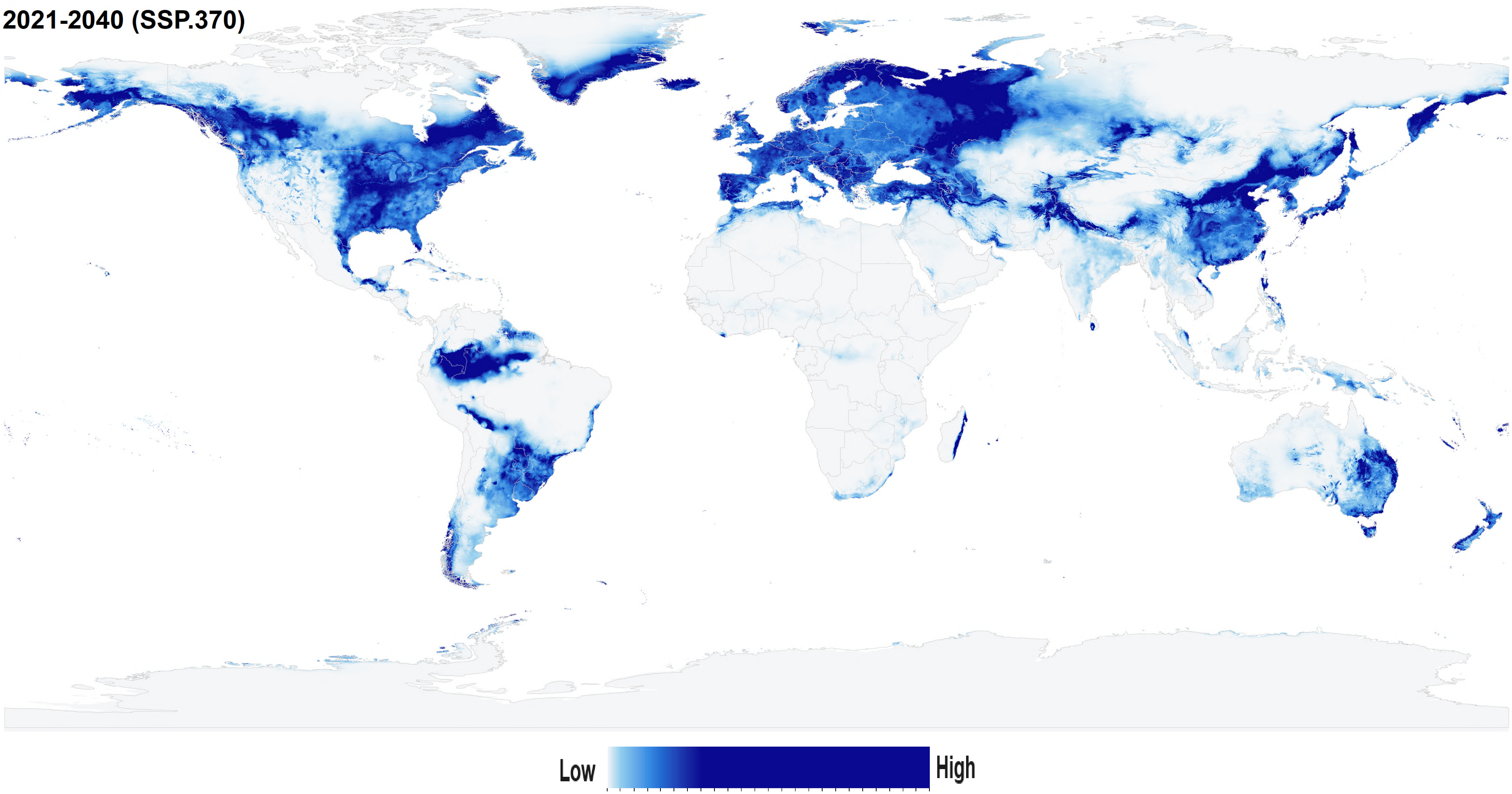
2021-2040 (SSP.370)
Low
High

2021-2040 (SSP.585)

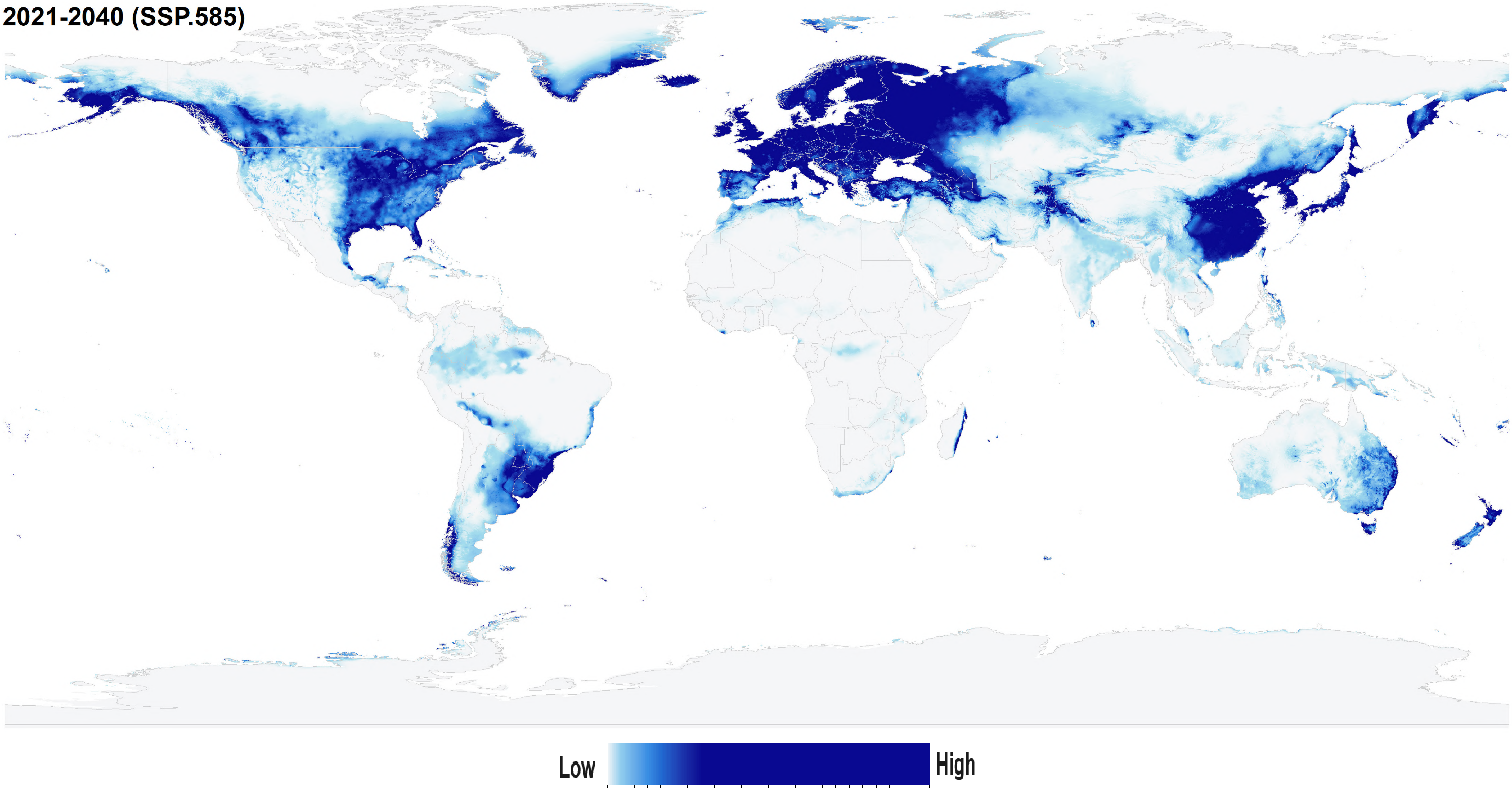

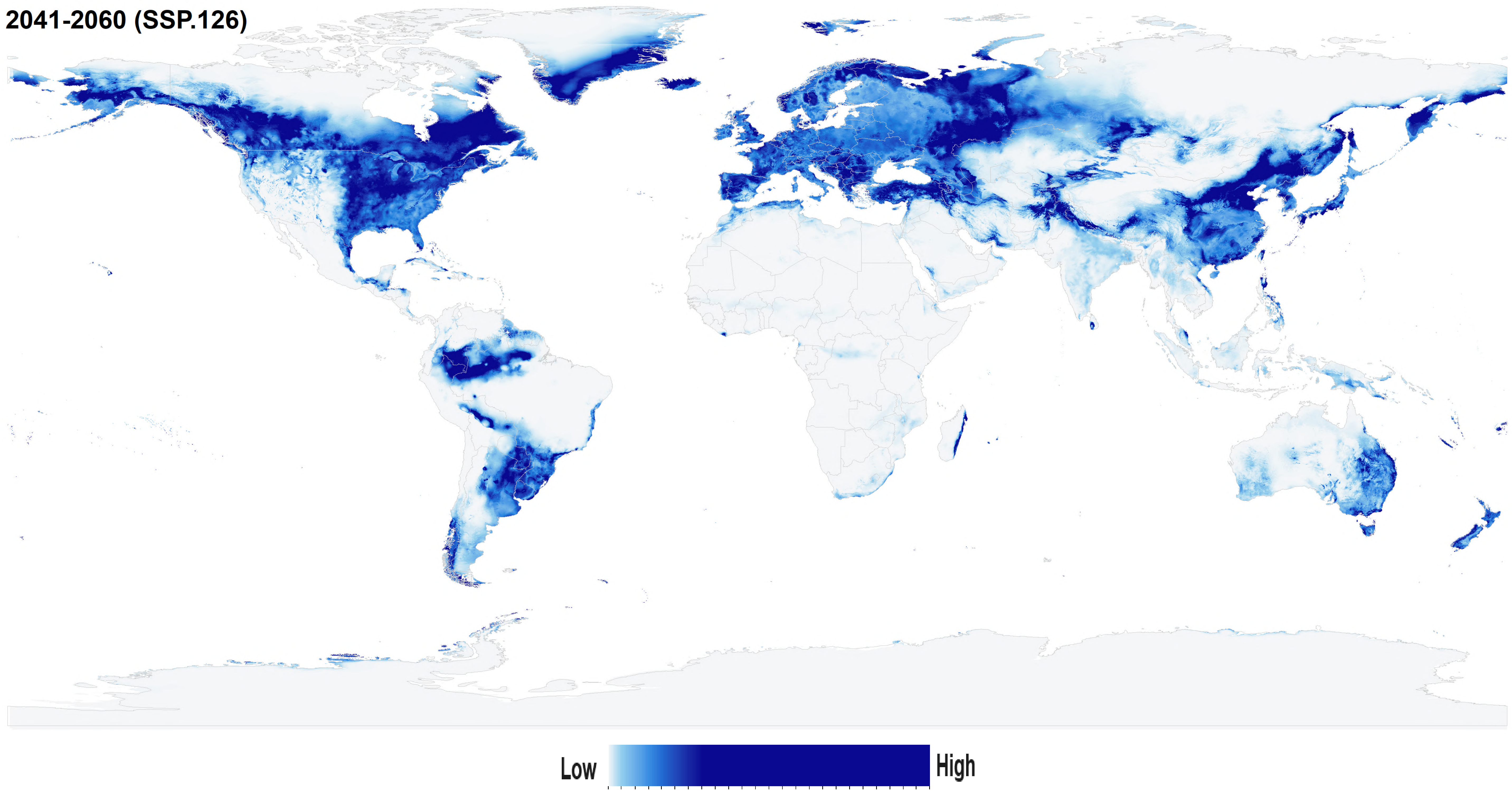
2041-2060 (SSP.126)
Low
High

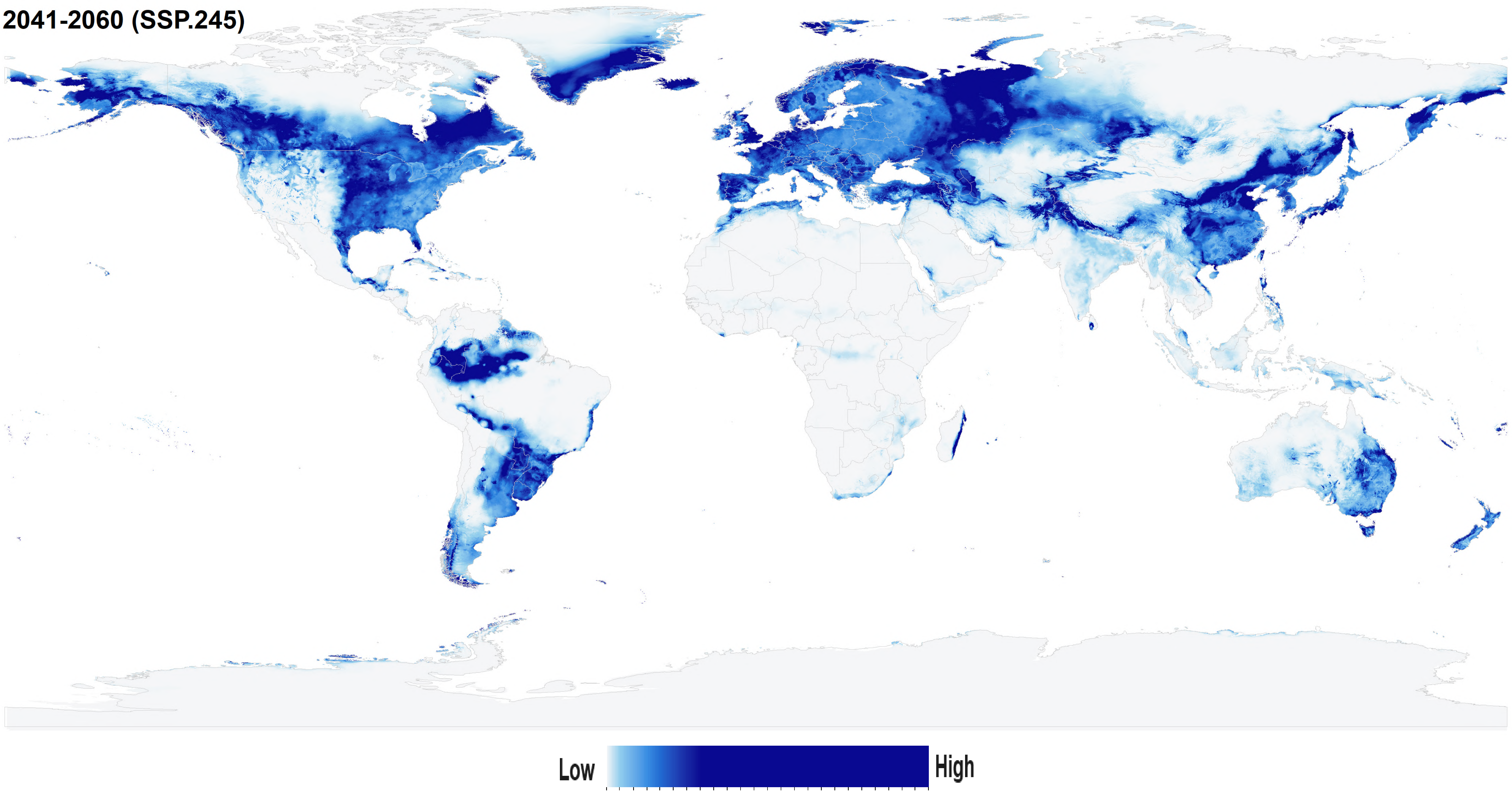
2041-2060 (SSP.245)
Low
High

2041-2060 (SSP.370)

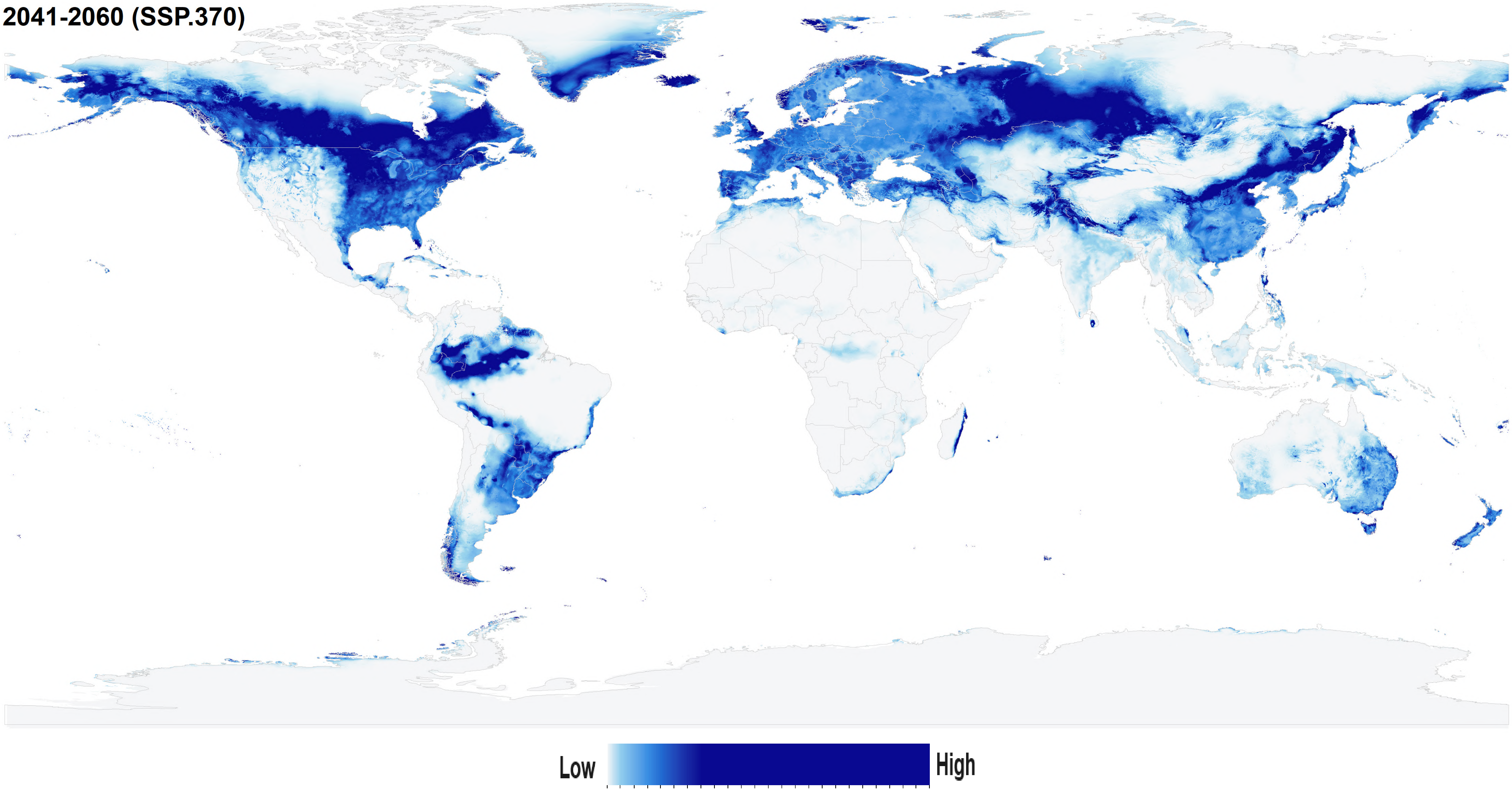

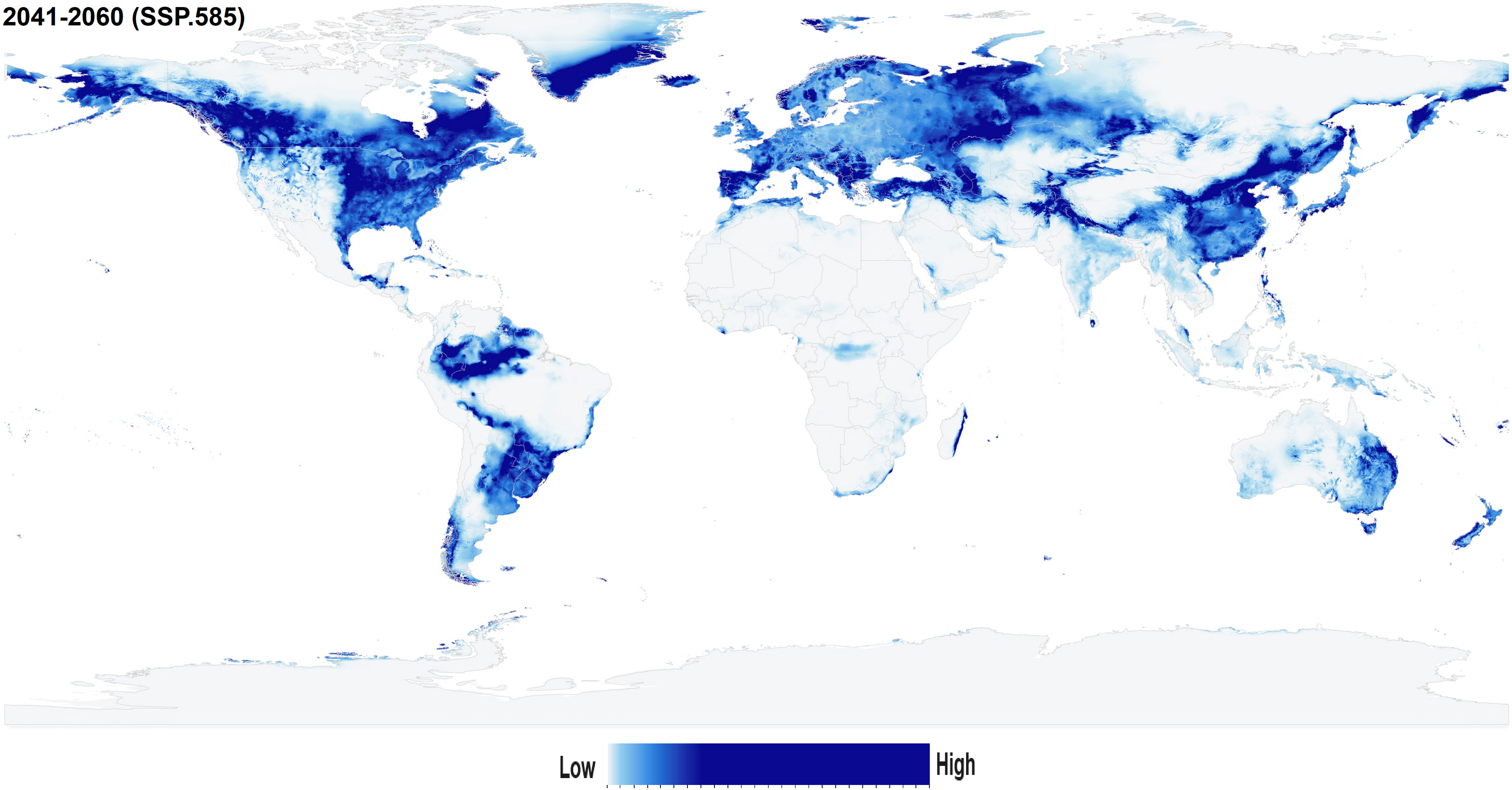
2041-2060 (SSP.585)
Low
High

2061-2080 (SSP.126)

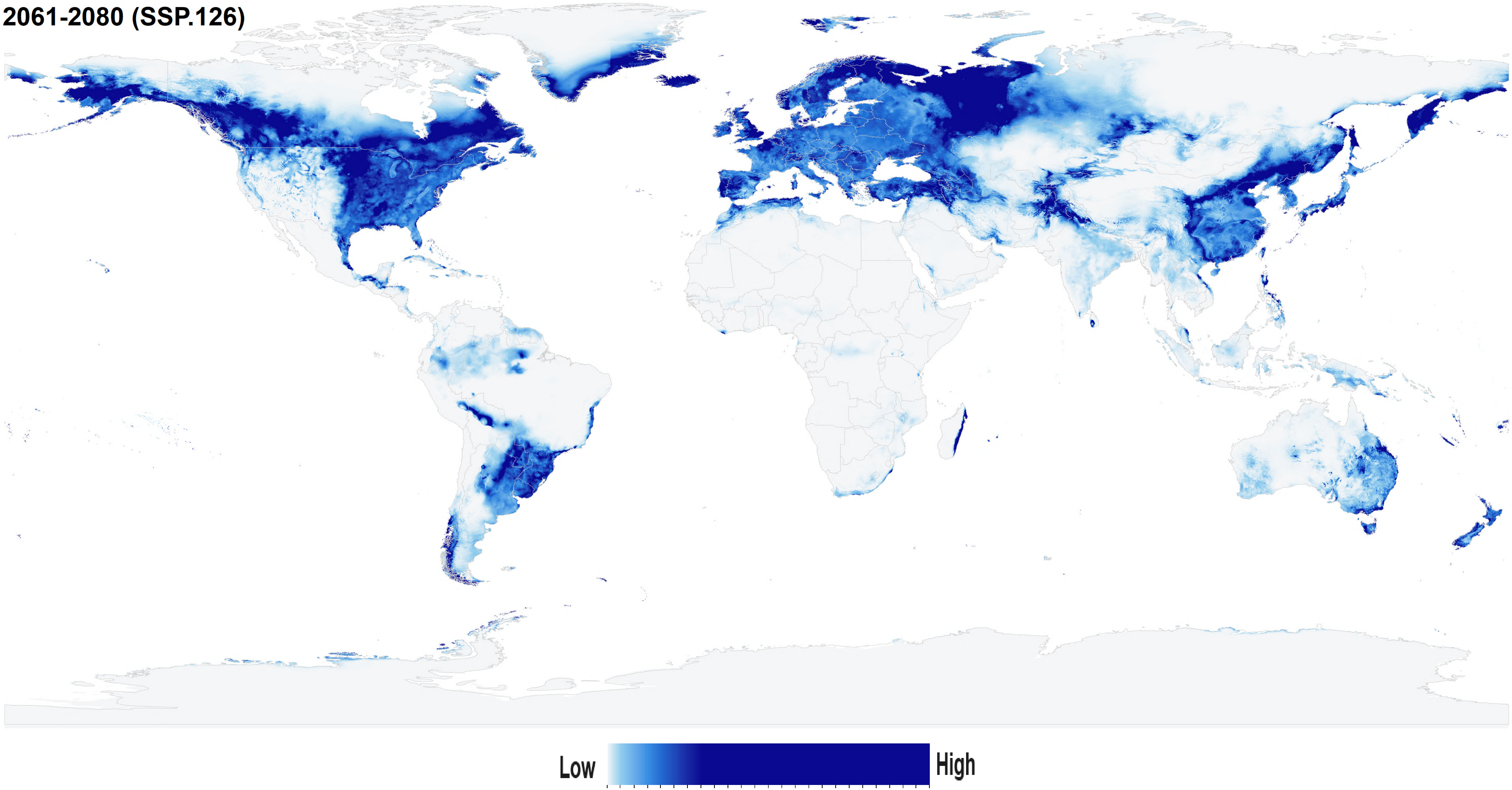

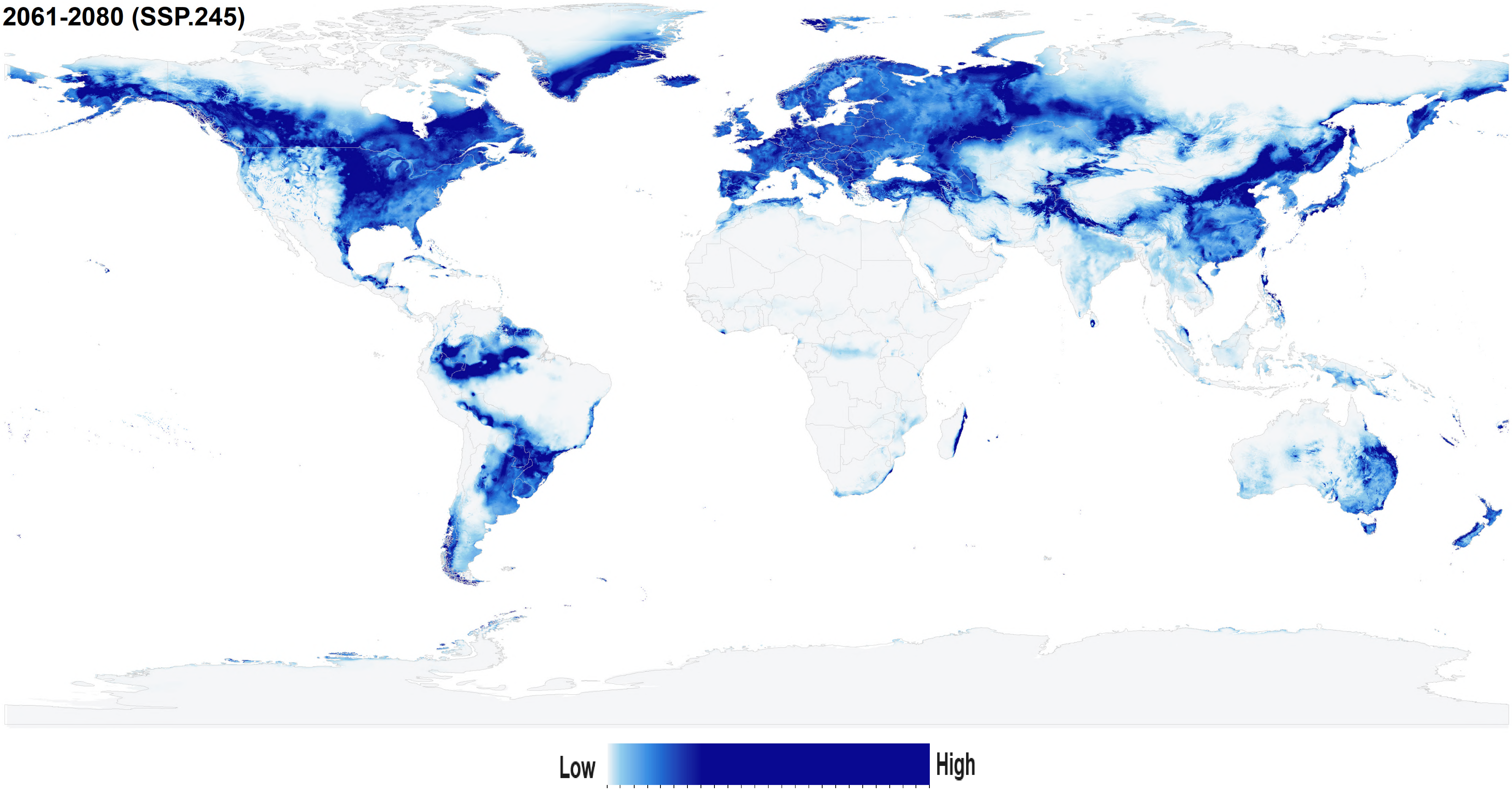
2061-2080 (SSP.245)
Low
High

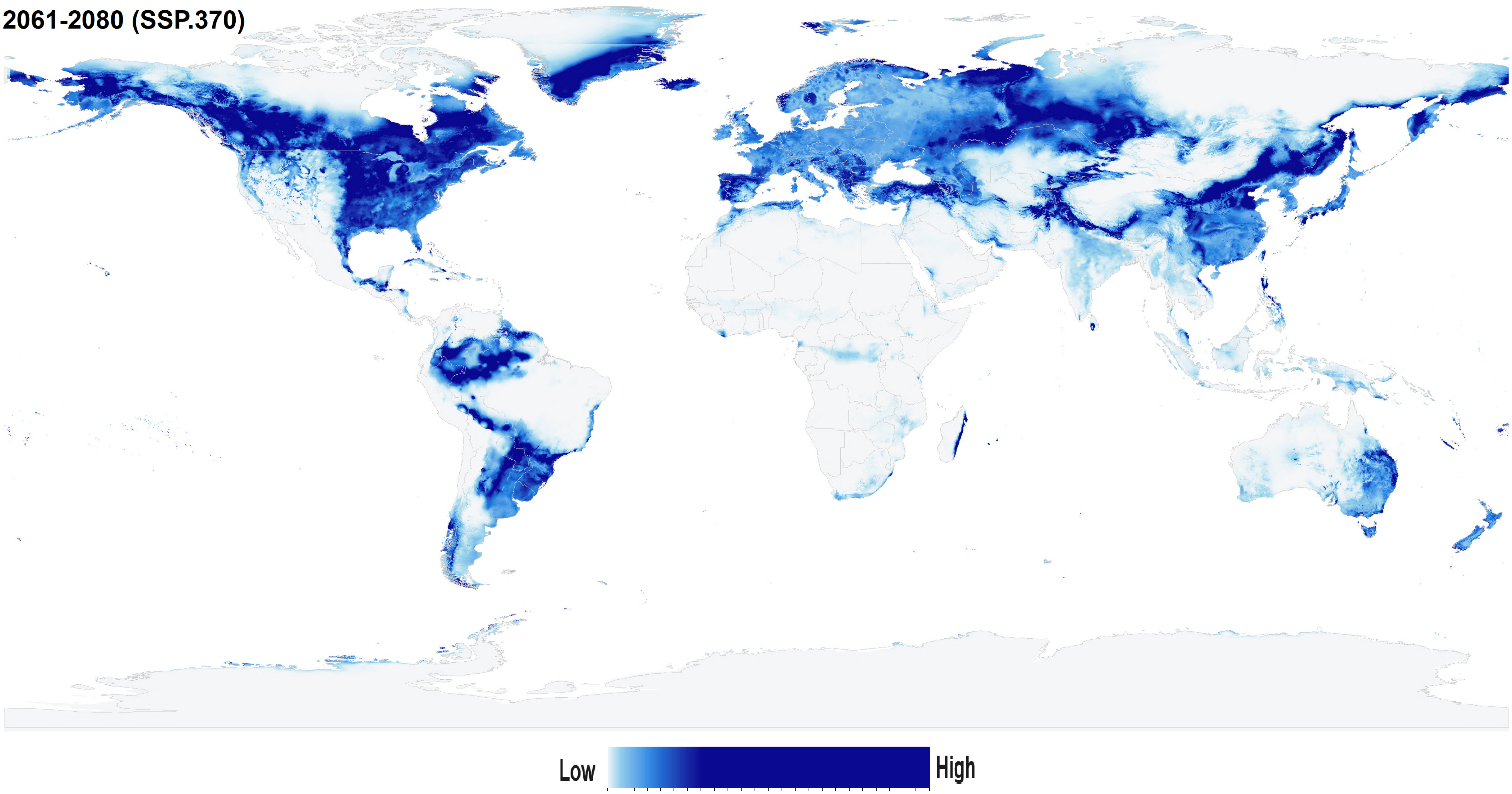
2061-2080 (SSP.370)
Low
High

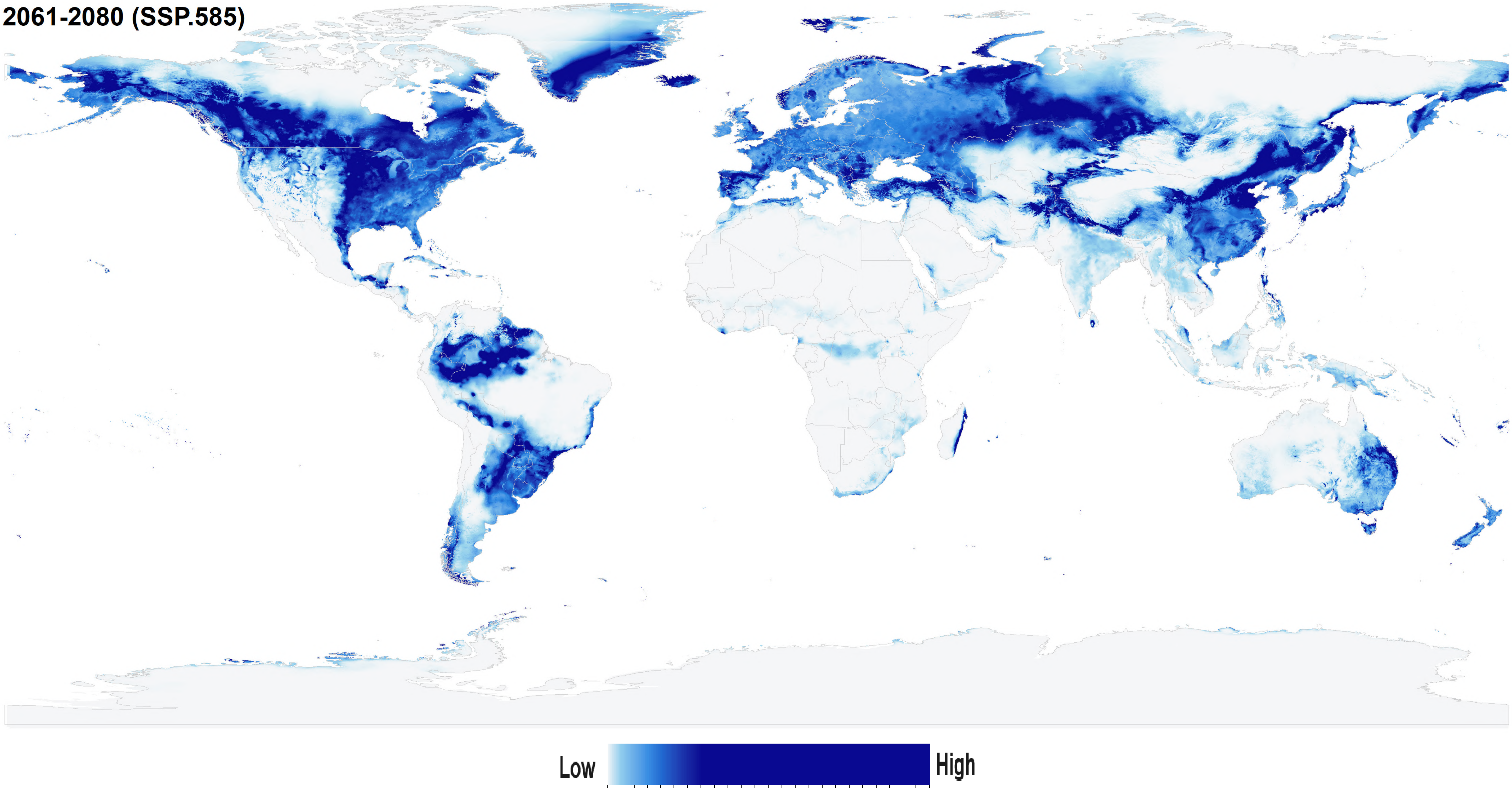
2061-2080 (SSP.585)
Low
High

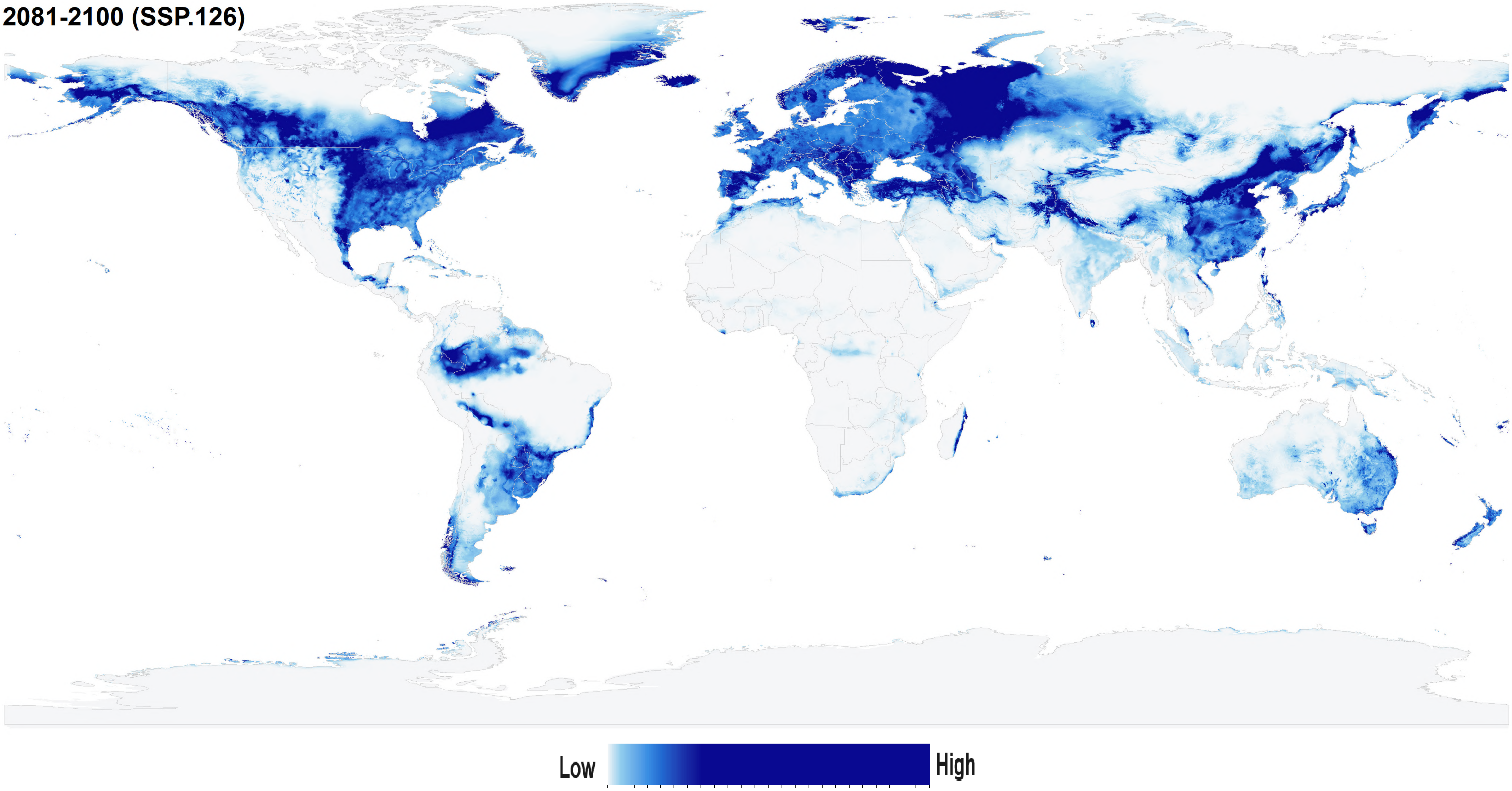
2081-2100 (SSP.126)
Low
High

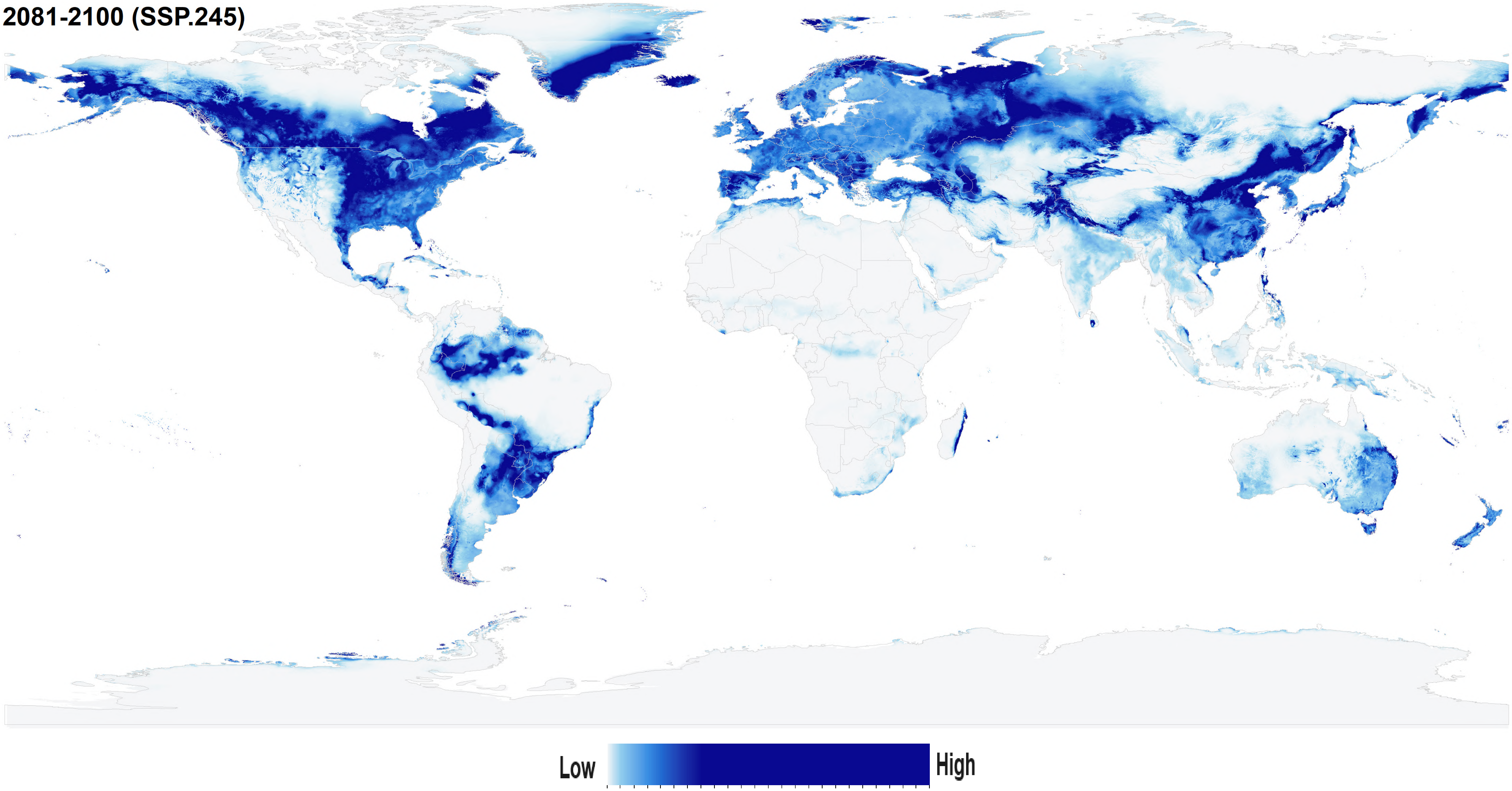
2081-2100 (SSP.245)
Low
High

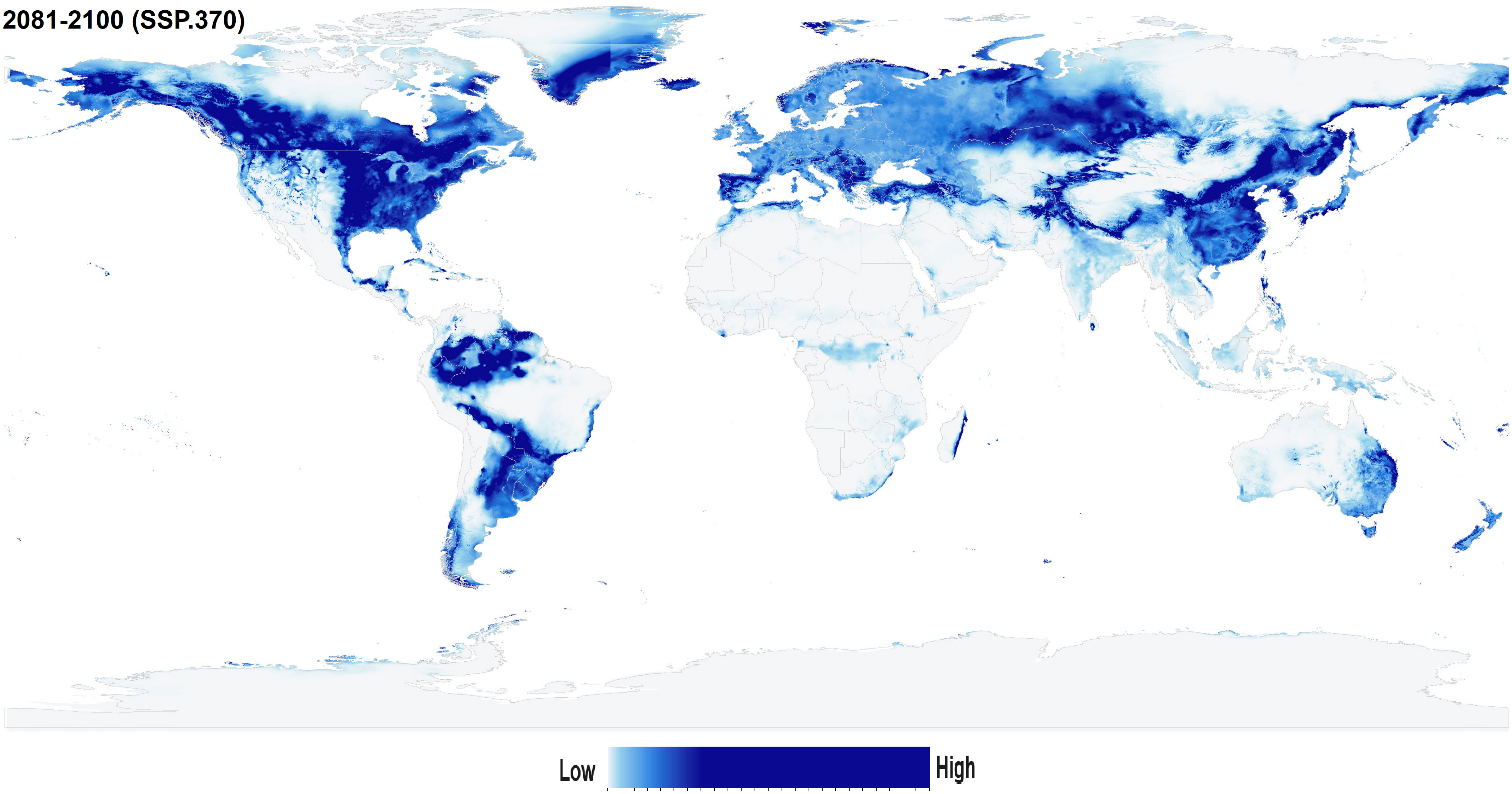
2081-2100 (SSP.370)
Low
High

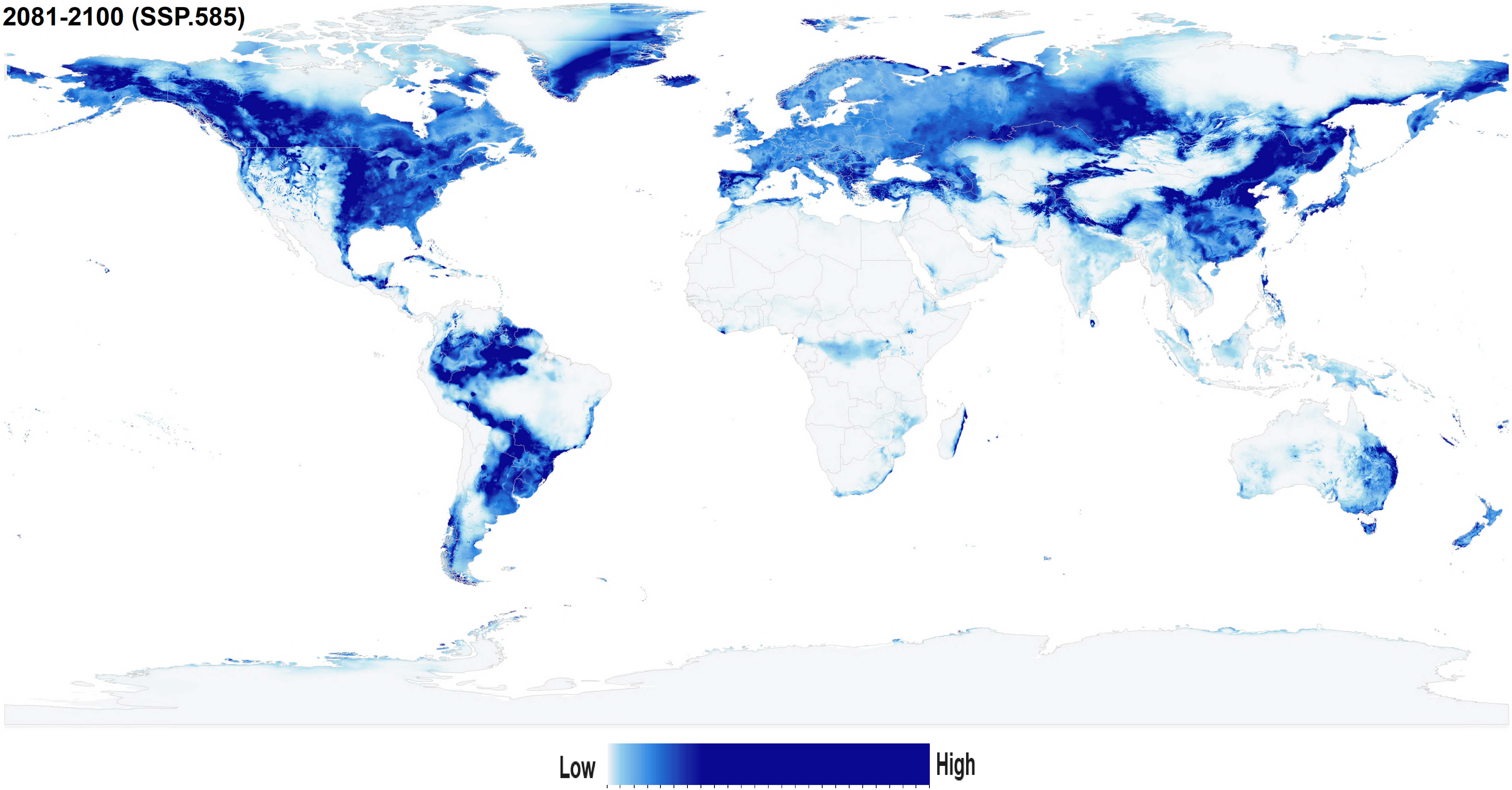
2081-2100 (SSP.585)
Low
High
