# Supplementary material for: Climate change influences on the potential geographic distribution of the invasive Asian longhorned tick, Haemaphysalis longicornis
Source: Sci Rep. 2025 Jan 17;15:2266. doi: 10.1038/s41598-025-86205-6 (PMC11748616; doi:10.1038/s41598-025-86205-6)
Supplement: Supplementary file 7 — Supplementary Material 7 [file 41598_2025_86205_MOESM7_ESM.pdf]

S. File 7: This file presents probability maps (A) and corresponding standard deviation (STD) maps (B) depicting the environmental suitability for the tick species *Haemaphysalis longicornis* under various climate change scenarios across four different time periods. The maps are based on four Shared Socioeconomic Pathways (SSPs), which include SSP.126, SSP.245, SSP.370, and SSP.585. The probability maps indicate the likelihood of environmental suitability for *H. longicornis*, while the STD maps provide a measure of uncertainty or variability in the model predictions. These visualizations help assess both the potential distribution of the species and the confidence in these projections under evolving climatic conditions.

(A)

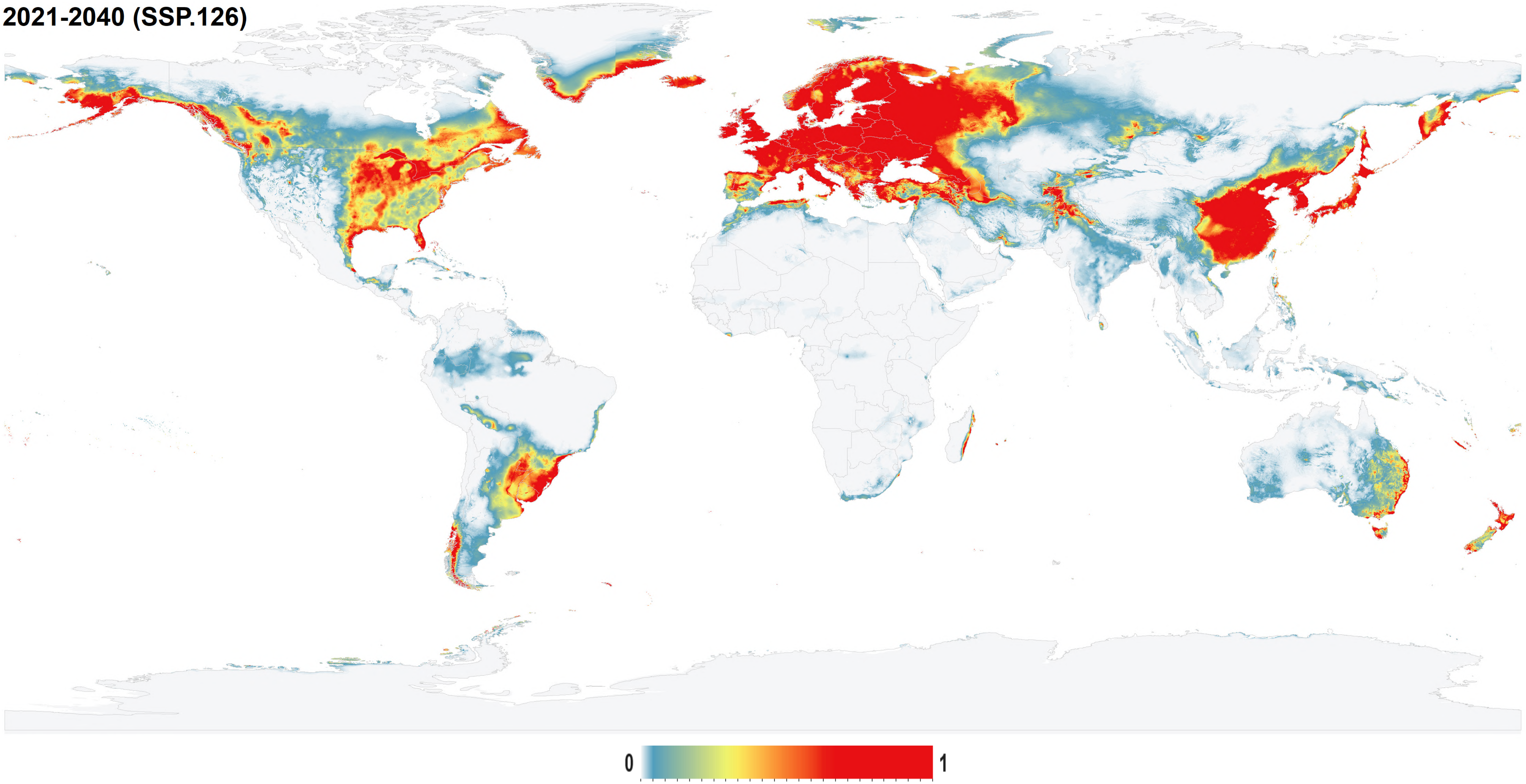

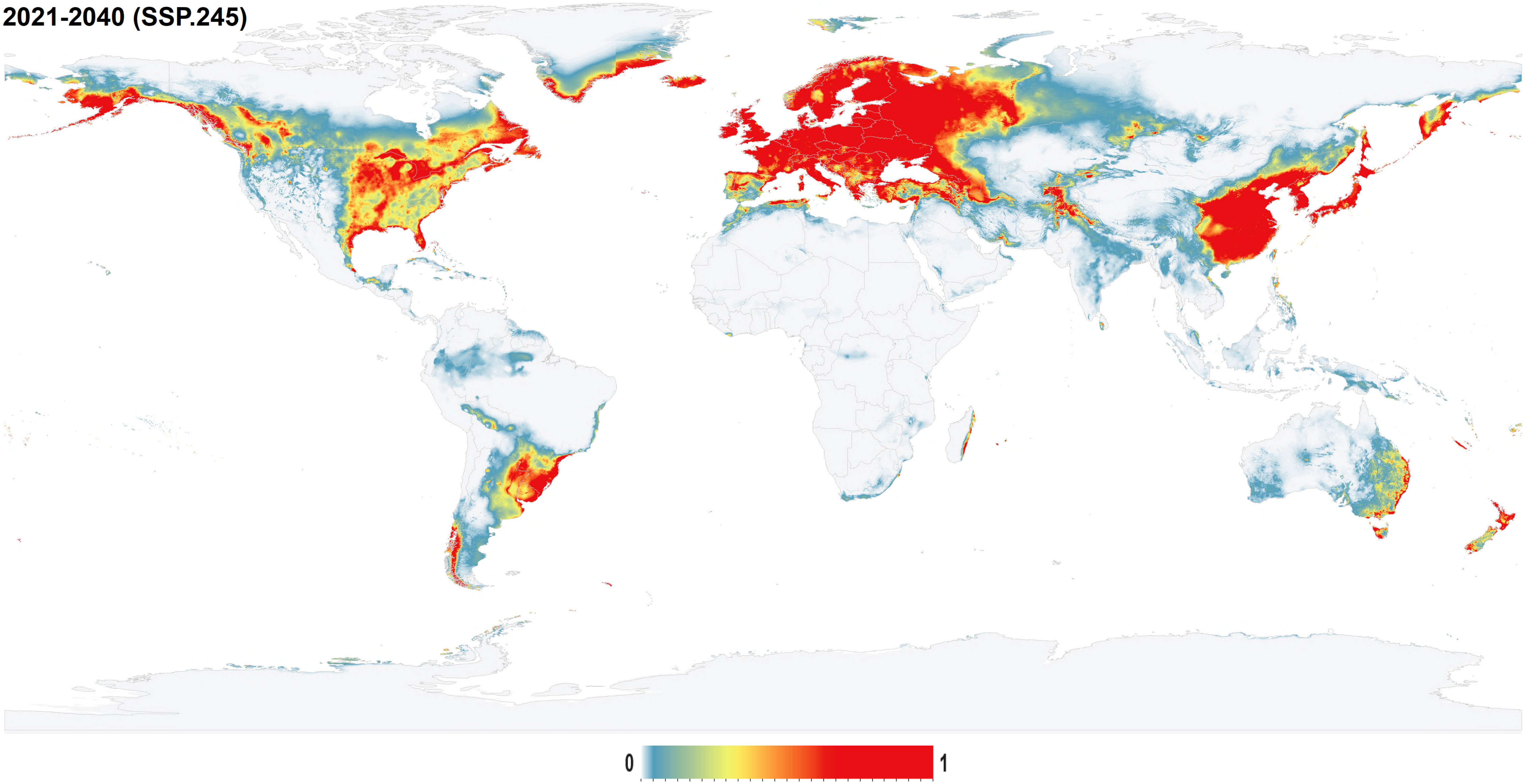
2021-2040 (SSP.245)
0
1

2021-2040 (SSP.370)

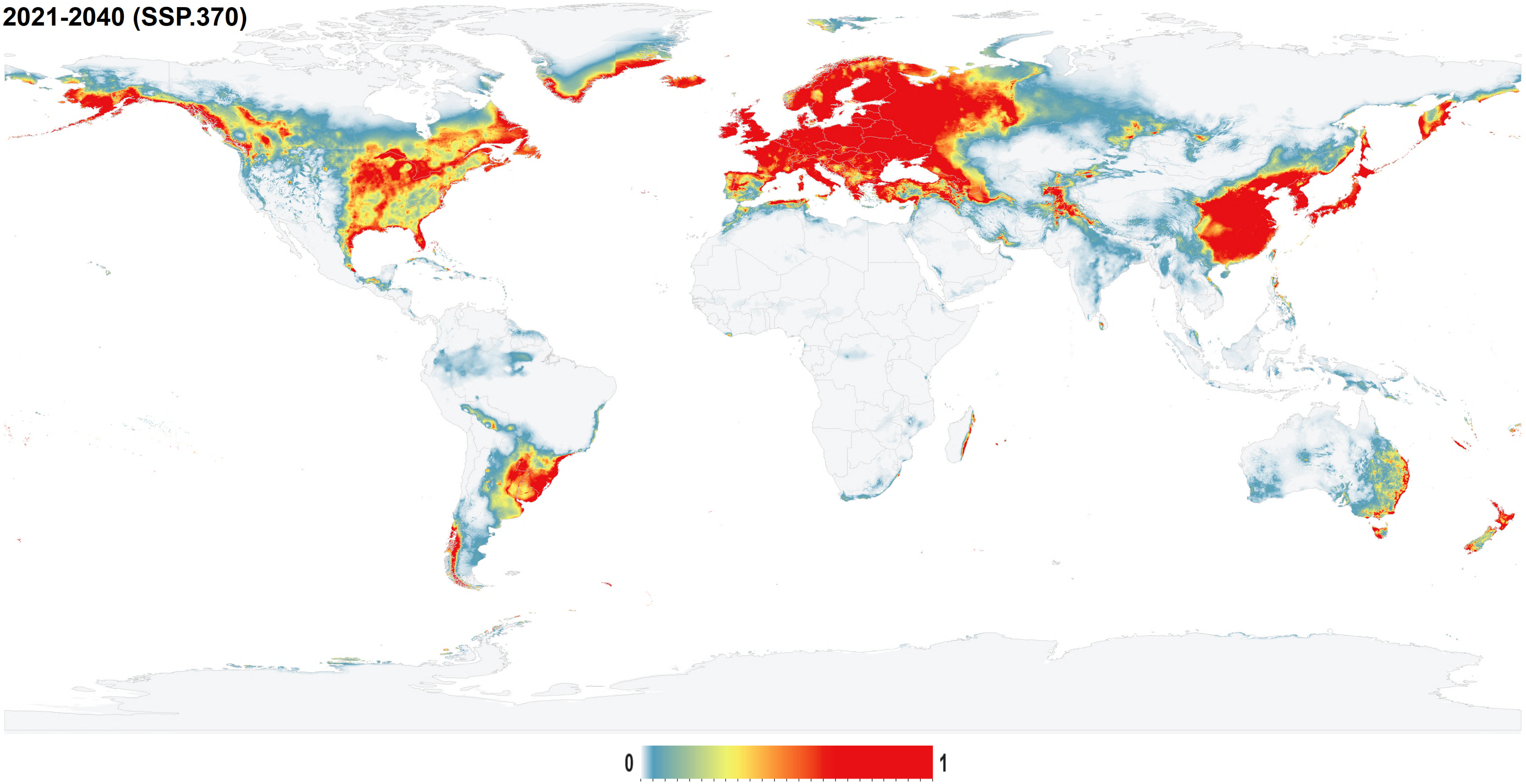

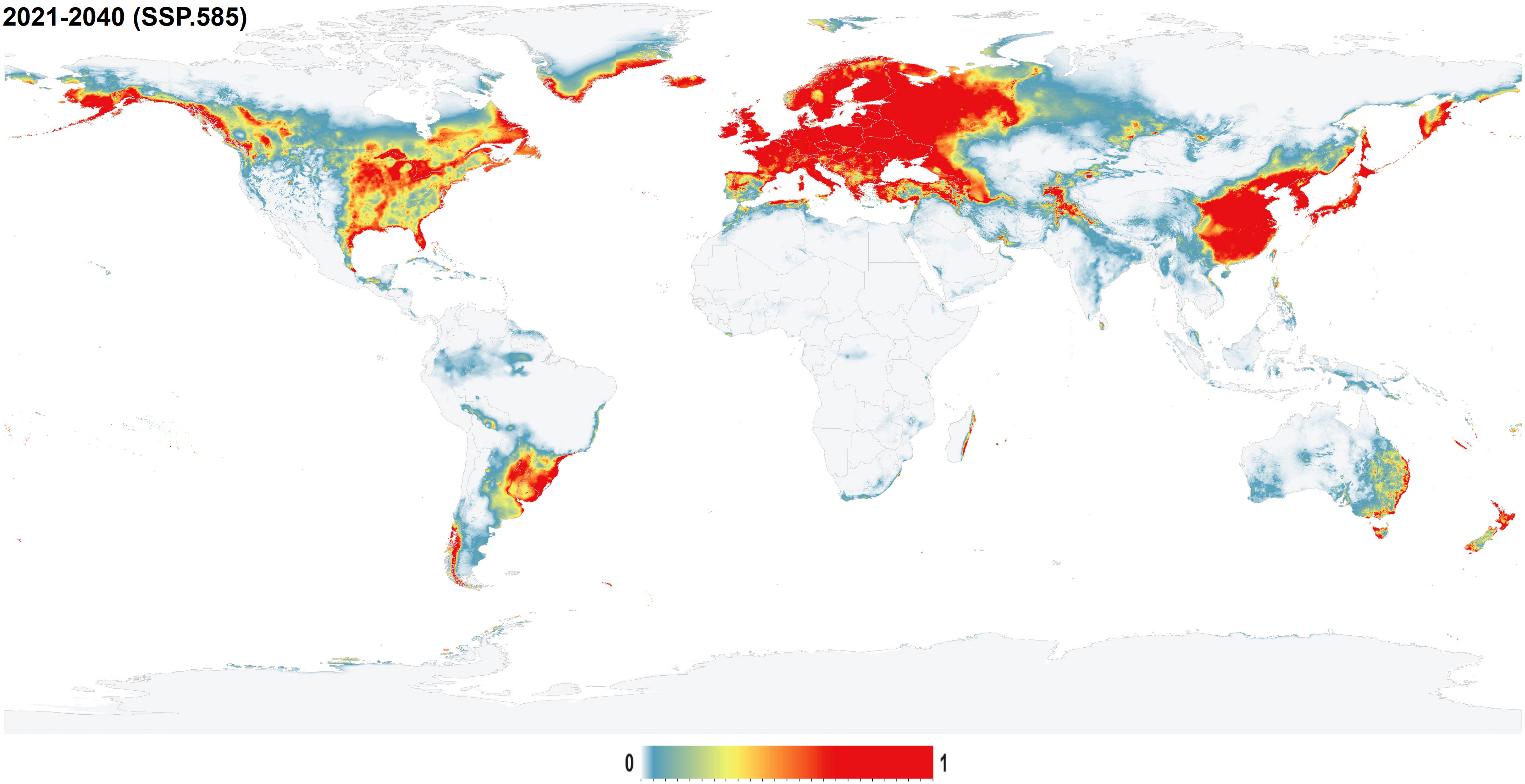
2021-2040 (SSP.585)
0
1

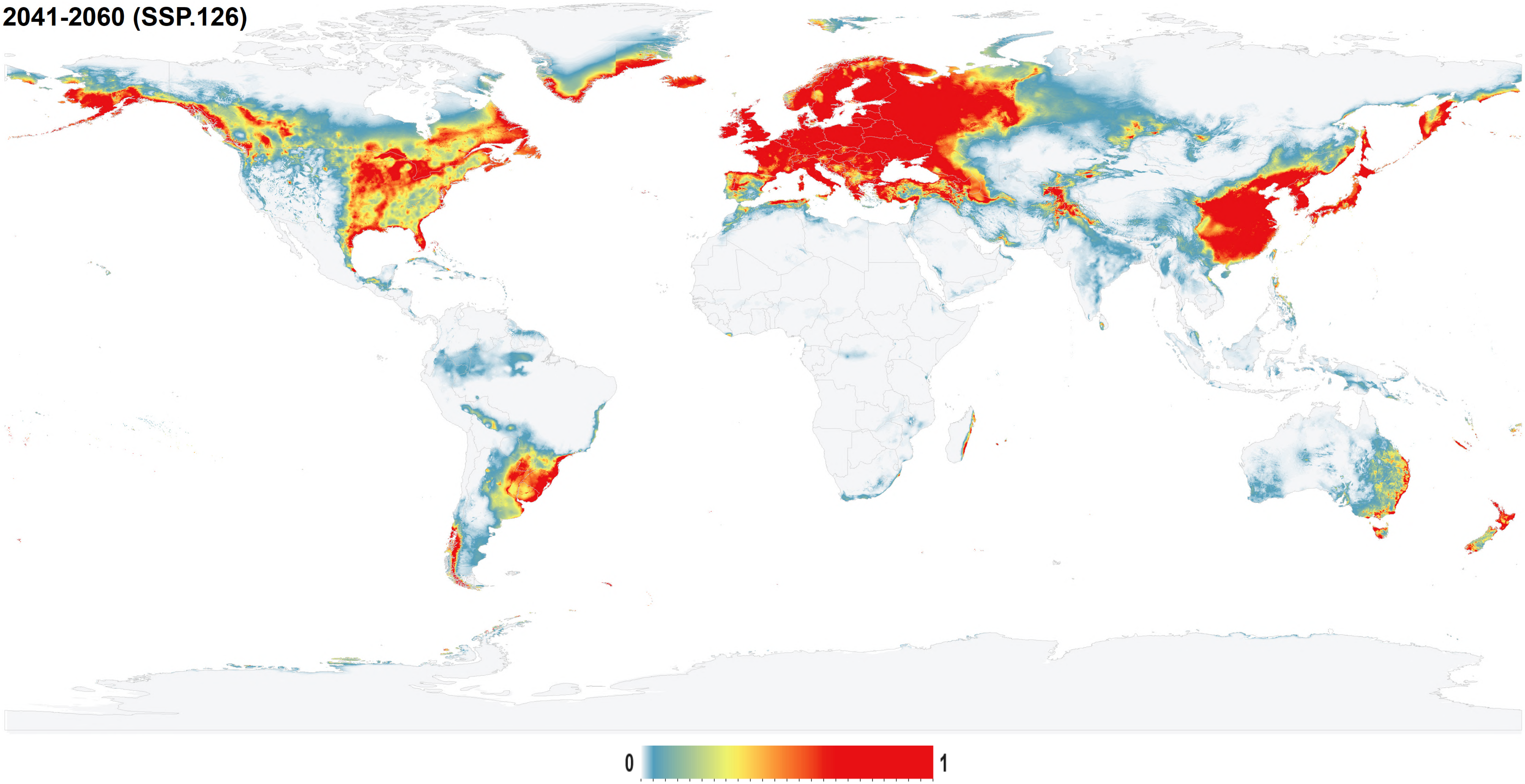
2041-2060 (SSP.126)
0
1

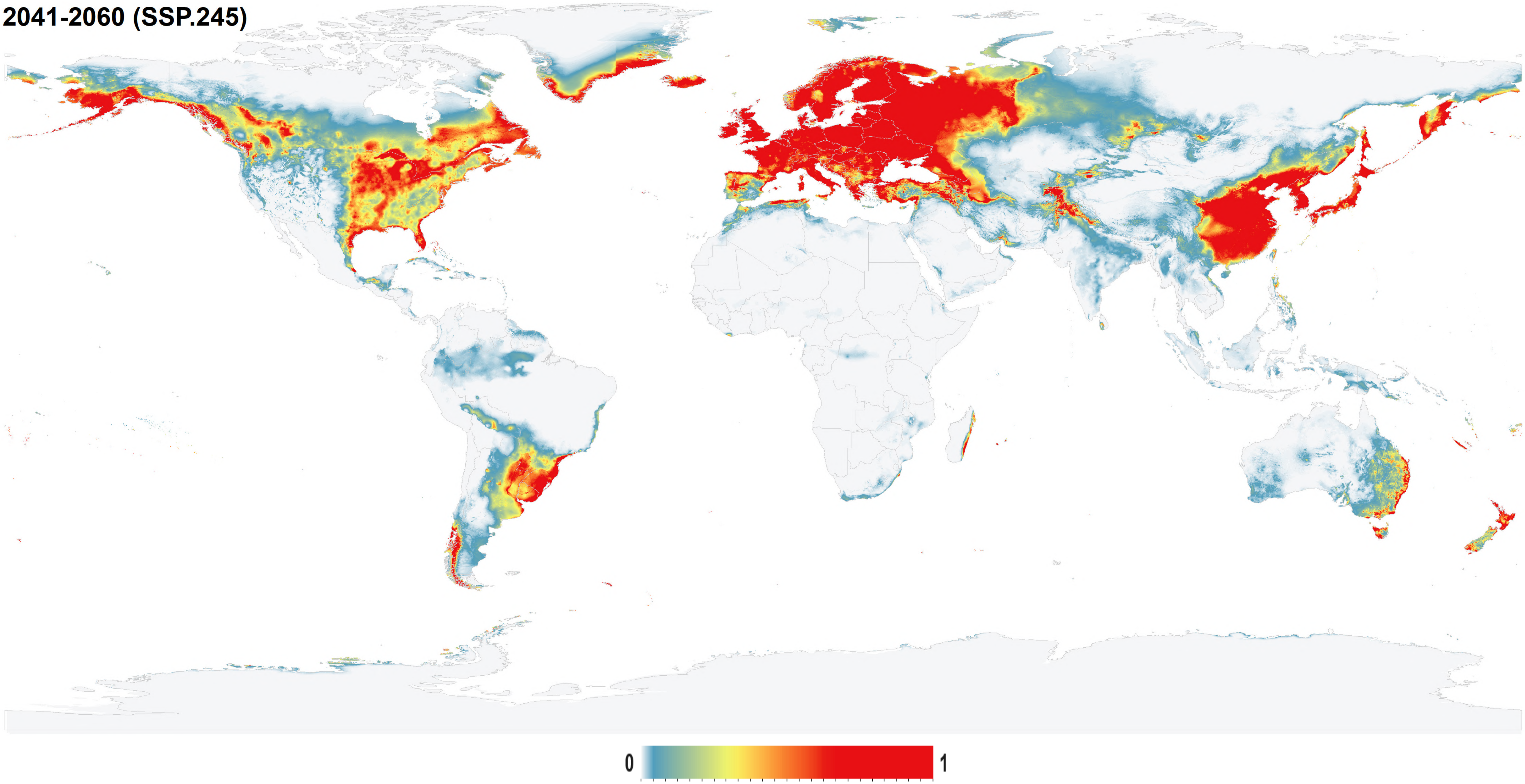
2041-2060 (SSP.245)
0
1

2041-2060 (SSP.370)

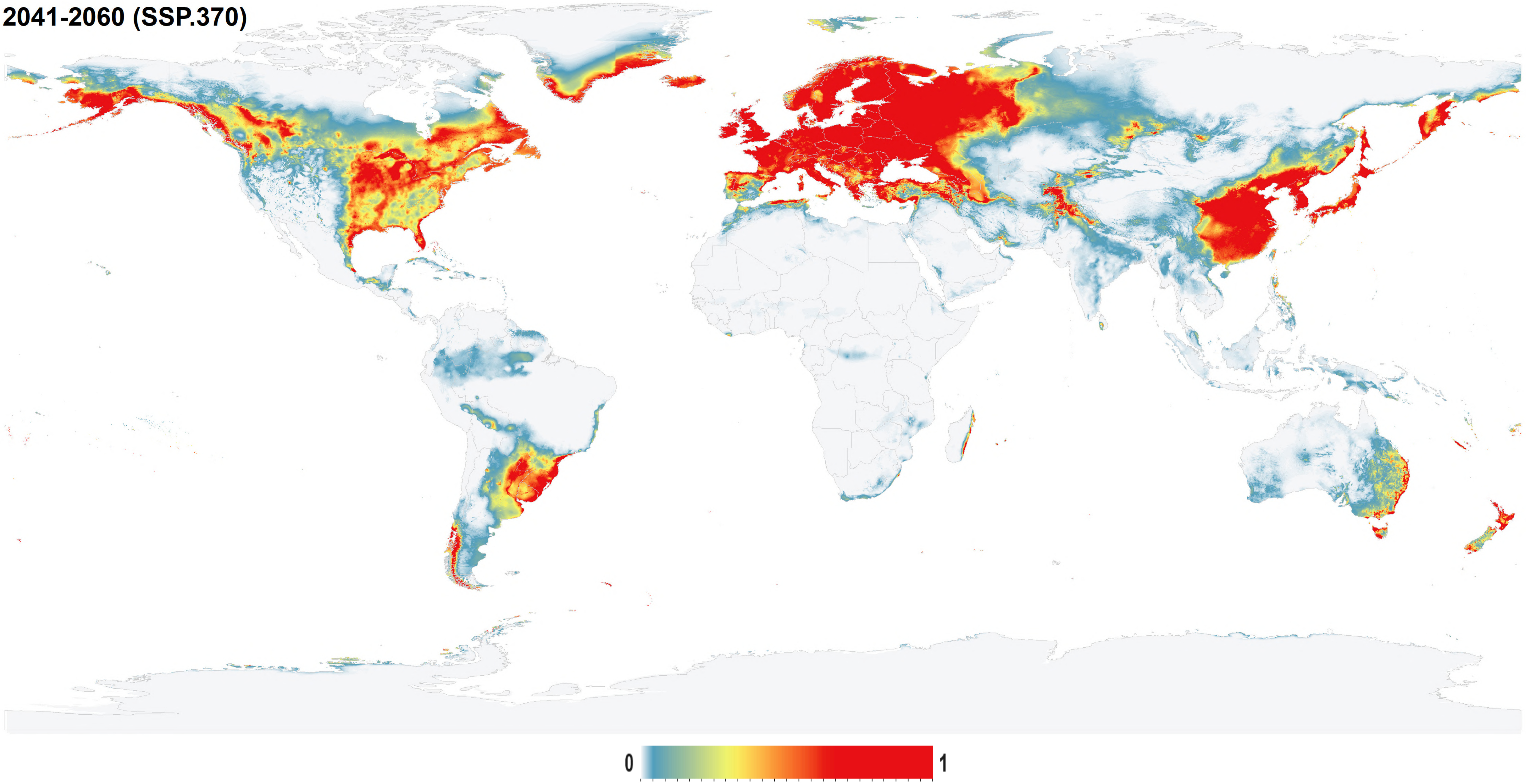

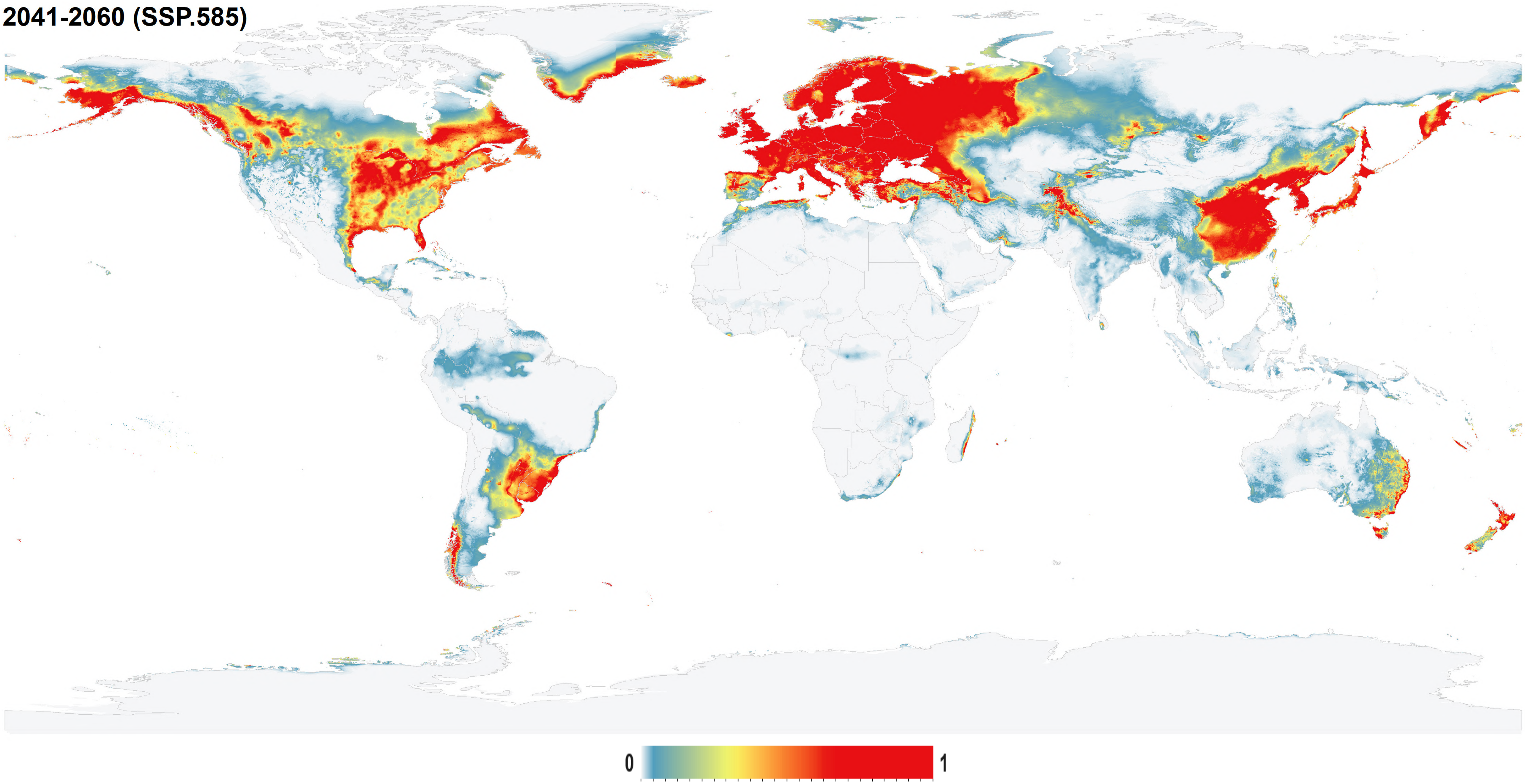
2041-2060 (SSP.585)
0
1

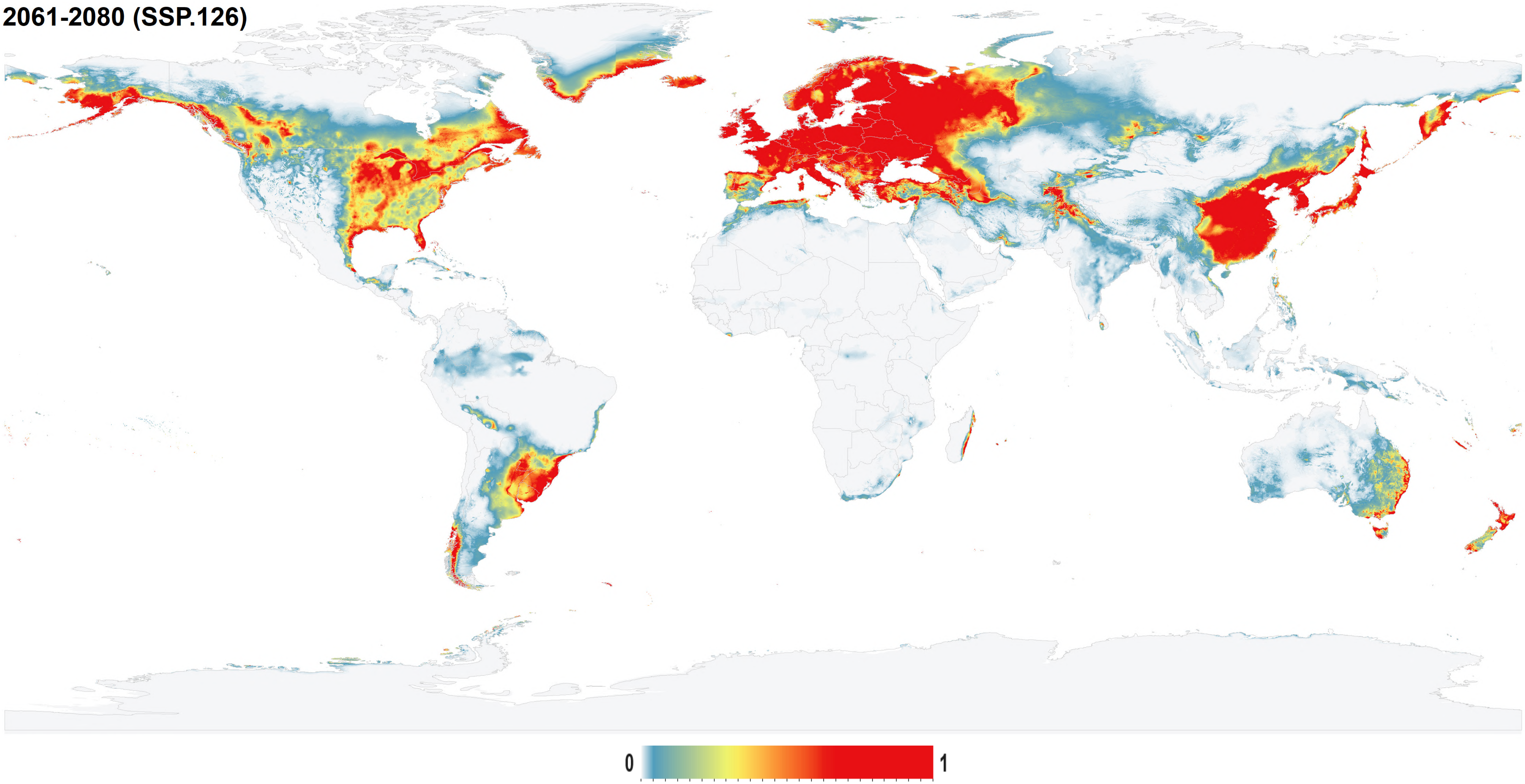
2061-2080 (SSP.126)
0
1

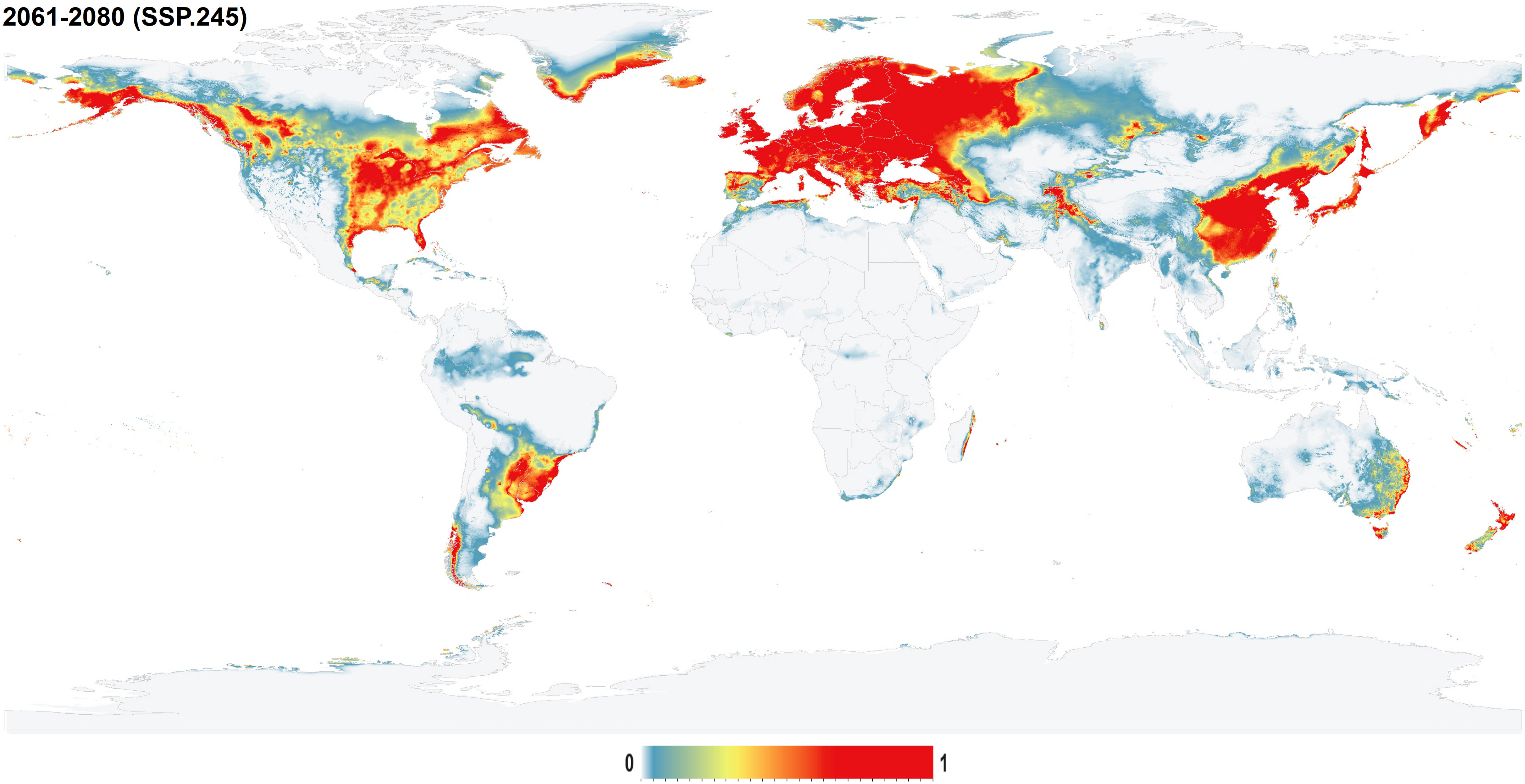
2061-2080 (SSP.245)
0
1

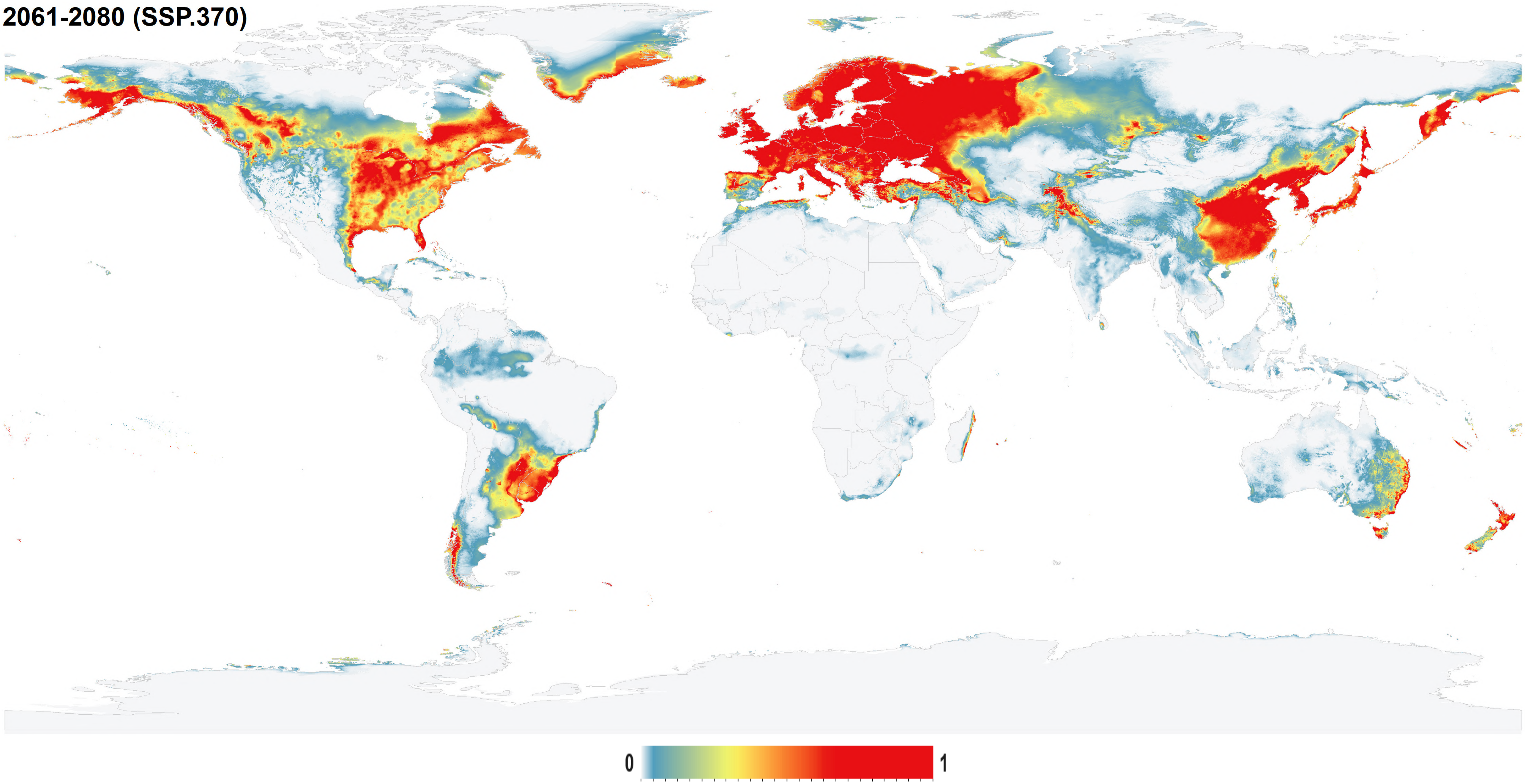
2061-2080 (SSP.370)
0
1

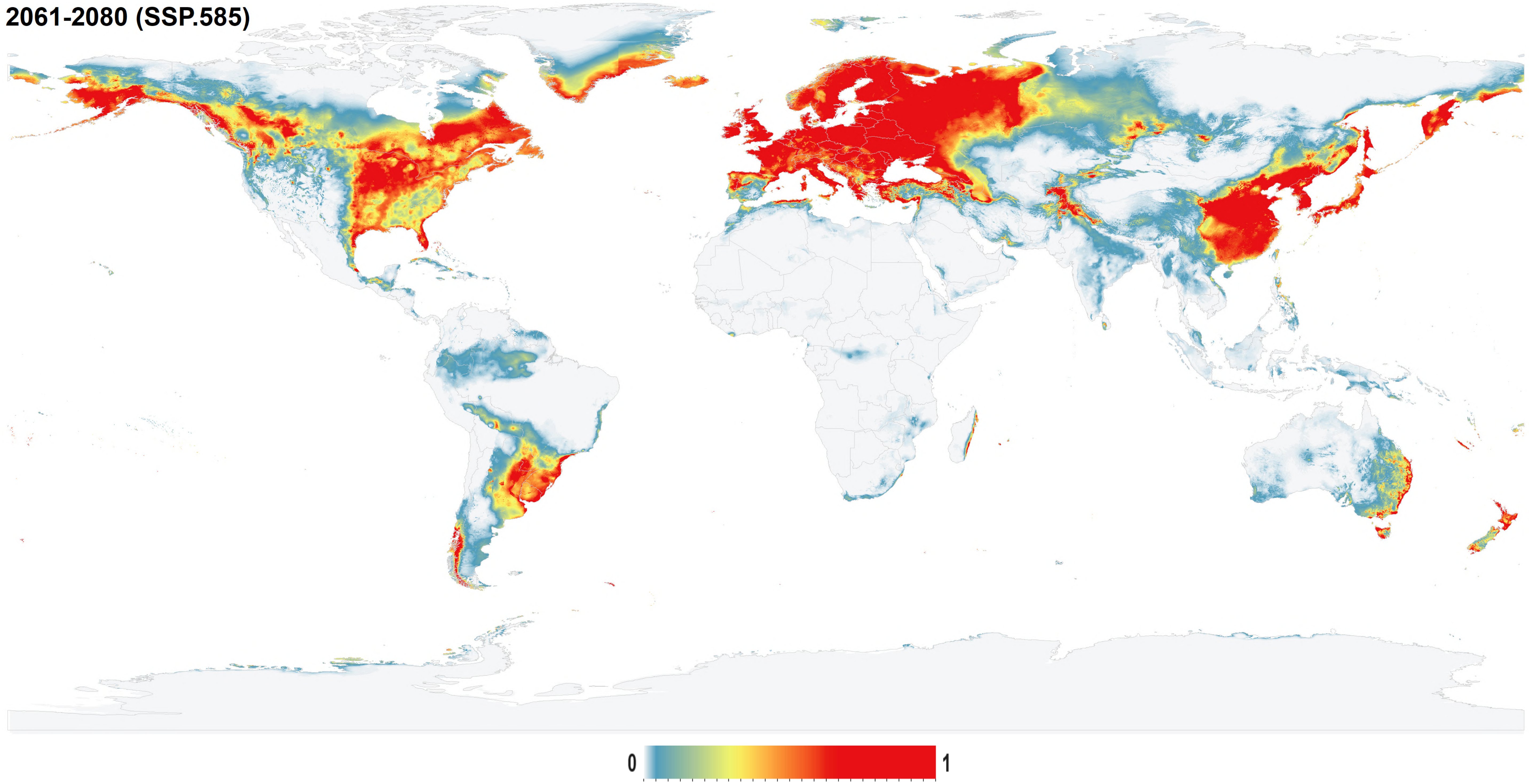
2061-2080 (SSP.585)
0
1

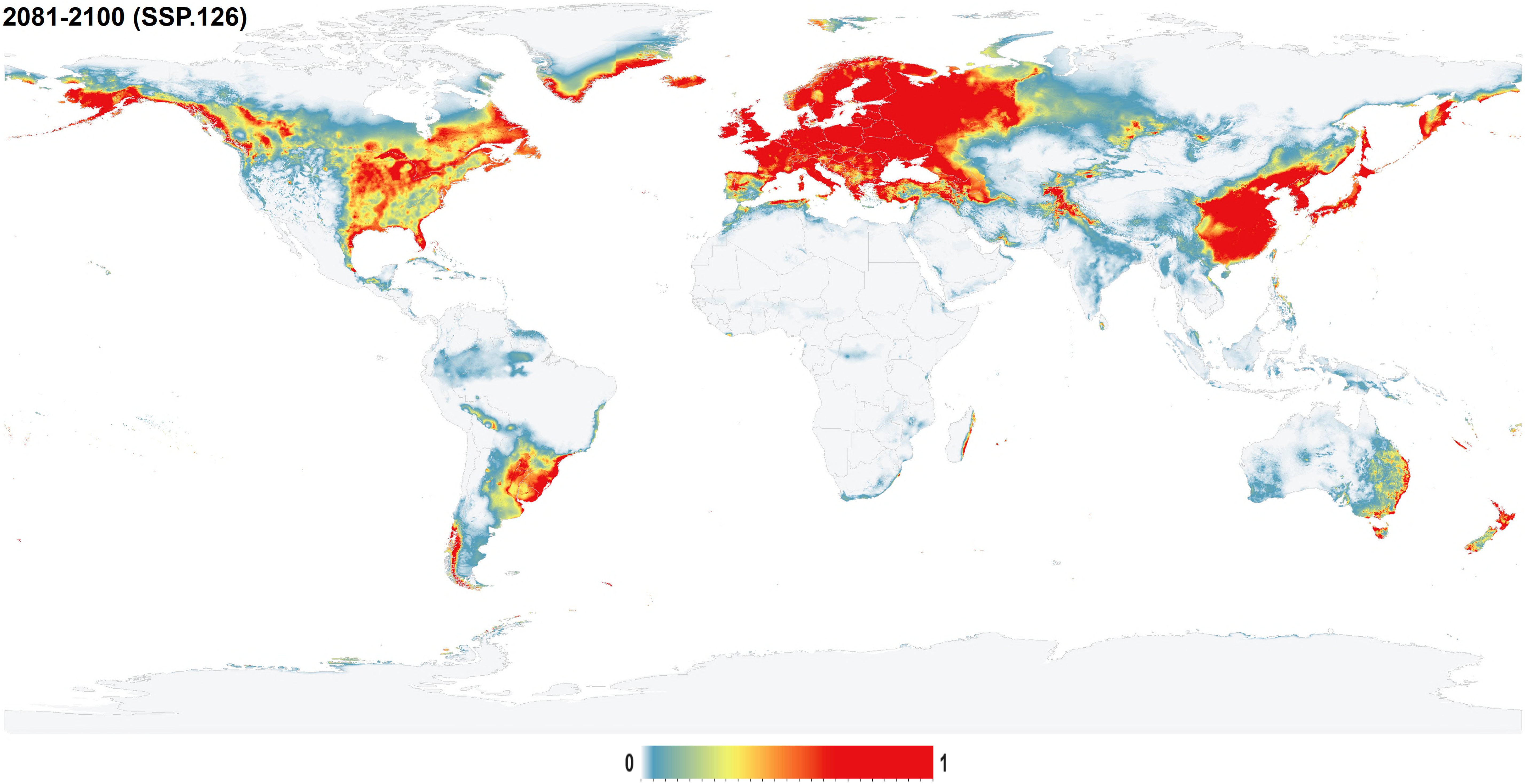
2081-2100 (SSP.126)
0
1

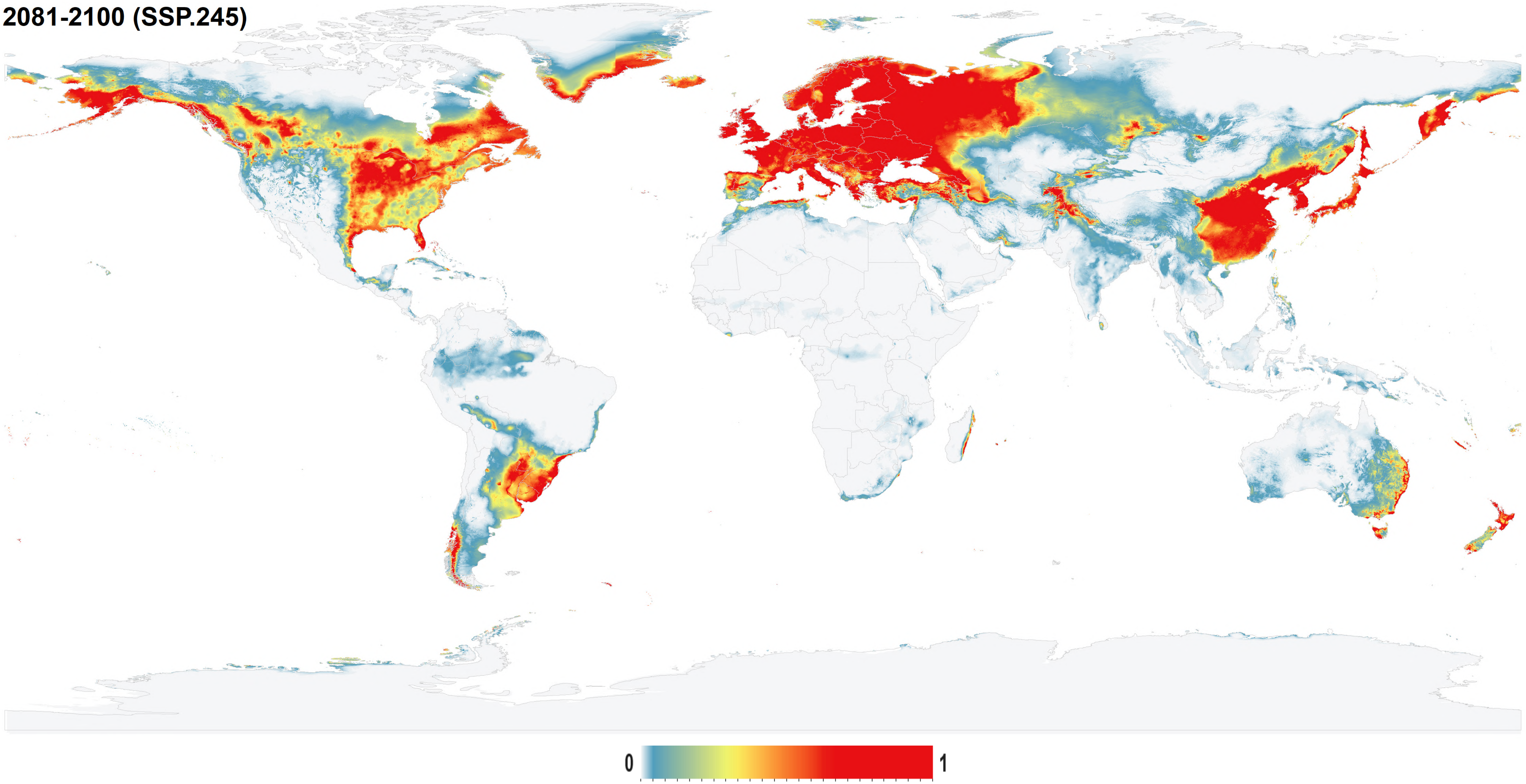
2081-2100 (SSP.245)
0
1

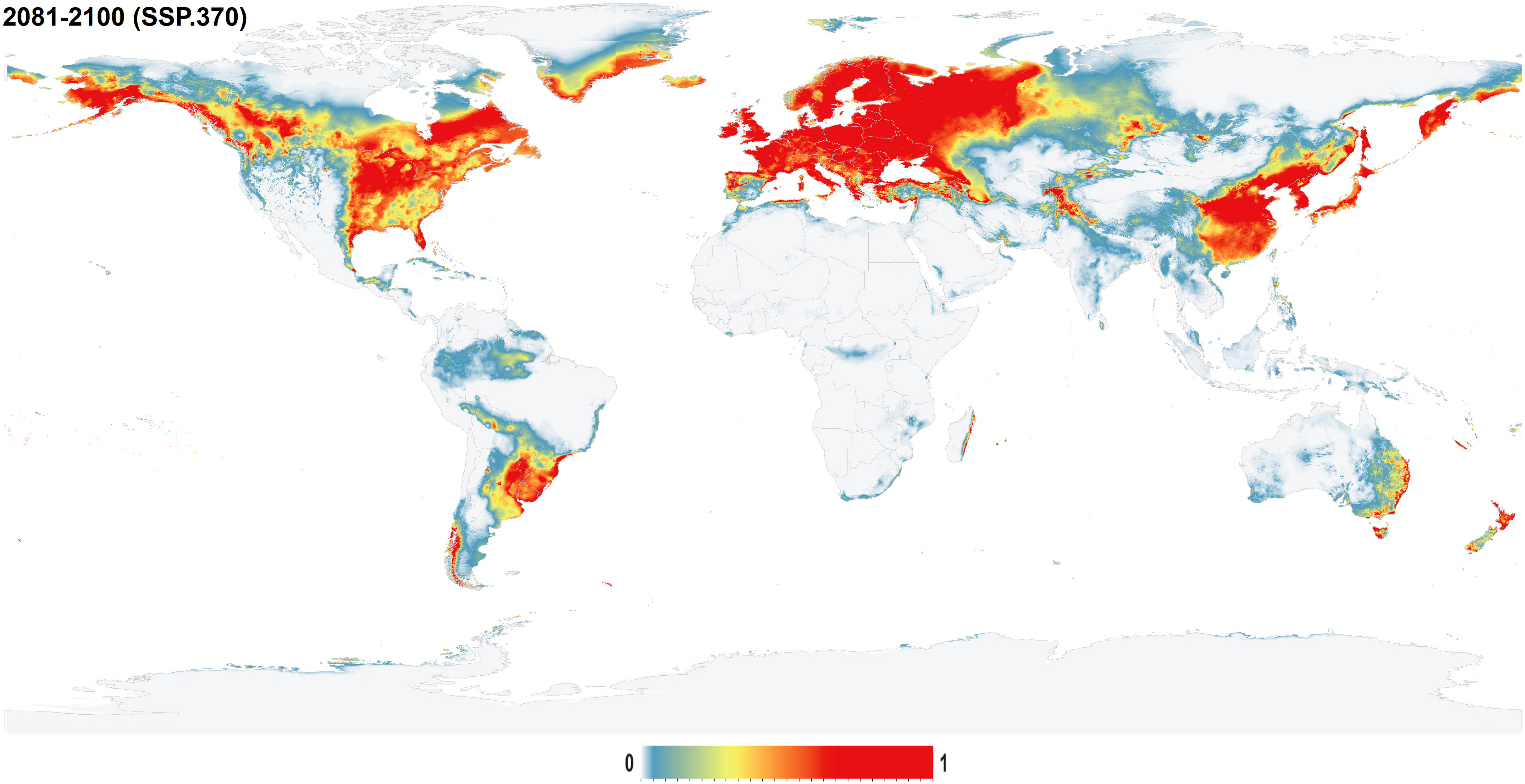
2081-2100 (SSP.370)
0
1

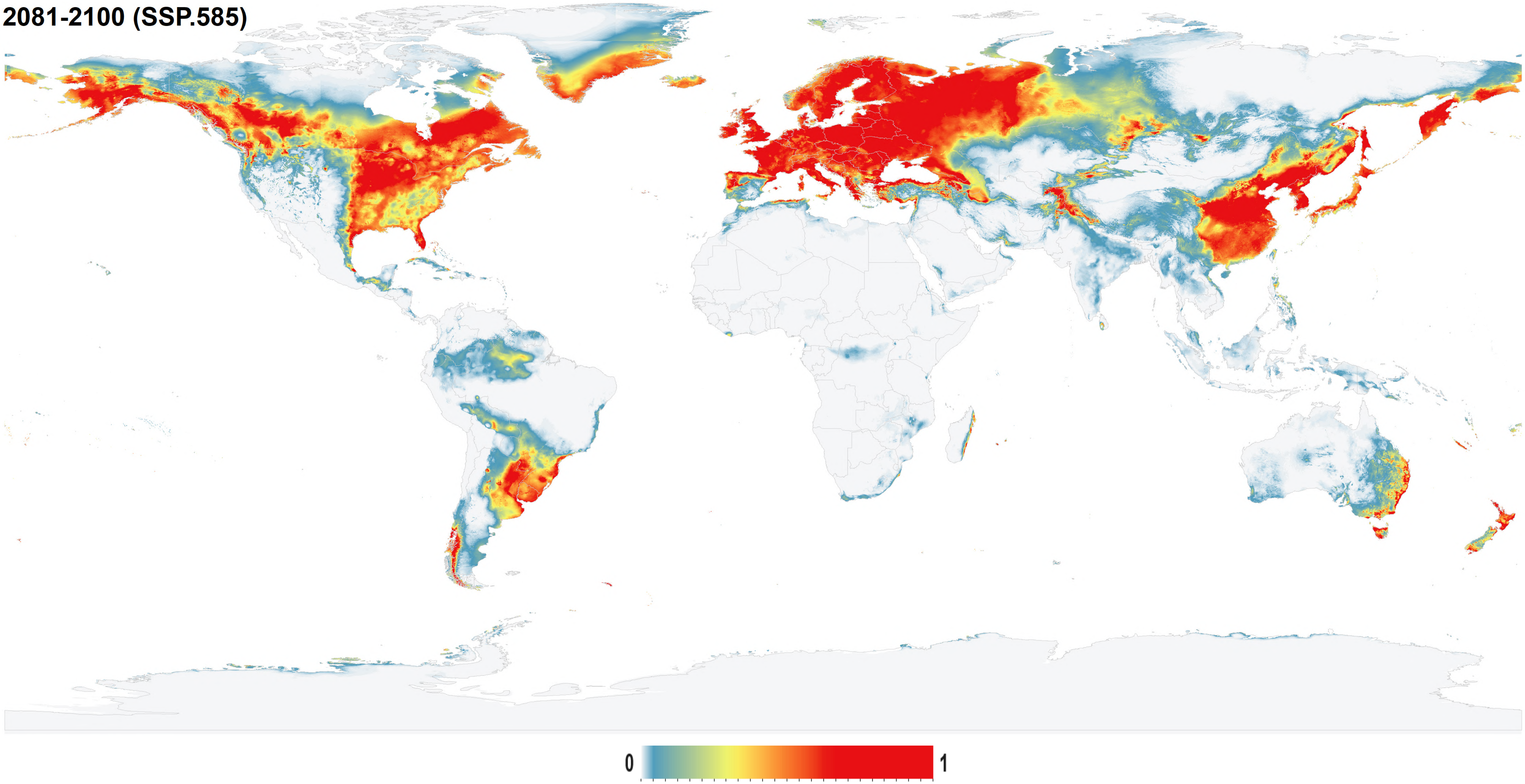
2081-2100 (SSP.585)
0
1

(B)

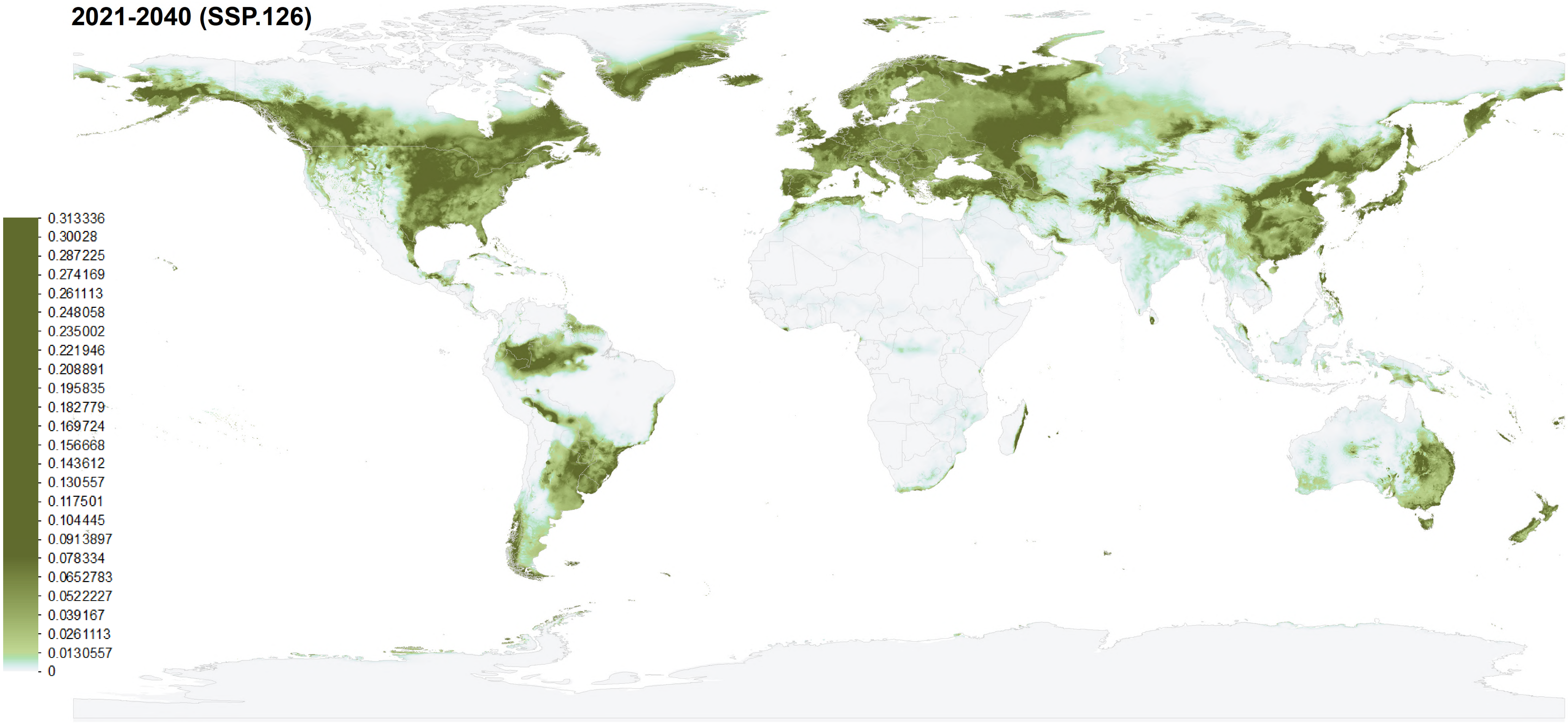
2021-2040 (SSP.126)
0.313336
0.30028
0.287225
0.274169
0.261113
0.248058
0.235002
0.221946
0.208891
0.195835
0.182779
0.169724
0.156668
0.143612
0.130557
0.117501
0.104445
0.0913897
0.078334
0.0652783
0.0522227
0.039167
0.0261113
0.0130557
0

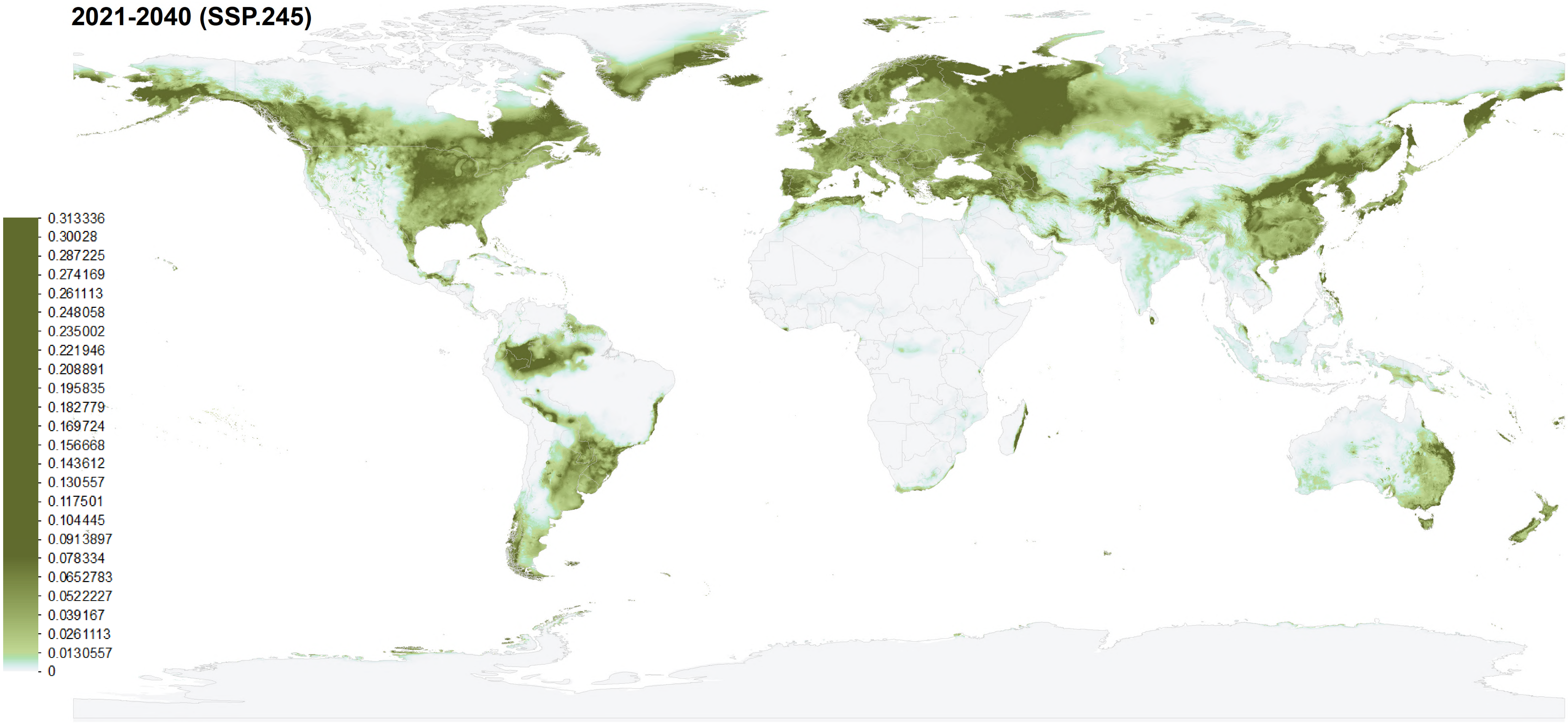
2021-2040 (SSP.245)
0.313336
0.30028
0.287225
0.274169
0.261113
0.248058
0.235002
0.221946
0.208891
0.195835
0.182779
0.169724
0.156668
0.143612
0.130557
0.117501
0.104445
0.0913897
0.078334
0.0652783
0.0522227
0.039167
0.0261113
0.0130557
0

2021-2040 (SSP.370)

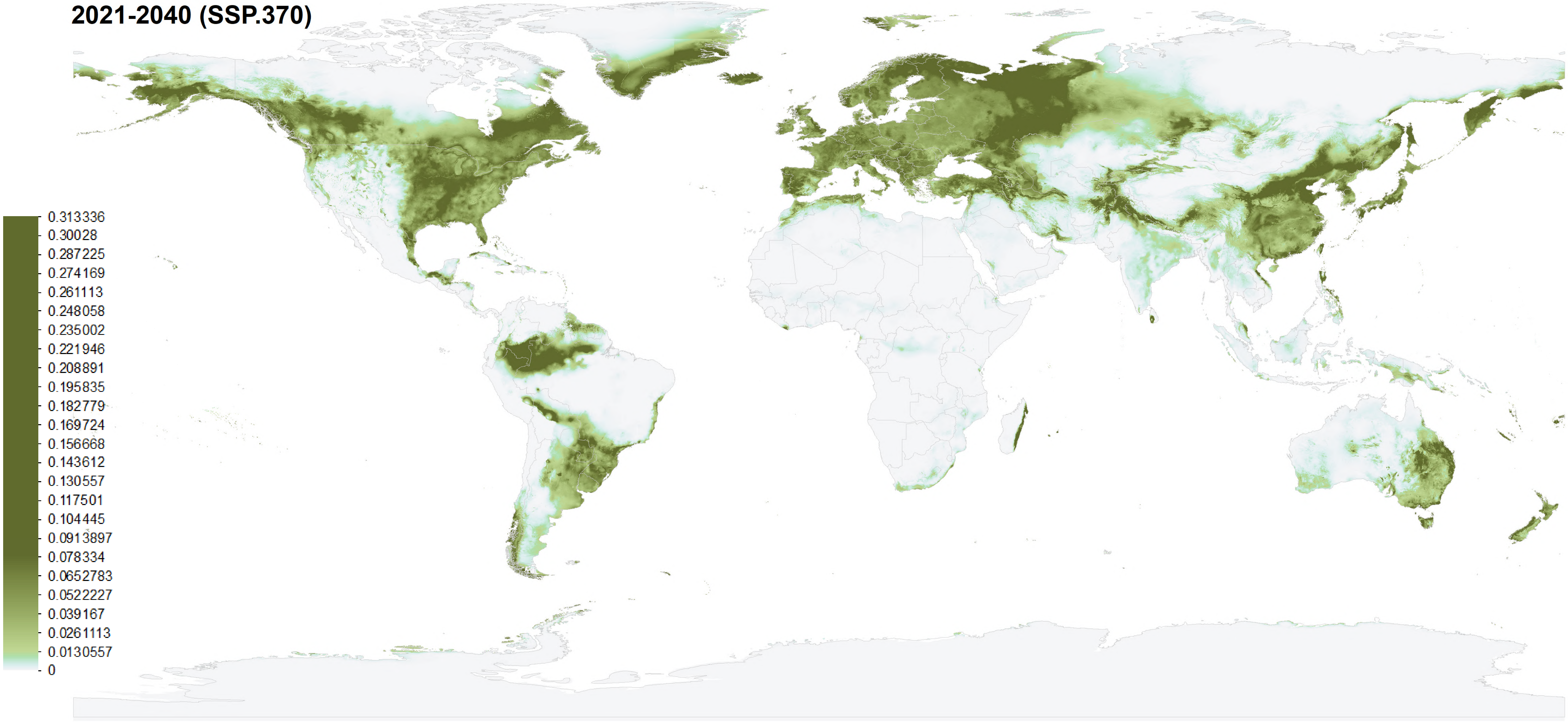

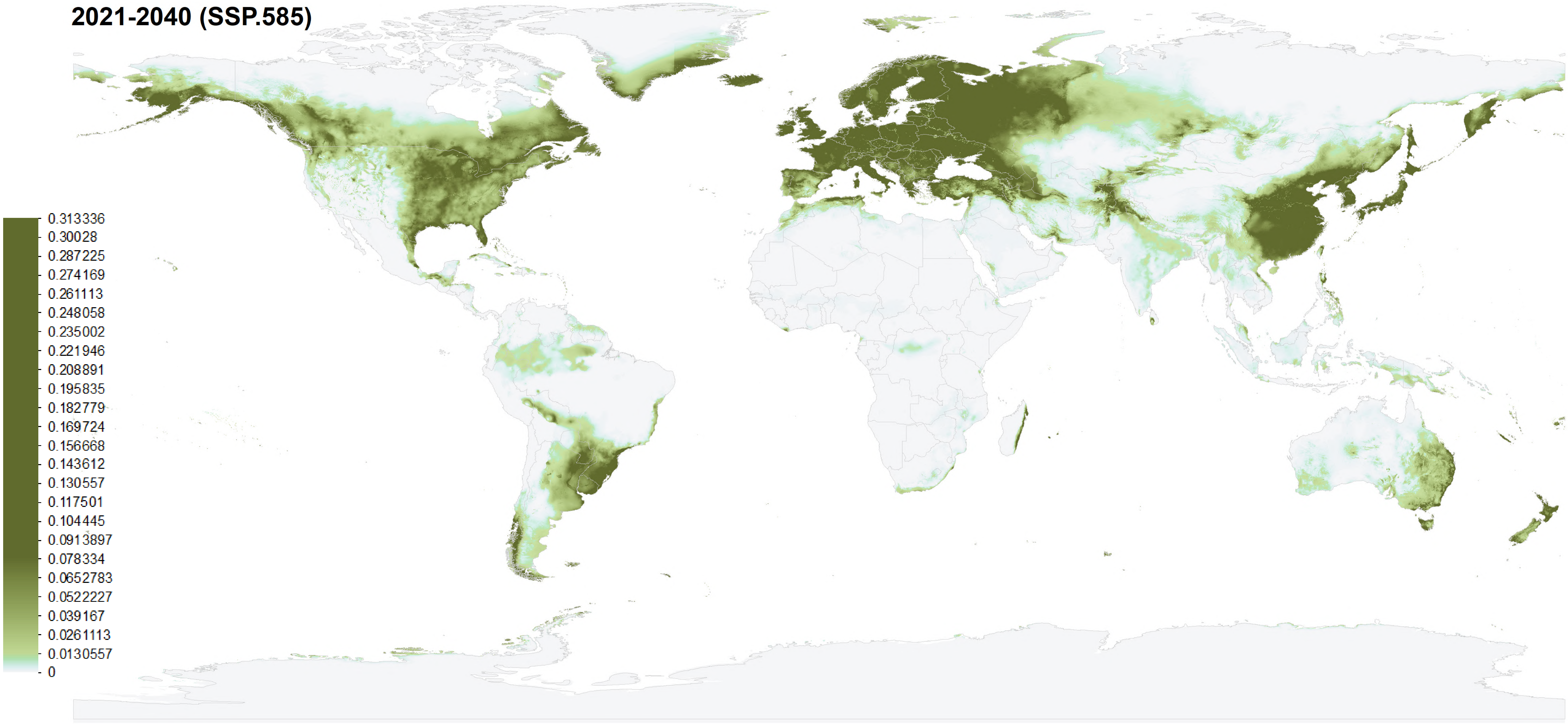
2021-2040 (SSP.585)
0.313336
0.30028
0.287225
0.274169
0.261113
0.248058
0.235002
0.221946
0.208891
0.195835
0.182779
0.169724
0.156668
0.143612
0.130557
0.117501
0.104445
0.0913897
0.078334
0.0652783
0.0522227
0.039167
0.0261113
0.0130557
0

2041-2060 (SSP.126)

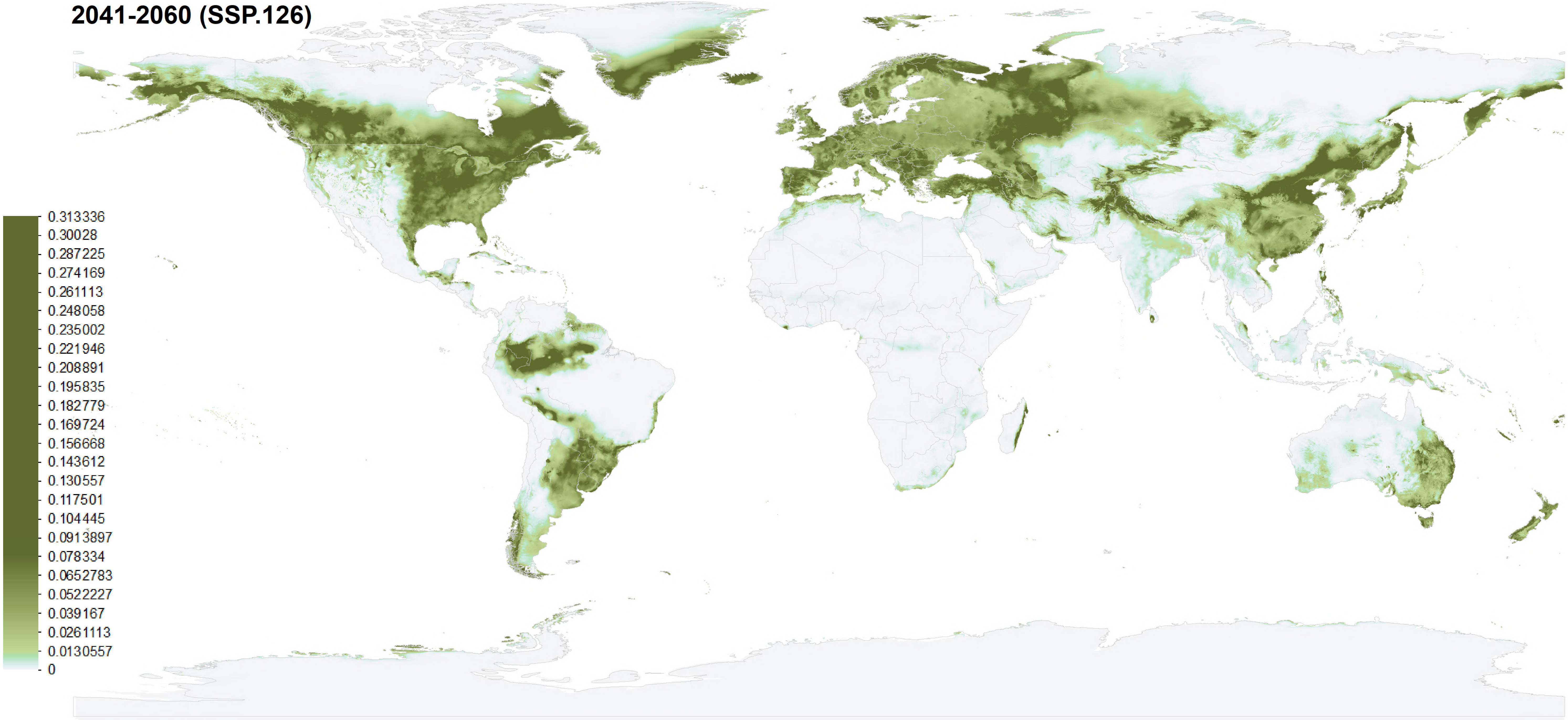

2041-2060 (SSP.245)

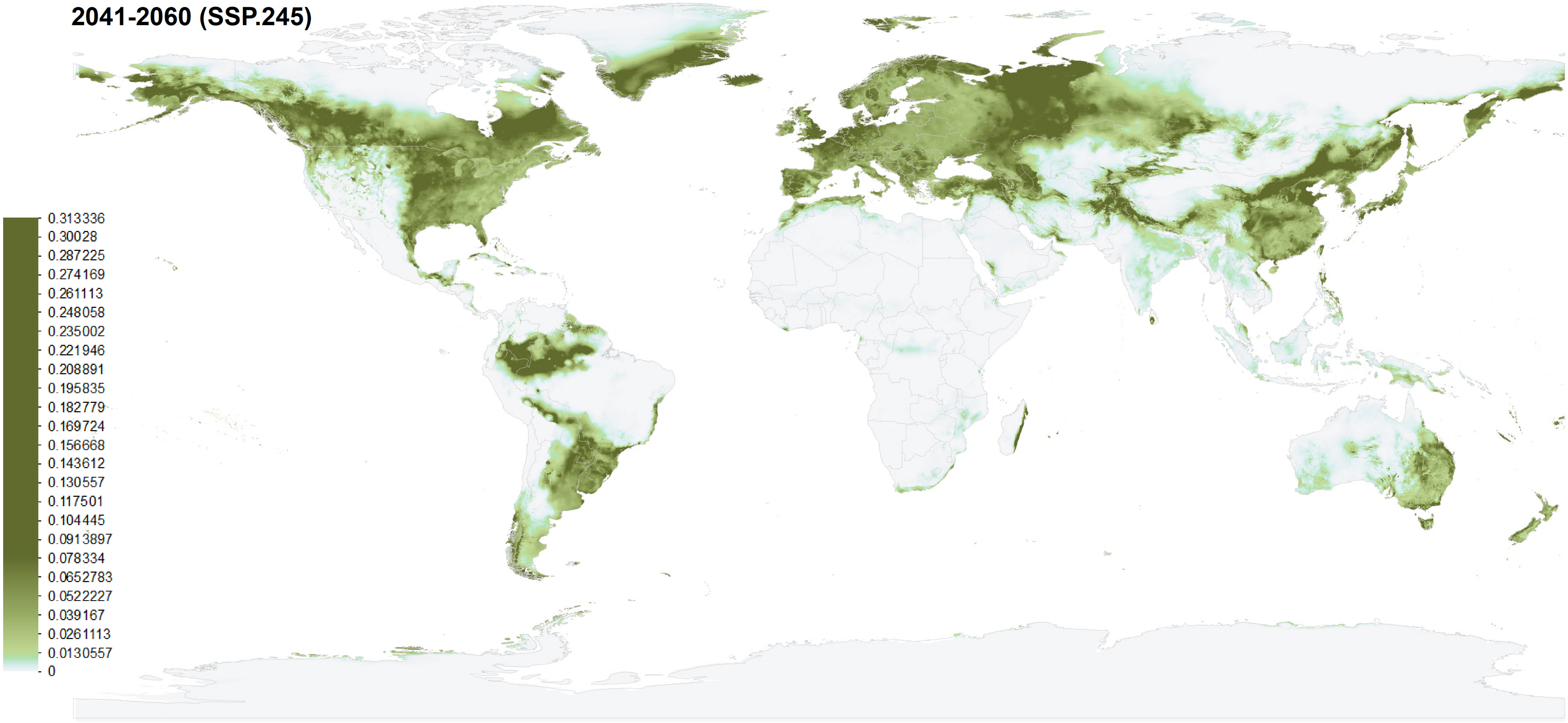

2041-2060 (SSP.370)

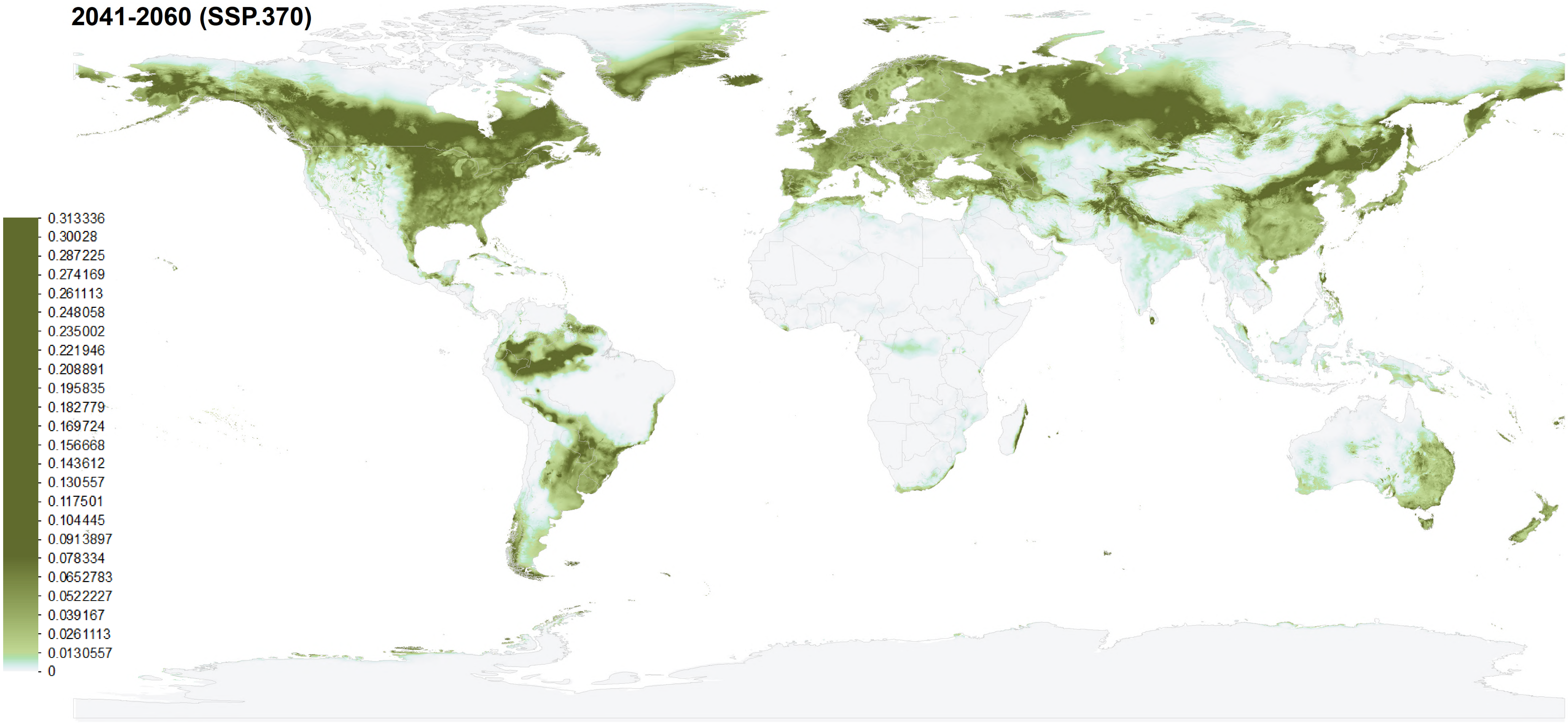

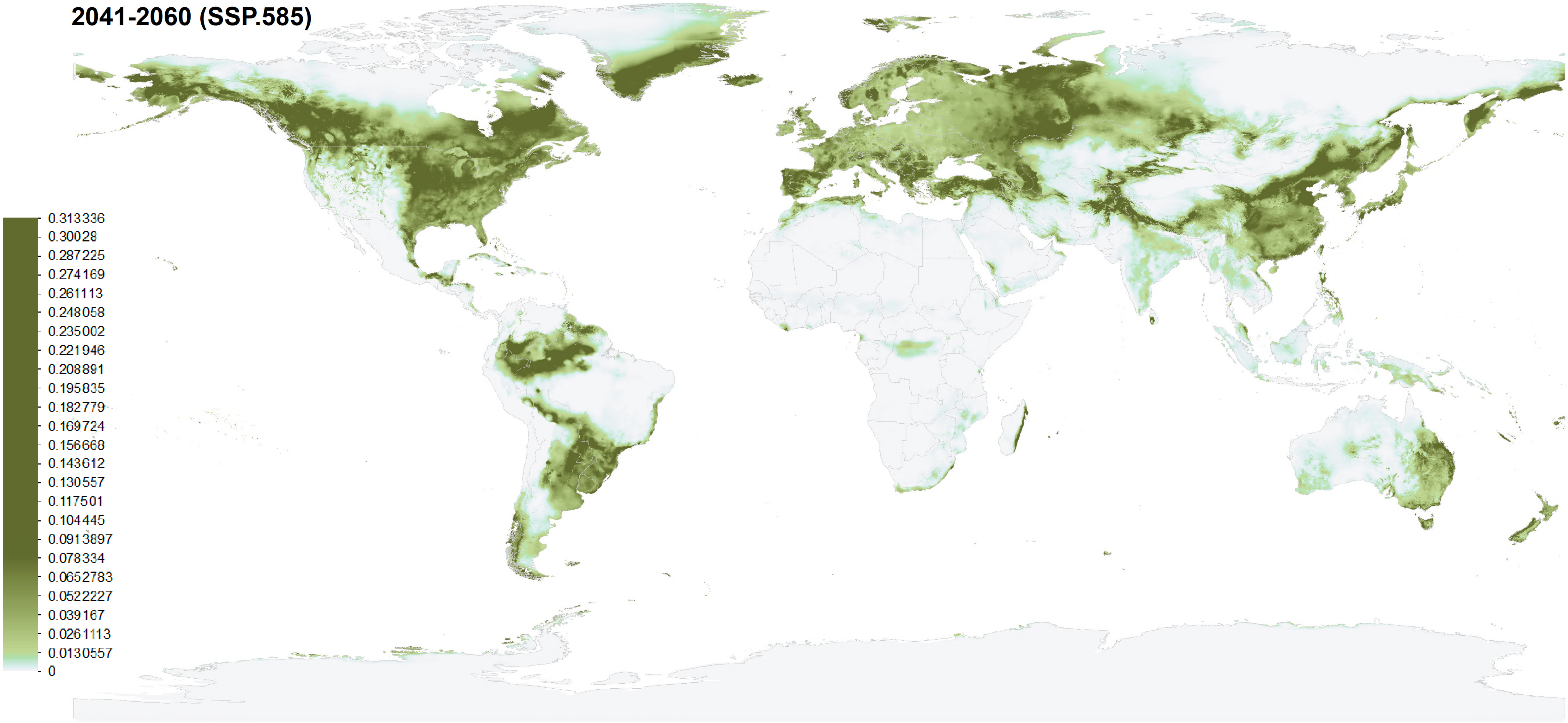
2041-2060 (SSP.585)
0.313336
0.30028
0.287225
0.274169
0.261113
0.248058
0.235002
0.221946
0.208891
0.195835
0.182779
0.169724
0.156668
0.143612
0.130557
0.117501
0.104445
0.0913897
0.078334
0.0652783
0.0522227
0.039167
0.0261113
0.0130557
0

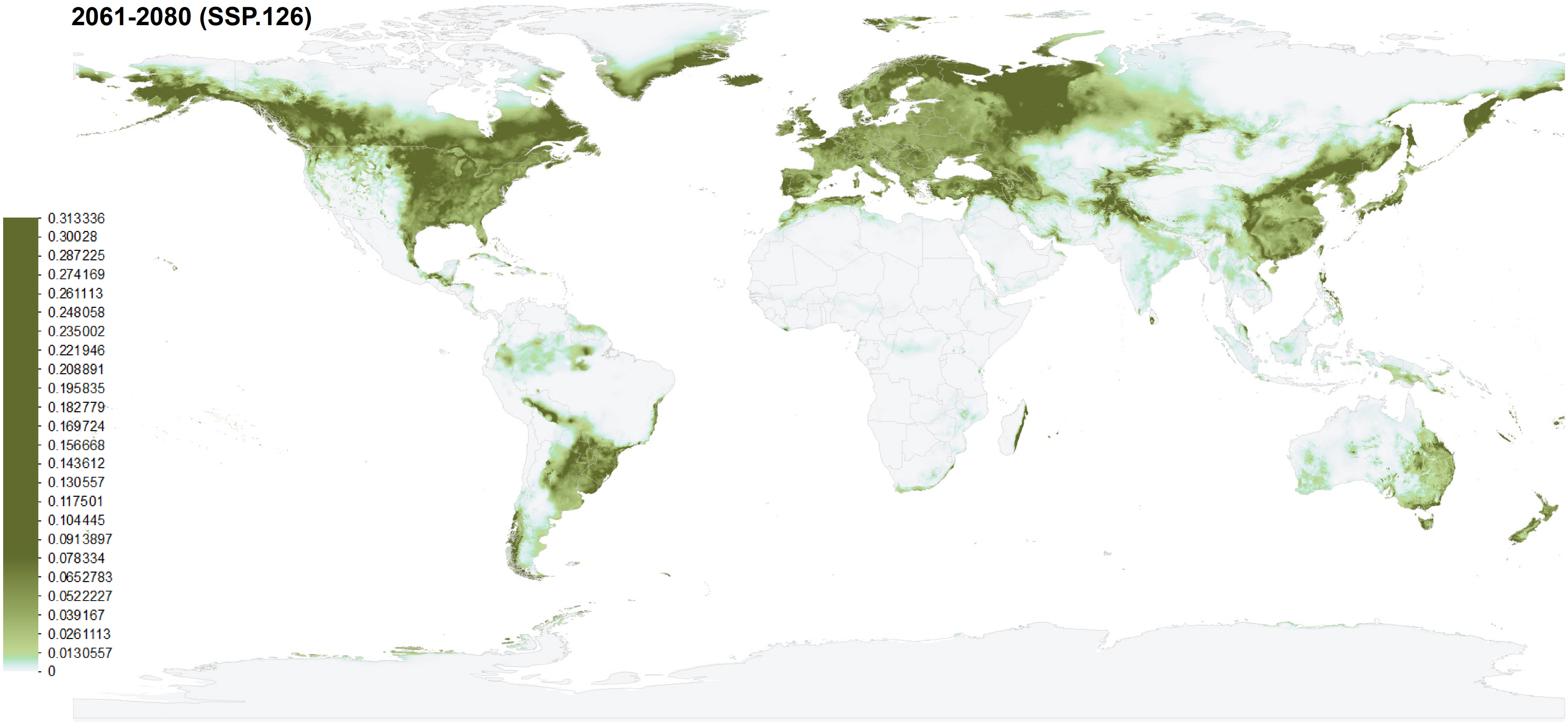
2061-2080 (SSP.126)
0.313336
0.30028
0.287225
0.274169
0.261113
0.248058
0.235002
0.221946
0.208891
0.195835
0.182779
0.169724
0.156668
0.143612
0.130557
0.117501
0.104445
0.0913897
0.078334
0.0652783
0.0522227
0.039167
0.0261113
0.0130557
0

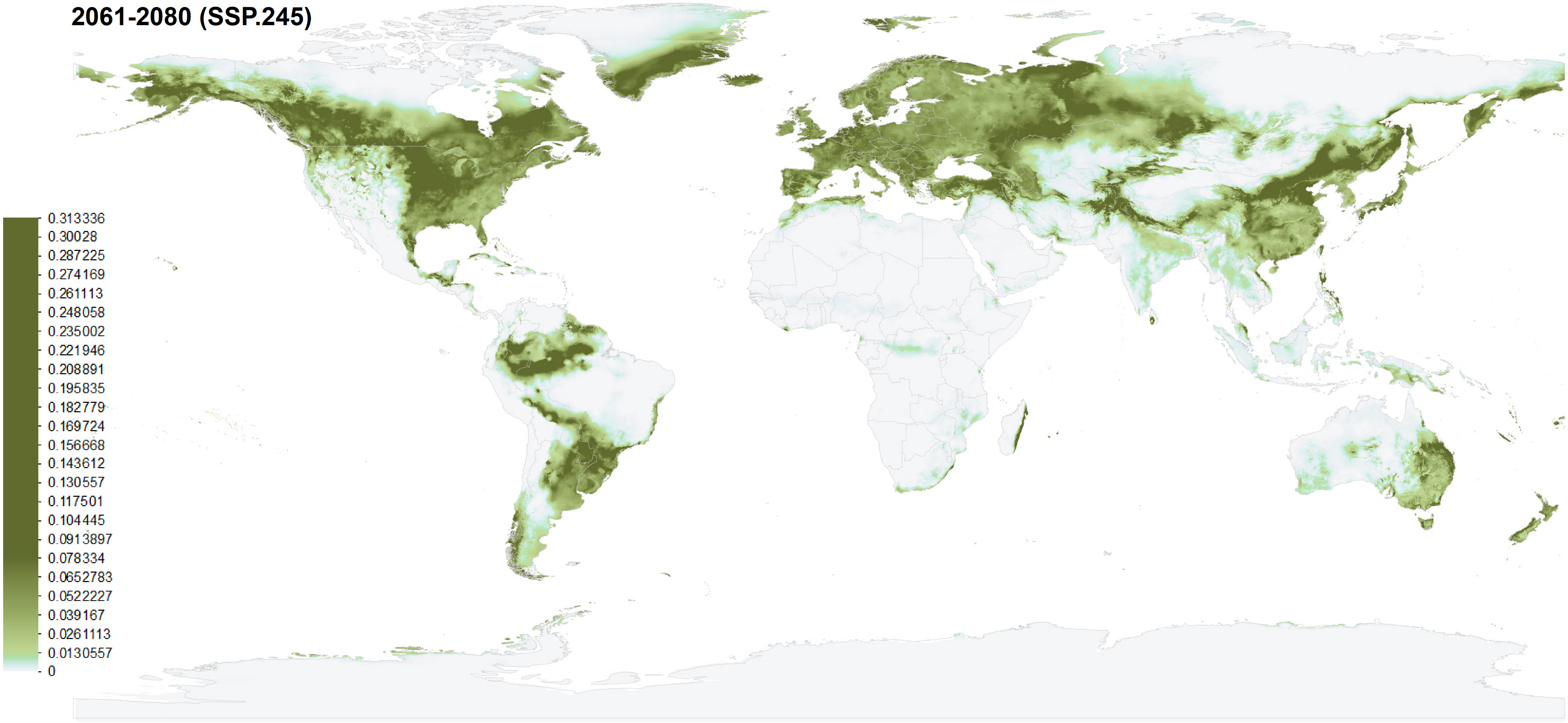
2061-2080 (SSP.245)
0.313336
0.30028
0.287225
0.274169
0.261113
0.248058
0.235002
0.221946
0.208891
0.195835
0.182779
0.169724
0.156668
0.143612
0.130557
0.117501
0.104445
0.0913897
0.078334
0.0652783
0.0522227
0.039167
0.0261113
0.0130557
0

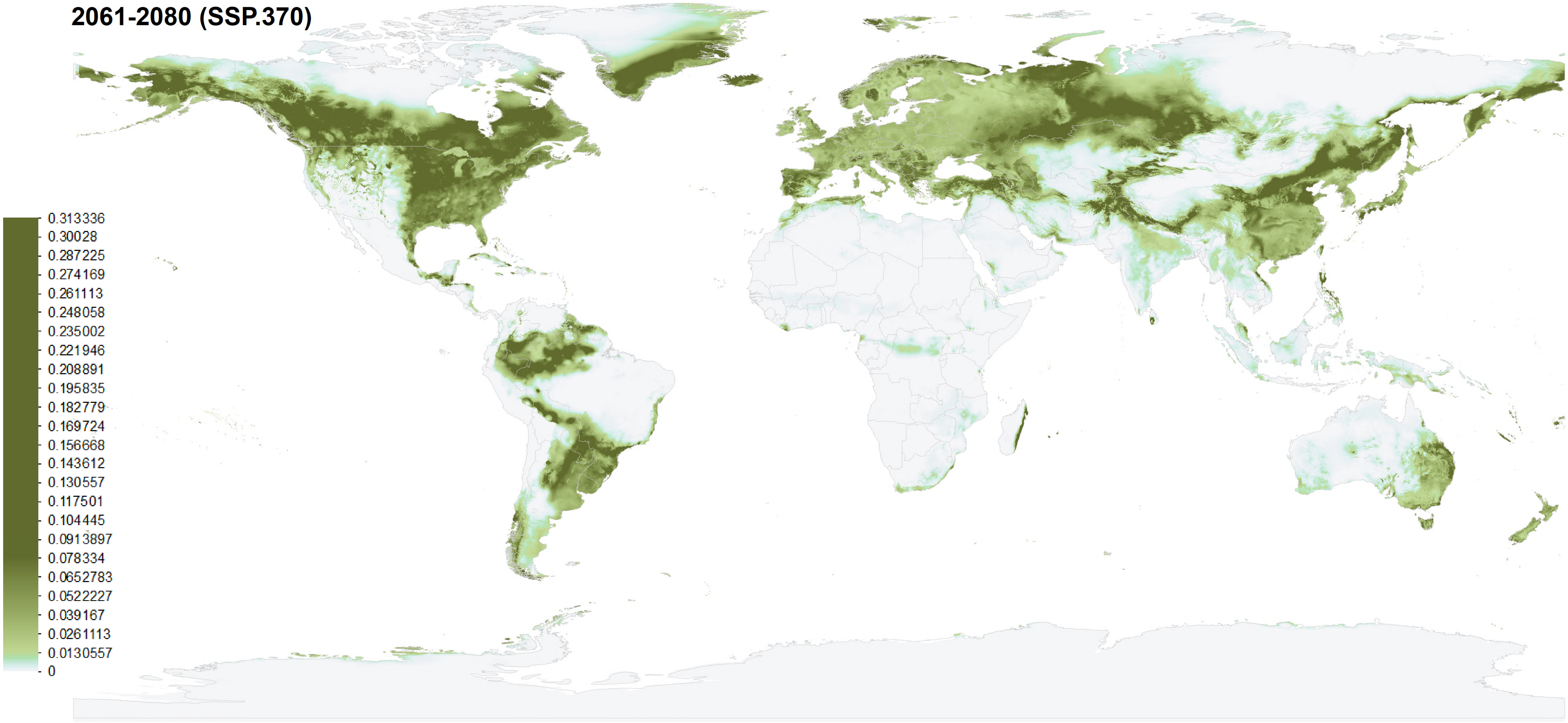
2061-2080 (SSP.370)
0.313336
0.30028
0.287225
0.274169
0.261113
0.248058
0.235002
0.221946
0.208891
0.195835
0.182779
0.169724
0.156668
0.143612
0.130557
0.117501
0.104445
0.0913897
0.078334
0.0652783
0.0522227
0.039167
0.0261113
0.0130557
0

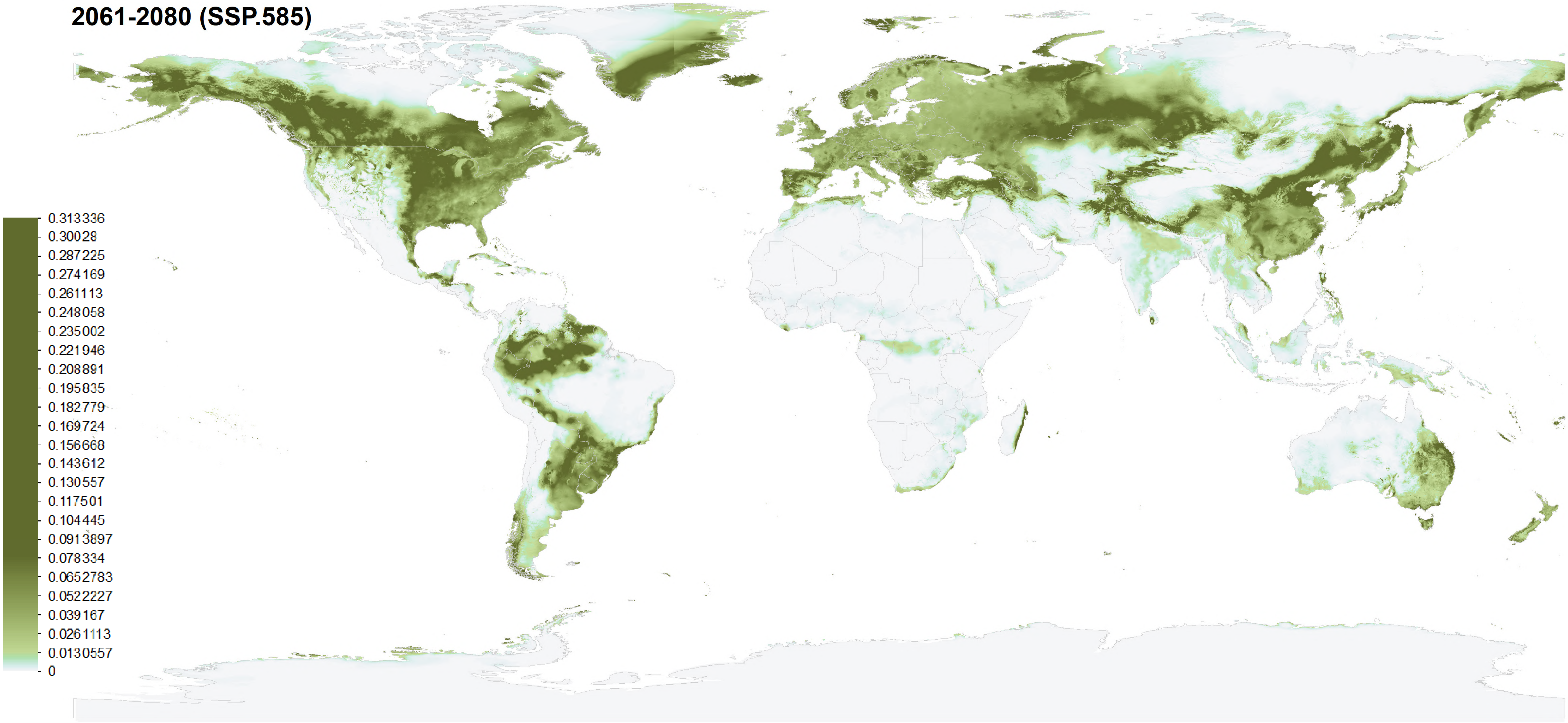
2061-2080 (SSP.585)
0.313336
0.30028
0.287225
0.274169
0.261113
0.248058
0.235002
0.221946
0.208891
0.195835
0.182779
0.169724
0.156668
0.143612
0.130557
0.117501
0.104445
0.0913897
0.078334
0.0652783
0.0522227
0.039167
0.0261113
0.0130557
0

2081-2100 (SSP.126)

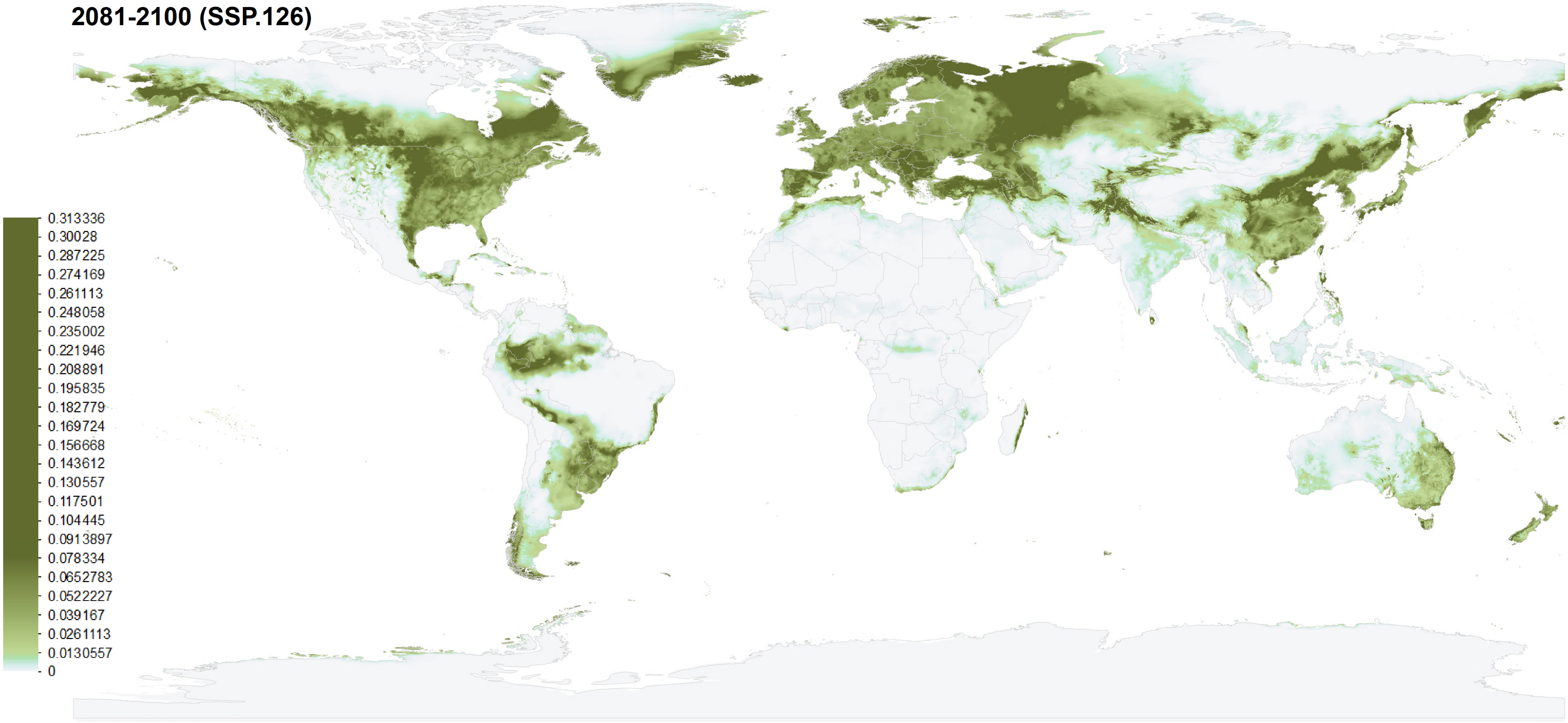

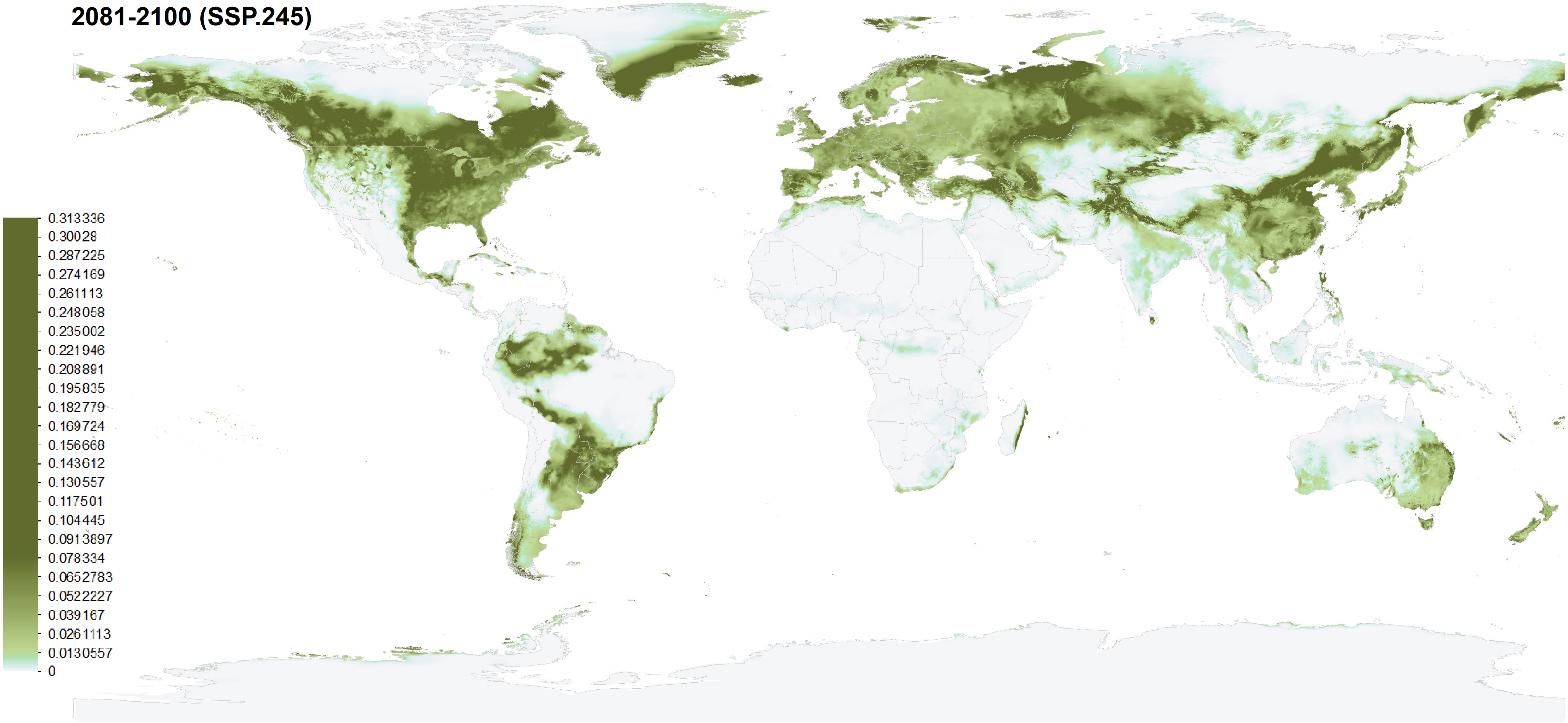
2081-2100 (SSP.245)
0.313336
0.30028
0.287225
0.274169
0.261113
0.248058
0.235002
0.221946
0.208891
0.195835
0.182779
0.169724
0.156668
0.143612
0.130557
0.117501
0.104445
0.0913897
0.078334
0.0652783
0.0522227
0.039167
0.0261113
0.0130557
0

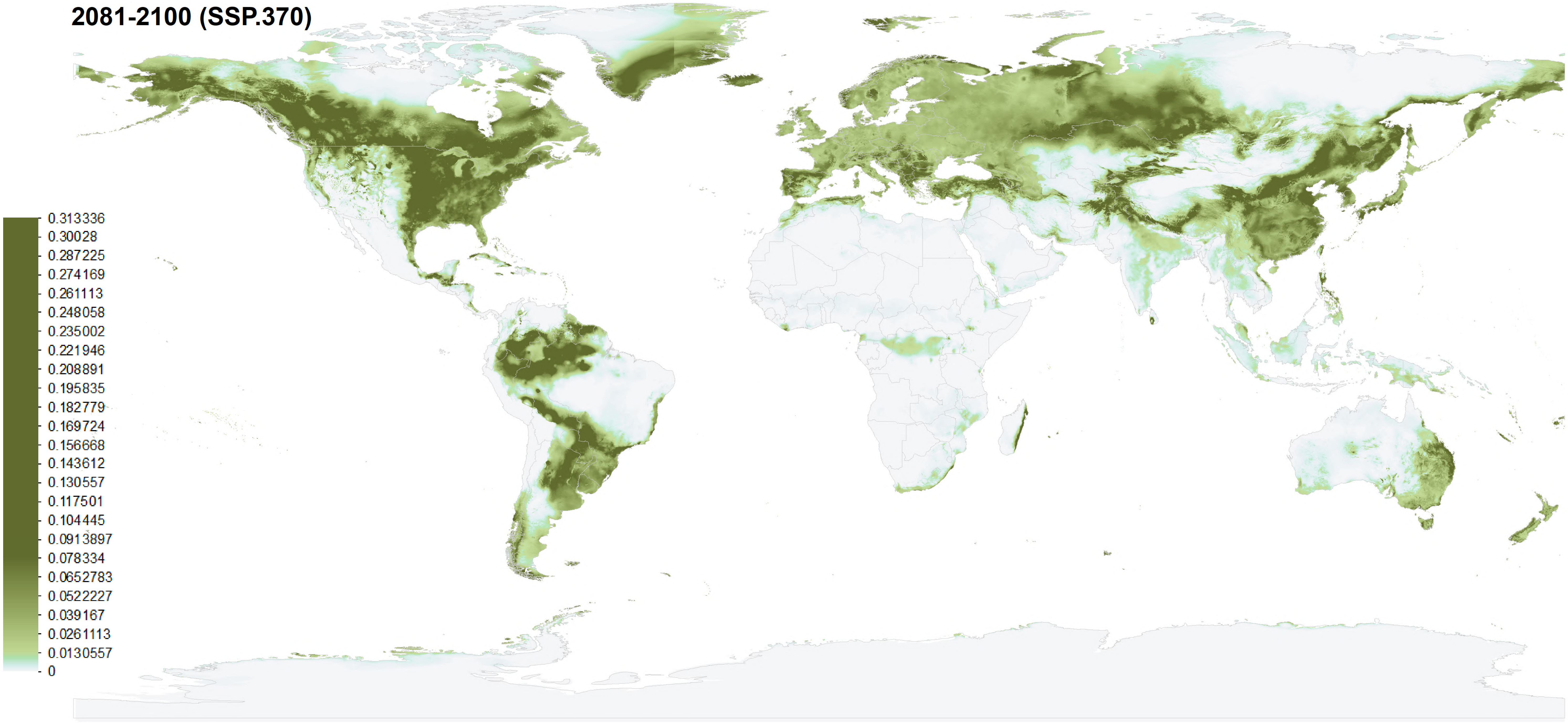
2081-2100 (SSP.370)
0.313336
0.30028
0.287225
0.274169
0.261113
0.248058
0.235002
0.221946
0.208891
0.195835
0.182779
0.169724
0.156668
0.143612
0.130557
0.117501
0.104445
0.0913897
0.078334
0.0652783
0.0522227
0.039167
0.0261113
0.0130557
0

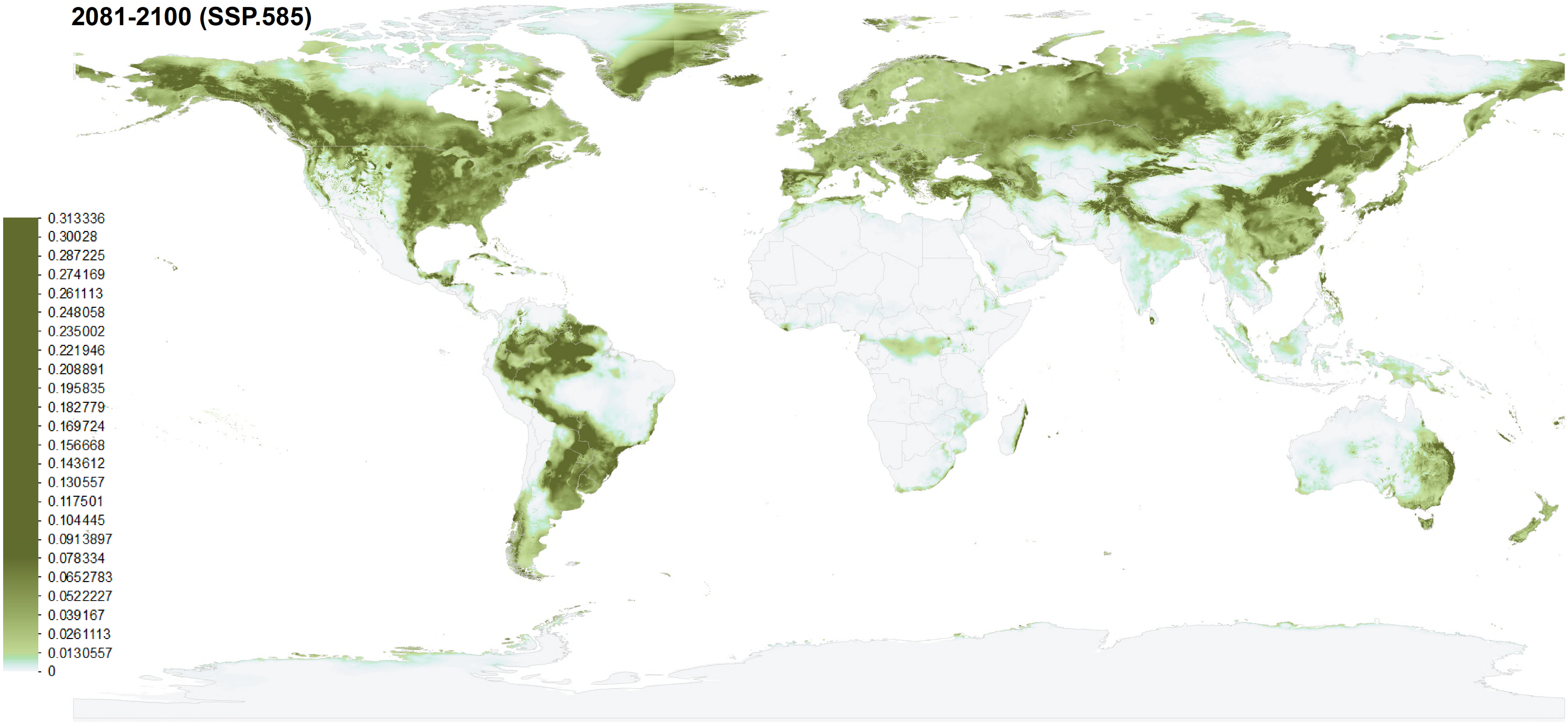
2081-2100 (SSP.585)
0.313336
0.30028
0.287225
0.274169
0.261113
0.248058
0.235002
0.221946
0.208891
0.195835
0.182779
0.169724
0.156668
0.143612
0.130557
0.117501
0.104445
0.0913897
0.078334
0.0652783
0.0522227
0.039167
0.0261113
0.0130557
0
